# Developing the structure–property relationship to design solid state multi-stimuli responsive materials and their potential applications in different fields†
†Electronic supplementary information (ESI) available: General remarks, materials, experimental details, crystallographic data, characterization data and respective spectra, computational details (DFT) including co-ordinates, and optical properties in solid and solution states. Temperature dependent studies and applications are provided. CCDC 1572548, 1536146, 1571721 and 1572060. For ESI and crystallographic data in CIF or other electronic format see DOI: 10.1039/c8sc00143j


**DOI:** 10.1039/c8sc00143j

**Published:** 2018-03-05

**Authors:** Bibhisan Roy, Mallu Chenna Reddy, Partha Hazra

**Affiliations:** a Department of Chemistry , Indian Institute of Science Education and Research (IISER) , Pune (411008) , Maharashtra , India . Email: p.hazra@iiserpune.ac.in; b Centre for Energy Science , Indian Institute of Science Education and Research (IISER) , Pune (411008) , Maharashtra , India

## Abstract

Prediction of multi-stimuli responsive behavior in newly developed luminogens is an appealing yet challenging puzzle, since no concrete design strategy has been developed so far. In this article, we demonstrate a potent strategy to gain a deep understanding of the structure–property relationship to design multi-stimuli responsive mechanochromic materials. To achieve our goal, a variety of new isoindolinone core based charge transfer luminogens exhibiting aggregation-induced emission (AIE) have been prepared through C–H bond activation using a cost-effective ruthenium (Ru) metal catalyzed one-pot synthetic strategy. We have shown that slight tuning of the donor moiety is found to be highly effective in controlling molecular packing and metastable energy states in solid states, and thus, optical properties and multi-stimuli responsive behaviors. The flexibility and twisting of donor moieties afford a loosely bound ‘herringbone’ packing, enabling reversible transformation under multiple mechanical stimuli. The cyclized derivative of the donor exhibits a completely different packing mode (*i.e.*, cross packing), and subsequently, does not give rise to mechanochromism. The Hirshfeld surface analysis from a single crystal infers that non-covalent interactions (specifically C–H···π and π···π) are extremely important to yield mechanochromism under external force. Correlating all solid-state behavior with the molecular structure, we conclude that the synergistic effect between the twisting and conformational flexibility of donor moieties along with numerous non-covalent interactions gives rise to multi-stimuli responsive behaviors. Finally, the newly designed molecules are found to be highly emissive in solution and potentially applicable in fluorescence thermometer construction, lighting up cells, acid–base sensors and rewritable devices.

## Abstract 2

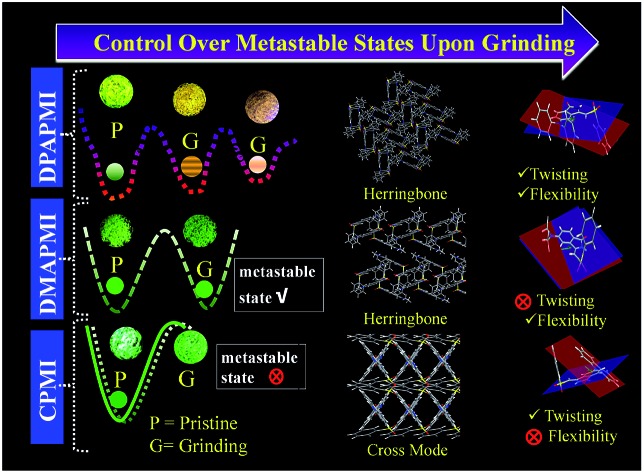
Establishing the structure–property relationship for multi-stimuli responsive mechanochromic materials based on charge transfer luminogens.

## Introduction

Luminescent materials (or luminogens) sensitive to mechanical stimuli have attracted burgeoning interest owing to their promising applications in optical storage, mechanical sensors, security systems, optoelectronic devices, *etc.*^1^–^14^ The mechanochromic properties of organic molecules are mainly governed by the molecular packing in the condensed state.^7^,^8^,^12^,^15^–^37^ However, most of the luminogens in the condensed state suffer from the destructive aggregation-caused quenching (ACQ) effect.^38^,^39^ Thus, it is a highly onerous job to precisely fabricate mechanochromic materials with significant emission efficiency. Until now, the development of most mechanochromic materials has strongly depended on the serendipitous discovery or derivatization of known mechanochromic organic cores;^7^,^8^,^12^–^24^ hence, the majority of the mechanochromic events often appeared as a single isolated event.^3^,^16^ Collective mechanochromic luminogens apparently suggest that non-covalent interactions may play an important role in the appearance of this novel phenomenon.^7^,^8^,^12^–^24^,^40^ However, an in-depth and comprehensive understanding of non-covalent interactions is still lacking. Therefore, it is important to develop a novel strategy, which can provide a deep insight into the understanding of the structure–property relationship for developing new mechanochromic materials. Very recently, it has been established that the donor (D)–acceptor (A) substituent of small organic molecules can induce molecular packing, thereby modulating the mechanochromic properties of the luminogens.^41^,^42^ Moreover, it has been reported that D–A molecules having locally excited (LE) and charge transfer (CT) states are beneficial for improved efficiency of electroluminescent devices,^43^ and this kind of molecular system is useful for high contrast reversible fluorescence tuning driven by a switching of the excited state in the solid phase under mechanical stimuli. However, the biggest challenge to construct mechanochromic materials based on the D–A skeleton is the densely packed arrangement (mostly as head to tail driven by the oppositely charged character in D–A molecules) owing to their well-separated electron density.^44^,^45^ The densely packed arrangements in the solid state suppress the possibility of mechanochromism, as such packing is unable to produce metastable states under external mechanical force. Therefore, establishment of the structure–property relationship based on CT luminogens along with an inherent molecular level understanding of mechanochromism undoubtedly paves a new way to design this type of novel material.

**Scheme 1:**
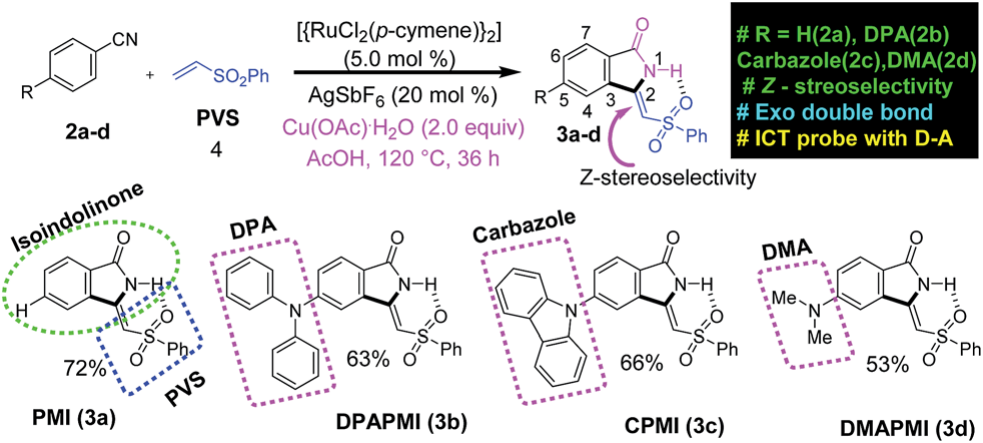
Ruthenium-catalysed one pot synthetic route to isoindolinone based new CT luminogens.

In this article, we have invested much effort to provide a structure–property relationship to design mechanochromic materials based on the precise tuning of solid-state packing by modulating donor substitution in isoindolinone (green circle part in Scheme 1) based newly developed charge transfer (CT) luminogens. We have noticed that slight tuning of donor substitution in CT luminogens can effectively control the metastable states under mechanical grinding. We have also observed that multiple non-covalent interactions play a crucial role in obtaining mechanochromism. Moreover, it is found from Hirshfeld surface analysis (from single crystal data) that, among various non-covalent interactions, precisely C–H···π and π···π interactions dictate the mechanochromism of the D–A based CT luminogens. In addition, by tuning the donor units, we have shown that not only the twisting but also the flexibility of donor units in CT luminogens is crucial to obtain mechanochromism under external stimuli. It must be pointed out that individually none of the above-mentioned parameters are able to provide mechanochromic properties. Hence, we conclude that the synergistic effect between the twisting and conformational flexibility of donor units along with numerous non-covalent interactions (especially C–H···π and π···π interactions) gives mechano-active properties to CT luminogens. Our study also illustrates an idea regarding the design of self-reversible mechanochromic materials. To the best of our knowledge, this is the unique report providing a detailed insight regarding the structure–property relationship with precise control of metastable energy states based on the tuning of molecular arrangement and Hirshfeld surface analysis. Interestingly, the newly developed CT luminogens show strong emission, and emission peak positions are found to be strongly dependent on the polarity of the solvent. Notably, all of our designed luminogens exhibit gigantic emission shifts of ∼125 nm (DPAPMI), ∼120 nm (CPMI) and ∼100 nm (DMAPMI) going from low to high polarity solvents along with the fluorescence switching ability over a wide range of temperatures. We have employed such temperature dependent fluorescence switching in the applications of fluorescence thermometer construction. Our designed molecules were also found to exhibit the potential ability to be applied in lighting up cells and rewritable devices.

## Results and discussion

### Molecular design

According to the focus of this work, we have incorporated three common frameworks in each molecule: (1) D–A framework, (2) twisted conformation of the donor moiety and (3) multiple non-covalent interaction sites. In addition, attention has been paid to the tuning of the donor moiety to control the flexibility and molecular packing. After a careful search, we have selected isoindolinone as the core moiety for this work. Isoindolinone, a typical planar, electron deficient molecule, consists of a fused phenyl and pyrrolidinone ring, and hence, it may be well suited for the π···π stacking. A slight modification of the five-membered heterocyclic pyrrolidinone ring of isoindolinone has been performed by introducing a phenyl vinyl sulfone (PVS) group (blue square region in Scheme 1), which may be useful here for the following reasons. (1) The sulfone group in PVS will form a strong intramolecular H-bond with the N–H of the isoindolinone core forming a fused molecular framework with *Z*-stereoselectivity (discussed in the SCXRD section), which is necessary for the π···π stacking. (2) The bulky phenyl ring in PVS can play a significant role in keeping the balance between two sets of molecular planes. (3) The sulfone unit in PVS may also offer strong intermolecular contacts by robust hydrogen bond formation to immobilize the molecular conformations and rigidify the crystal, thereby, reducing non-radiative decay channels. However, the PVS attached isoindolinone framework (abbreviated as PMI in Scheme 1) is still electron deficient in nature (shown by DFT calculations in the next section); hence, it can act as an efficient acceptor (A). Next, we plan to attach some twisted donor moiety at the 5^th^ position on the fused phenyl ring of PMI, as it will allow electron flow from the donor moiety to the carbonyl side (Scheme S1†). Notably, donor substitution at the 6^th^ position creates electron flow towards the sulfone side, which may possibly destabilize the *Z*-conformation of the luminogens (Scheme S1†). Now, the most crucial job in the design part is the proper selection of the donor group at the 5^th^ position (pink circle part in 3a–d in Scheme 1). According to our target, a propeller-shaped (twisted) and flexible donor moiety is anticipated to provide flexibility in the crystal packing, and may exhibit tunability under external mechanical stress. Next, a cyclized framework of that same flexible donor moiety needs to be synthesized in order to understand the importance of flexibility in mechanochromism. In such a context, propeller-shaped triphenylamine (TPA) would have been an ideal choice owing to its flexibility and twisted conformation (CNC angle 119.6°).^46^ However, synthesizing a cyclized framework of TPA is virtually impossible. Thus, we have chosen its close analogue, *i.e.*, diphenylamine (DPA), which exhibits flexibility, and twisting nature (CNC angle 123.9°).^47^ Moreover, the cyclized analogue of DPA, *i.e.*, carbazole is a well-known moiety having interesting material properties, such as charge transport, luminescence and thermal stability.^48^ Besides this, we have also chosen dimethylamine (DMA) as a donor moiety to clearly understand the influence of conformational twisting on mechanochromism. Notably, DMA is the smallest in size but strongest in donor ability compared to DPA and carbazole.

### Synthesis and characterization

The traditional way of synthesizing the designed molecules would suffer from multistep procedures and hard accessibility of synthetic precursors. Here, we have adopted a cost-effective, metal (ruthenium) catalyzed, step economical one-pot synthetic procedure using C–H bond activation as a key step. To the best of our knowledge, this is the first ever report on synthesizing mechanoactive molecules based on Ru metal catalyzed C–H bond activation. A brief representation of the synthetic scheme and mechanism is outlined in Schemes 1 and S2,† respectively. According to our aim, the parent and donor substituted luminogens are formed in a highly *Z*-stereoselective manner (Scheme S2†). In brief, the substituted benzonitrile starting materials (2b and 2c) were synthesized by a nucleophilic fluoro-displacement reaction of 4-fluorobenzonitrile with diphenylamine and carbazole (for details see ESI†). The functional group interconversion (FGI) followed by the oxidative cyclization of benzonitrile with phenyl vinyl sulfone (4) was performed in the presence of a Ru metal catalyst {RuCl_2_(*p*-cymene)}_2_ (5.0 mol%), silver salt (AgSbF_6_ (20 mol%)), Cu(OAc)_2_, and H_2_O (2.0 equiv.) in acetic acid (AcOH) solution at 120 °C for 36 h (for details see ESI†). This powerful *in situ* synthetic route results in the cyclization product (*Z*)-3-((phenylsulfonyl)methylene)isoindolin-1-one (PMI) (3a) selectively as a *Z*-stereoisomer with high product yield (72%). In the same manner, substituted benzonitrile (2b–d) efficiently undergoes an *in situ* cyclization reaction with PVS (4) resulting in (*Z*)-5-(diphenylamino)-3-((phenylsulfonyl)methylene)isoindolin-1-one (DPAPMI), (*Z*)-5-(9*H*-carbazol-9yl)-3-((phenylsulfonyl)methylene)isoindolin-1-one (CPMI), and (*Z*)-5-(dimethylamino)-3-((phenylsulfonyl)methylene)isoindolin-1-one (DMAPMI) (3b–d) with 63%, 66% and 53% product yield, respectively (for details see ESI†). Each synthesized starting material and each final product have been purified by silica-gel column chromatography using hexane and ethyl acetate as the eluent (4 : 1 for starting materials and 9 : 1 for final products). All the starting materials and final products have been extensively characterized by ^1^H, ^3^C, DEPT-135 NMR, HRMS and IR spectroscopic studies (see the Characterization section in the ESI†). In addition to this, single crystal X-ray diffraction has been provided as a characterization proof. The solubility test indicates that the synthesized compounds show good solubility in all common organic solvents.

### Density functional theory calculations

**Fig. 1:**
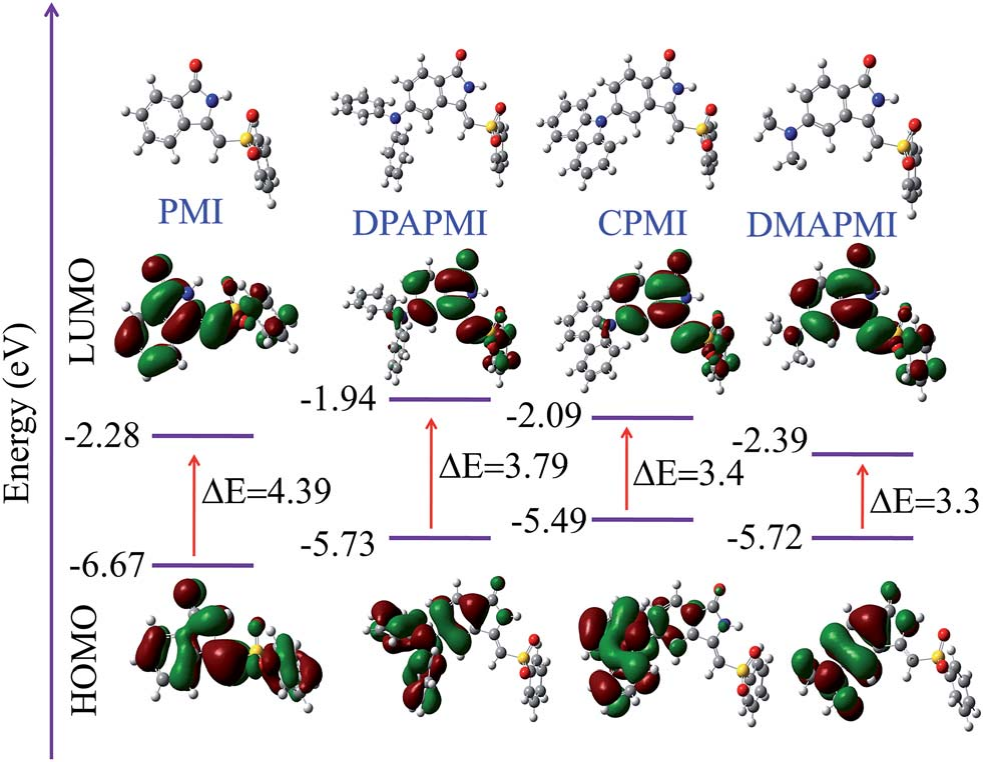
HOMO and LUMO orbitals along with energy levels of PMI and its donor substituted derivatives (optimised geometry of each molecule has been given).

To assess the viability of the molecular design and the electronic effect of donor substituents on the parent PMI molecule, we have conducted density functional theory (DFT) calculations at the B3LYP/6-31G (d,p) level (see ESI† for co-ordinates). The calculated highest occupied molecular orbital (HOMO), lowest unoccupied molecular orbital (LUMO) and optimized geometries along with the energy diagram are summarized in Fig. 1. In PMI, the electron densities of HOMO and LUMO orbitals are uniformly distributed. However, the electron densities of HOMO and LUMO orbitals in donor substituted PMI are completely separated. The electron density in the HOMO is located on the donor group (DPA, carbazole and DMA), while the LUMO orbital resides at the core of the PMI moiety in each case. Hence, upon photoexcitation there must be a considerable intramolecular charge transfer (ICT) from D to A. It is clear from the energy diagram that donor groups push the HOMO level up, whereas they hardly influence the energy level of the LUMO. As a result, the energy gap (Δ*E*) of donor substituted molecules narrows down compared to the parent PMI molecule. The calculated HOMO energies of DPAPMI, CPMI and DMAPMI are –5.73, –5.49 and –5.72 eV, respectively.

### Optical properties in THF solvent and aggregation-induced emission study

We have measured the optical properties of donor substituted PMI derivatives in a medium polar solvent THF (Δ*f* = 0.208) to assign the LE and CT emission peaks (Fig. S1†). The parent PMI molecule exhibits absorption and emission maxima at ∼315 nm and 430 nm, respectively in THF. As PMI does not have a donor moiety, it emits only from the LE state. All other donor substituted derivatives exhibit two absorption peaks in THF; one in the UV region at ∼315 nm and the other in the visible region between 380 and 415 nm (Fig. S1†). The absorption band located in the UV region can be assigned to the π–π* electronic transition for the PMI molecule and the band appearing in the visible region (380–415 nm) is attributed to a newly generated CT transition from donor moieties (DPA, carbazole, and DMA) to the acceptor (PMI). The emission spectra of the donor conjugated PMI molecules exhibit a dual emission peak arising from LE (higher energy peak) and CT (lower energy peak) states. For CPMI, the intensity of the CT peak is stronger than that of the LE peak (appearing as a shoulder peak); on the other hand the intensity of the LE peak is higher than that of the CT peak in the case of DPAPMI. This observation can be rationalized in terms of different ratios of populations of CT and LE states of these two molecules in THF. However, DMAPMI exhibits a single emission peak at ∼505 nm corresponding to a stabilized CT energy state in THF (Fig. S1†), which is probably because of the strong donor ability of the DMA moiety.

**Fig. 2:**
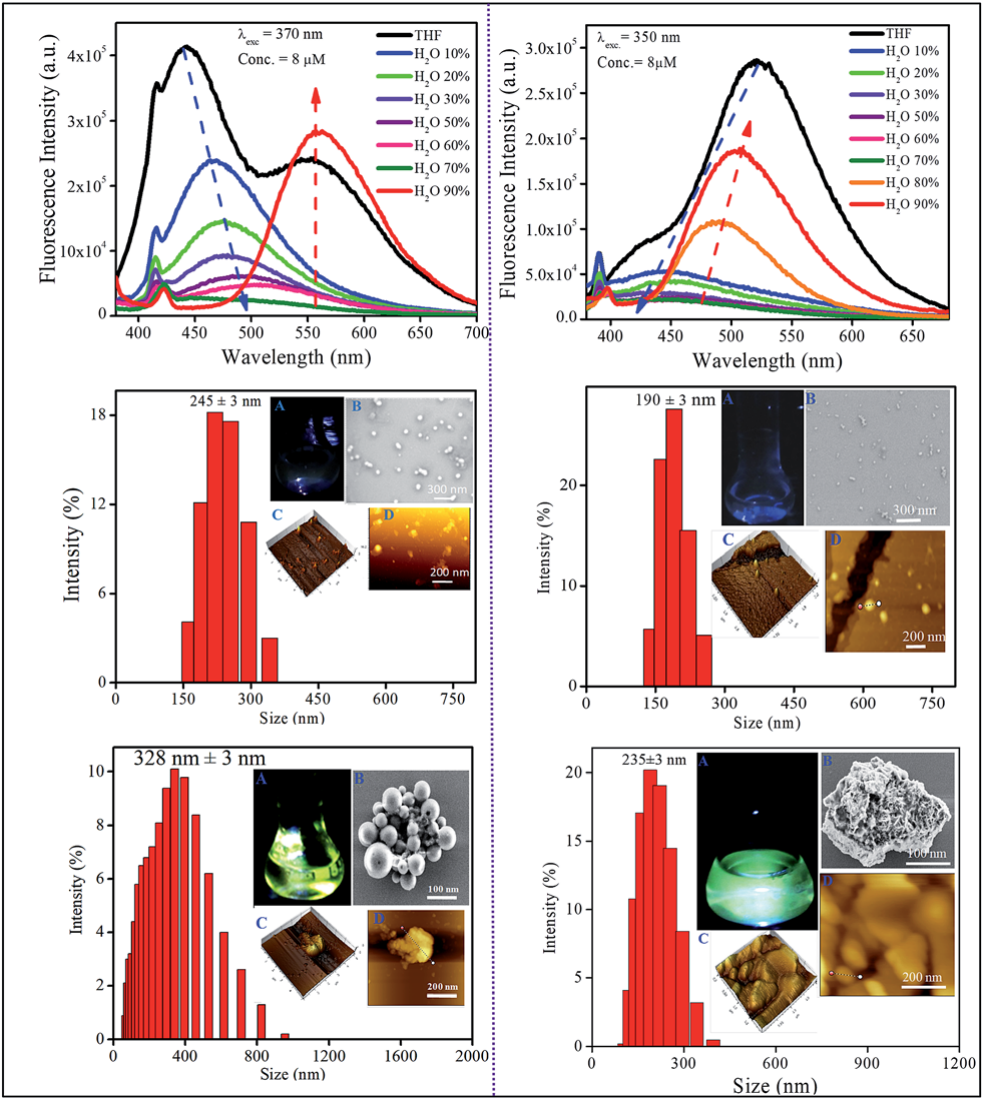
Aggregation-induced emission (AIE) results of DPAPMI (left column) and CPMI (right column). Steady-state emission spectra (top row), and characterization (DLS, SEM and AFM) at a low water (10%) content (middle row) and a high water (90%) content (bottom row). Here, A, B, C, and D represent the vial image (under 365 nm UV), SEM, AFM (3D height), and AFM morphology respectively in each case.

Prior to investigating the emission behavior in the solid state, the optical properties of nano-aggregates have been studied in THF/water binary mixtures. Since the molecules are not soluble in water, they should aggregate in the binary mixtures at high water content, and we are interested to see how aggregation affects the emission properties of CT luminogens. It has been mentioned that PMI and its *N*,*N*-dimethyl derivative, *i.e.*, DMAPMI emit at 430 nm (LE peak) and at 505 nm (CT state), respectively in bulk THF. Upon gradual addition of water (poor solvent) into the THF, the emission becomes gradually weaker for both PMI and DMAPMI with a concomitant redshift of ∼45 nm only for DMAPMI (Fig. S2†). At a very high water content (>95%), almost negligible fluorescence is observed for both the compounds. However, for DPAPMI and CPMI exactly opposite but interesting observations were found. Although both molecules exhibit dual emission peaks (LE and CT) in THF solution, with slight addition of water, the CT peak vanishes completely (Fig. 2). Interestingly, the LE peak persists with diminished intensity, and this trend continues before their emission becomes the ‘off’ state prior to *f*_w_ = 70% (Fig. 2). Interestingly, above *f*_w_ = 70%, unlike PMI and DMAPMI, the emission is invigorated from the CT peak for both the luminogens along with a redshift, and this increase in PL intensity after *f*_w_ = 70% can be attributed to the aggregation induced effect (AIE). The formation of nano-aggregates is confirmed by DLS, FE-SEM and AFM studies (Fig. 2 and S2†).

Owing to its poor solubility, PMI has a tendency to aggregate in water, and in the aggregated state it becomes non-emissive as a result of strong π···π stacking interactions between highly planar PMI moieties. Consequently, PMI exhibits aggregation-caused quenching (ACQ) at higher water content like normal rigid fluorophores. Surprisingly, despite the presence of DMA substitution, DMAPMI shows ACQ nature in the aggregated state. This observation suggests that the smaller size of the DMA group is not sufficient enough to disturb the π···π stacking interactions between the PMI moieties in the aggregated state. For DPAPMI and CPMI, probably the twisting conformations (due to the presence of bulky DPA groups and the carbazole moiety as a donor for DPAPMI and CPMI, respectively) of the molecules do not allow them to be involved in effective stacking interactions in the aggregates. This is also evident from the crystal-induced enhanced (CIE) emission observed in the condensed state for both the compounds. Generally, the intramolecular rotations decrease the emission efficiency from the CT state in bulk solution medium, whereas in the CT process in the aggregate, the intramolecular rotation is restricted, thereby, causing an increase in the efficiency of CT emission. As a result, the emission from the CT state gets a boost by the aggregation induced emission (AIE) process for both DPAPMI and CPMI molecules.

### Single crystal X-ray diffraction (SCXRD) study and optical properties in the crystalline state

**Fig. 3:**
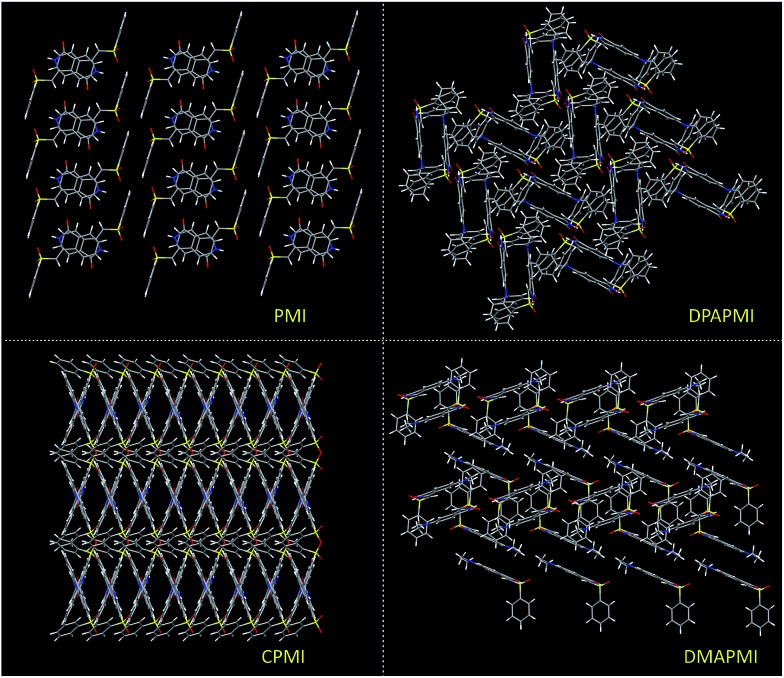
Molecular packing modes of PMI (lamellar), DPAPMI (herringbone), CPMI (cross mode) and DMAPMI (herringbone) luminogens.

**Fig. 4:**
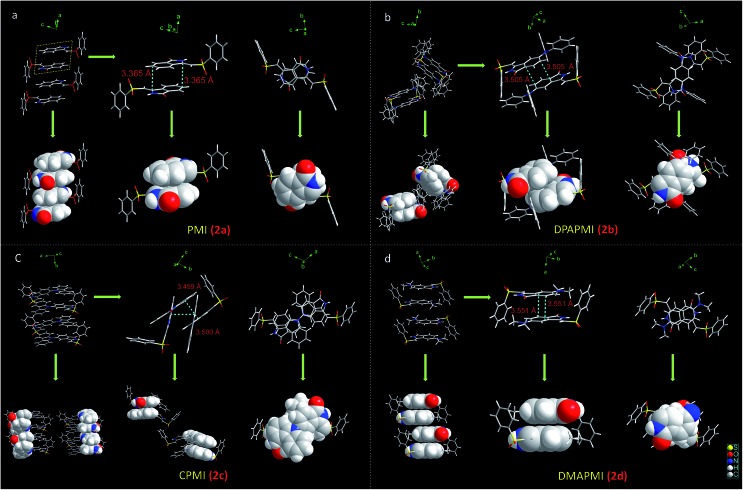
Molecular level understanding of π···π stacking interactions of PMI and donor substituted derivatives. The space-filling model has been shown for better clarification of the anti-parallel lamellar stacking (PMI), slipped-stacked herringbone (DPAPMI), ‘cross mode’ packing with two π···π stacking (CPMI) and anti-parallel herringbone (DMAPMI) packing structure.

**Fig. 5:**
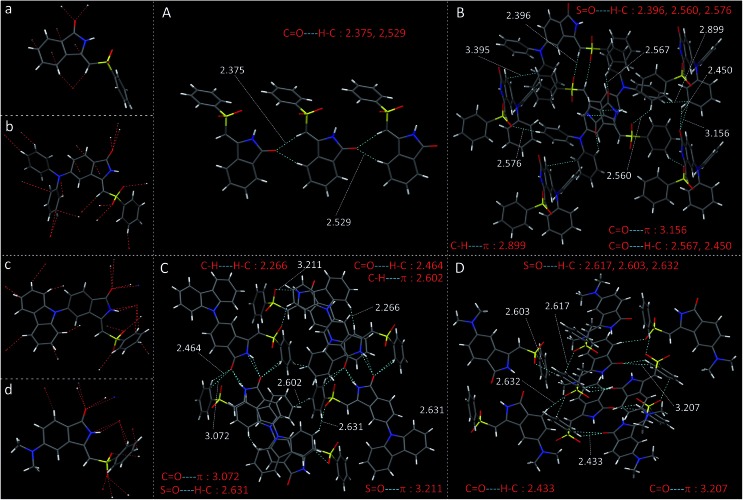
Crystal structure of (a) PMI (b) DPAPMI (c) CPMI and (d) DMAPMI and all kinds of multiple non-covalent interactions in the crystals of (A) PMI (B) DPAPMI (C) CPMI and (D) DMAPMI.

The best way to decipher solid-state optical and mechanochromic properties is through the molecular level understanding of the luminogens by the SCXRD study, which will also provide a clear insight regarding the structure–property relationship. For the SCXRD study, a good quality crystal has been grown from the binary mixture of MeOH : DCM (1 : 1) at 20 °C for all luminogens (crystallographic data are provided in the ESI†). According to our design conjecture, a strong intramolecular H-bonding (2.168 Å) interaction has been detected between N–H and the sulfone group in the parent PMI molecule, which serves to lock the molecule in the *Z*-conformation (Fig. S3(A)†). The rigid *Z*-conformer creates a one-dimensional planar sheet aided by multiple hydrogen bond (C=O···H–C) interactions in the symmetric repetitive fashion (Fig. 5). Such planar sheets are arranged on the top of each other by the strong π···π stacking (3.365 Å) with a head-to-tail arrangement (Fig. 4). Due to the strong π···π stacking and intramolecular H-bond assisted planar structure, the PMI molecule forms lamellar packing (Fig. 3) and shows destructive ‘ACQ’ (dark state) in the solid and aggregated states of the THF/water binary mixture.

The crystal of DPAPMI displays an entirely different packing mode. DPAPMI molecules arrange in a complicated herringbone packing (Fig. 3), in an antiparallel slip stacked manner in two-dimensional (2D) patterns (Fig. 4). In the herringbone packing of DPAPMI, two adjacent stacks ‘roll’ in the opposite direction and other alternate stacks are translated by half of the unit cell length along the stacking direction (Fig. 4). Being a typical D–A molecule (*μ* ∼ 8.35 D obtained from DFT), the acceptor PMI part sits just above the DPA donor unit of the lower DPAPMI molecule (see the space-filling model in Fig. 4). Besides this, the propeller-shaped DPA unit helps to keep the π···π stacking between the adjacent molecules, which is reflected in the enhanced π···π stacking distance (3.505 Å) compared to parent PMI (Fig. 4). It is worth noting that a small dihedral angle of 10.48° (*θ*_1_ in Fig. S3(B)†) between the DPA (D) unit with the PMI (A) core infers a possibility of intramolecular charge transfer (ICT) upon photoexcitation. Interestingly, among two aryl groups in the DPA unit, one moderately distorts at ∼49.17° (*θ*_2_), while the other aryl group highly twists to ∼77.78° (*θ*_3_) with respect to the PMI core to fit into the crystalline lattice (Fig. S3(B)†). Such tilted phenyl blades of the DPA unit probably may help to produce metastable states under external force, which may lead to yield mechanochromism. Another intriguing parameter that holds the herringbone orientation and arrangement is multiple non-covalent interactions such as C=O···H–C (2.56 Å and 2.45 Å), C–H···π (2.89 Å), C–H···O=S (2.39 Å, 2.56 Å, and 2.57 Å), C=O···π (3.15 Å), *etc.* (Fig. 5). Moreover, the stability of the herringbone packing also depends on the strength of the above-mentioned interactions. To provide a clear idea regarding the strength of interactions, we have mapped the Hirshfeld surface (for details see note S2 in the ESI†) taking a neighboring molecule depicted in Fig. S4.† The universal color codes red, white and blue indicate the strong, medium and weak interaction, respectively. Obviously, among these numerous non-covalent interactions, the quantitative prediction of particular interaction(s) in mechanochromism is a highly challenging task. Herein for the first time, we have attempted to study the specific contribution of non-covalent interaction(s) to mechanochromism using (quantitative) Hirshfeld surface analysis discussed in the next section. Notably, most of these interactions are lost upon amorphization under mechanical stress and thus metastable states are generated with distinct energy states and optical properties. To clearly demonstrate the difference in optical properties after mechanical crushing, we briefly highlighted the optical properties of DPAPMI in the crystalline state (Fig. S5†). Emission spectra of the DPAPMI crystal reveal a major peak at ∼570 nm and a peeping peak at ∼430 nm, suggesting the existence of CT and LE states, respectively. Picosecond time resolved decay studies of the crystal (collected at CT peak) exhibit bi-exponential decay (Fig. S5†), with transients of 11.2 ns (48%) and 2.9 ns (52%). Interestingly, the major component shows a shorter lifetime comparable to the dilute (8 μM) solution (THF) state (2.9 ns, collected at the ICT peak), corroborating its monomer like behavior in the crystalline state due to the herringbone arrangement. On the other hand, a longer component could arise due to the excited oligomers resulting from the weak π···π stacking interaction between DPAPMI molecules in a 2D way.

The dimethylamine substituted PMI derivative, *i.e.*, DMAPMI also displays a ‘herringbone’ packing like DPAPMI (Fig. 3). The similarity between DMAPMI and DPAPMI arises because both of them contain flexible acyclic donor moieties. The space-filling model shows a head to tail packing arrangement of individual DMAPMI molecules (Fig. 4). The ‘herringbone’ packing of DMAPMI is stabilized by the several van der Waals and non-covalent interactions (Fig. 5 and S4†). Moreover, DMAPMI exhibits a nearly planar structure owing to a negligible twisting angle of 2.99° between DMA (D) and the PMI (A) core (Fig. S3(B)†), which provides important information on the structure–property relation for mechanochromic molecule design. Moreover, the negligible angle between D and A moieties along with the strong donor ability of DMA group makes DMAPMI the highest feasible molecule for an efficient CT process among all. The high CT efficiency in this molecule is also evident from the higher dipole moment (*μ*) value of ∼9.41 D (obtained from DFT calculations), and the partially double bond character of the C–N bond (*d*_C–N_ = 1.366 Å) between the donor (DMA) and the acceptor (PMI) moiety (Fig. S6†). Cyclization of donor moieties in DPAPMI causes enormous changes in crystal packing. The cyclized analogue of DPAPMI, *i.e.* CPMI forms a rarely observed ‘cross mode’ packing along the long axis of the one-dimensional (1D) column in a symmetrical fashion (Fig. 3). The carbazole moiety in CPMI makes a twisting angle of 43.10° with the PMI core (Fig. S3(B)†), which is slightly higher than that of the isolated molecule (40.75°) in the gas phase (calculated by the DFT study). Owing to the D–A skeleton, the CPMI molecule is packed in such a way that the carbazole of one CPMI molecule comes closer to the central PMI core of another CPMI molecule triggered by the opposite dipole–dipole interaction (Fig. 4). The stability of such a packing mode has been maintained by two weak π···π stacking (3.499 and 3.50 Å) interactions between the carbazole and PMI moiety (Fig. 4). This observation is scarce, as in most of the cases two nearby organic molecules form a single π···π stacking interaction, however, the twisted geometry of CPMI assists the formation of two π···π stacking interactions, which provides extra stability in ‘cross mode’ stacking. The crystal of CPMI also gets rigidified with the aid of numerous non-covalent interactions, such as C–H···O=S (2.631 Å), C=O···H–C (2.464 Å), S=O···π (3.211 Å and 3.072 Å), C–H···H–C (2.266 Å), C–H···π (2.602 Å), *etc.* (Fig. 5 and S4†). The aforementioned ‘cross mode’ packing and non-covalent interactions eliminate molecular vibration, and are responsible for boosting up the solid-state quantum yield (80(±10)%) of the CPMI crystal compared to other derivatives. Notably, to date, numerous strategies have been established for the design of efficient solid-state emissive materials;^49^–^51^ among them ‘cross mode’ stacking has been considered the most preferred one.^52^ The CPMI crystal emits only from the ICT state at ∼510 nm (Fig. S7†), irrespective of the excitation wavelength due to the existence of a single twisted orientation of the donor moiety. Notably, a significant blue shift (60 nm) is observed in the emission maximum of the CPMI crystal compared to its acyclic analogue DPAPMI crystals (Fig. S8†). The blue shift may be attributed to the ‘cross mode’ packing of the CPMI molecule. Moreover, the time-resolved decay of the CPMI crystal reveals a major transient of 7.9 ns (Fig. S7†), corresponding to a monomer like decay as evidenced by the appearance of a similar kind of lifetime component (7.5 ns) observed in dilute (8 μM) THF solution (Fig. S7†).

### Hirshfeld surface analysis and void space calculations

**Fig. 6:**
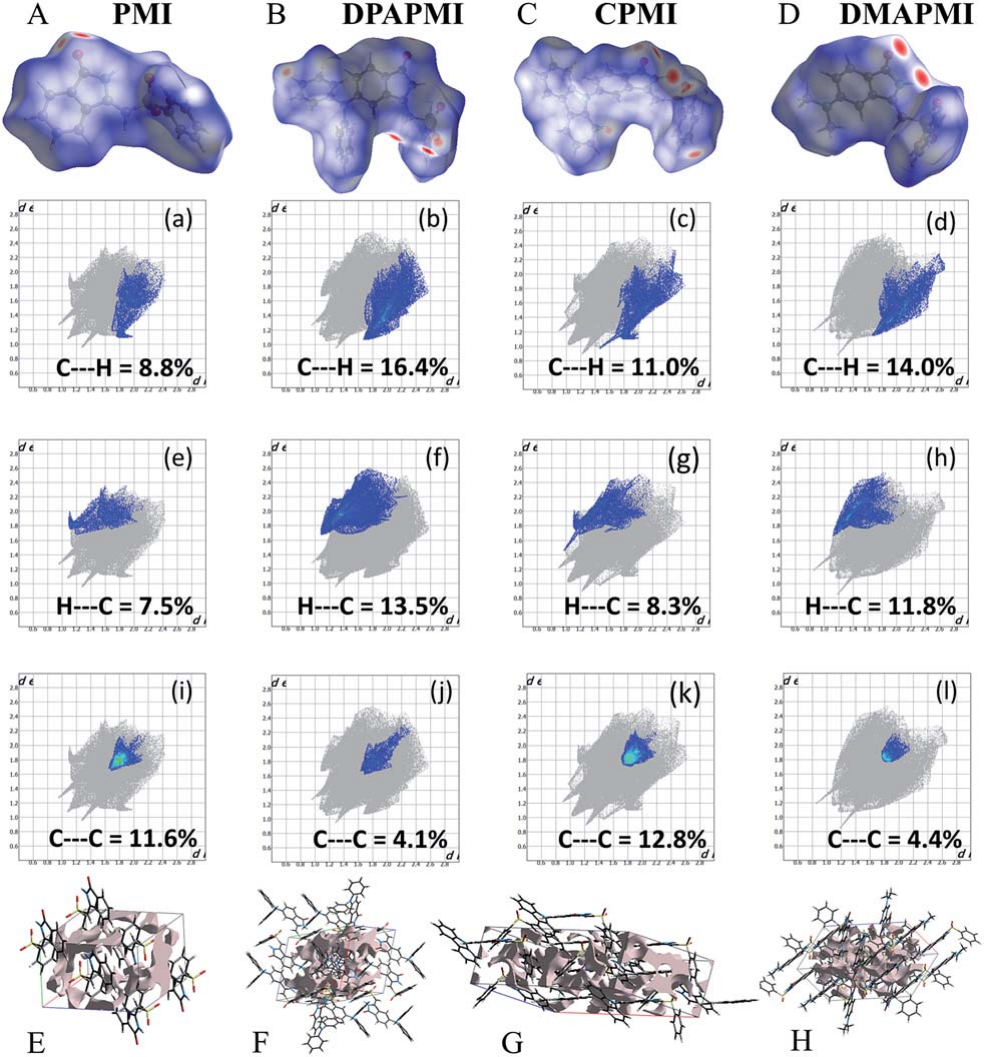
Normalized distance (*d*_norm_) mapped over the Hirshfeld surface of each luminogen (top), and generated fingerprint plots from *d*_norm_ for C···H (middle two rows) and C···C (bottom row) interactions. The grey part in 2D finger plots indicates the total interactions calculated from *d*_norm_. Fig. E to H represent the calculated void space (light pink color region) in PMI, DPAPMI, CPMI and DMAPMI, respectively.

**Fig. 7:**
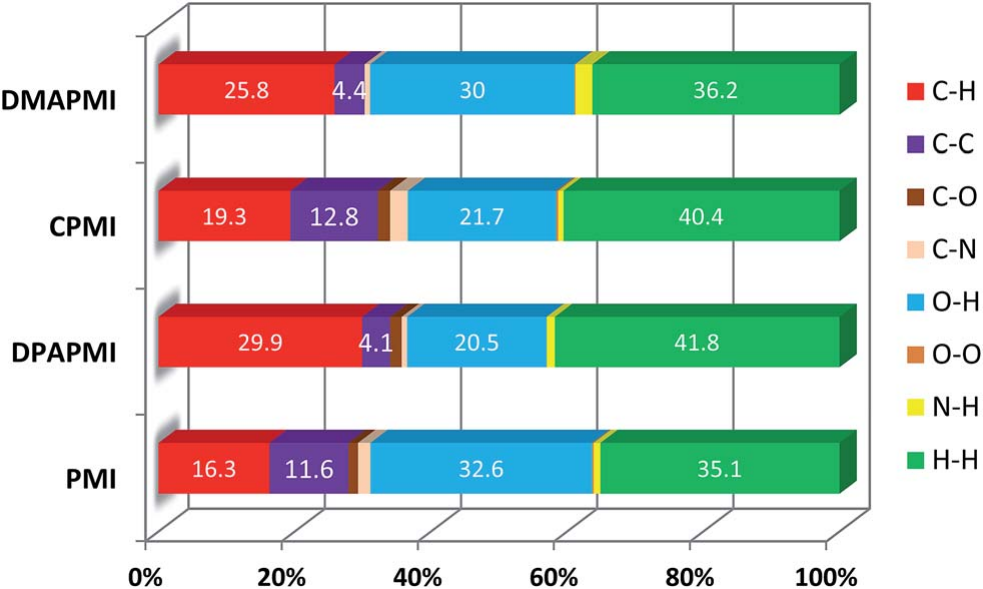
Histogram summarizes the non-covalent interactions (%) obtained from 2D fingerprint plots. Here, H···H indicates van der Waals type interactions.

Hirshfeld surface analysis is particularly useful in studying the effect of donor substitution on the crystal packing and evaluation of the specific contribution of non-covalent interaction(s) to mechanochromism (for details see Note S2 in the ESI†). All possible interactions have been presented in Fig. 6 (C–H···π and π···π) and Fig. S9–S12† (other interactions except C–H···π and π···π) and the calculated percentage of each interaction is summarized in Fig. 7. Although the percentage of van der Waals (H···H) interactions accounts for a significant portion out of total interactions (Fig. 7), their contribution towards the stabilization of the packing motif is quite small, since these interactions contribute a low enthalpy (∼0.4–4 kJ mol^–1^) of stabilization. Interestingly, the C–H···π (% C···H) interaction contributed substantially in DPAPMI and DMAPMI luminogens, while the π···π (% C···C) interaction has a minor contribution for all donor substituted luminogens (Fig. 6 and 7). Considering the high enthalpy of the C–H···π interaction (∼10.3 kJ mol^–1^ (ref. 52)) and its significant contribution in DPAPMI and DMAPMI, it is likely to play a pivotal role in their herringbone packing motif. Notably, the crystal packing mode is determined with the help of parameter *ρ*, which basically depends on the ratio of % C–H···π to % π···π interactions.^53^ Since, mechanochromism is mainly dictated by the molecular packing in the condensed state, we believe that C–H···π and π···π interactions are the most important to design such a novel material which will be further verified by the structure–property relationship discussed in the later part of the manuscript.

The *ρ* values obtained for DPAPMI and DMAPMI are 7.2 and 5.86 respectively, inferring the herringbone packing for both these molecules. However, CPMI exhibits the lowest *ρ* value of ∼1.5 owing to its highest % of π···π (% C···C in Fig. 7) interactions (because of two π···π stacking interactions per pair of CPMI molecules discussed in the previous section). Overall the interchain interactions and structural flexibility of the molecule create a void space inside the crystal (see the void space in Fig. 6), which can play a significant role in mechanochromism during the mechanical treatment. Among all the molecules, DPAPMI has the highest amount of void space of 235 Å^3^, probably because of its propeller-shaped flexible DPA units and numerous non-covalent interactions. However, among all molecules, CPMI exhibits the lowest amount of void space (180 Å^3^) despite the presence of multiple non-covalent interactions and a twisted carbazole ring. This is probably because CPMI contains four molecules per unit cell (Fig. S13†), while all other molecules contain two molecules per unit cell. DMAPMI exhibits a much greater void space (230 Å^3^) than CPMI but nearly the same void space as DPAPMI, as it contains flexible acyclic donor (DMA) substitution, like the DPAPMI molecule.

### Mechanochromic study

Since some of our designed molecules have flexibility and void space, their solid state emission properties should depend on the alteration of molecular arrangements in response to external mechanical treatment (mechanochromism), temperature (thermochromism) and exposure to solvent (vapochromism). The pristine powder of the parent PMI molecule exhibits non-emissive behavior (Fig. S14†). Even after strong grinding with a mortar and pestle, the emission efficiency of the molecule does not change at all. This observation suggests that the planar PMI molecule suffers from the ACQ effect due to the strong π–π stacking interaction, which remains unaffected even under high mechanical force (Fig. S14†).

**Fig. 8:**
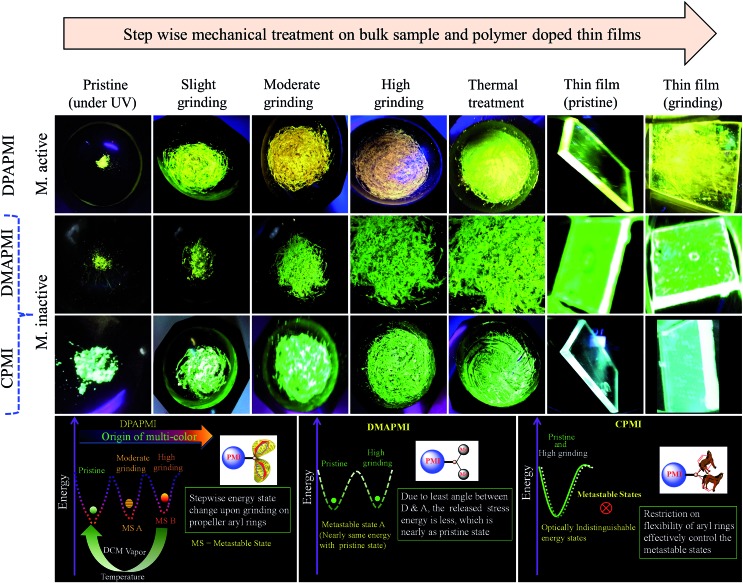
(Top) Luminogens (bulk and thin film state) under different mechanical treatments. DPAPMI shows clear mechanochromism, while CPMI and DMAPMI do not show mechanochromism under any condition. (Bottom) Change of metastable states of luminogens upon mechanical treatment, which leads to multiple colors of DPAPMI. Metastable states drawn based on overall experimental observations.

However, the pristine powder of DPAPMI shows strong emission (*φ*_pristine_ = 65 ± 10%) having an intense peak at ∼545 nm (CT) and a peeping peak at ∼425 nm (LE) (Fig. S15†). Interestingly, the CT peak in the pristine powder shows an ∼25 nm blue shift with respect to the crystalline state (*λ*_em_ ∼ 570 nm), although the peeping peak (LE state) remains nearly unaltered (Fig. S16†). This observation infers that the DPAPMI molecule takes a more planar conformation in the crystalline state, which favors relatively stabilized CT states. Notably, stepwise mechanical grinding of the pristine powder causes a significant modification in the molecular packing, and thereby, modulates the emission features of LE and CT states in the solid state by modulating their energy states (Fig. 8). Upon slight grinding, the emission spectrum shows a single redshifted (∼10 nm compared to the pristine powder) CT peak located at ∼555 nm. Consequently, further grinding leads to a redshifted (∼45 nm compared to pristine) CT peak located at ∼590 nm (Fig. S15†). The gradual color change upon step-wise grinding indicates the progressive compactness of DPAPMI, as it contains a propeller-shaped flexible DPA unit with two different orientations of two aryl rings (at ∼49.17° (*θ*_2_) and ∼77.78° (*θ*_3_)) compared to the PMI core (Fig. S3(B)†). The twisted conformation of the DPA unit contains a high twisting stress in the solid state with a large accessible empty (void) space (235 Å^3^). Hence, grinding results in the release of twisting stress and rupturing of non-covalent interactions, which probably leads to more planarized individual DPAPMI molecules. As a result, the CT state is getting more stabilized due to the increased orbital overlap between the donor and the acceptor; hence, a redshift is observed under high grinding conditions compared to the pristine powder. Taking together the molecular conformation and emission color change upon stepwise grinding of DPAPMI, it is clearly understandable that two metastable states are generated due to the presence of two flexible aryl rings with different twisting angles (see the energy diagram in Fig. 8). Notably, AFM images before and after grinding (Fig. S17 and S18†) ensure that grinding leads to splitting up of the DPAPMI molecular assembly from a larger size to a much smaller size probably because of rupturing of numerous non-covalent interactions with subsequent changes in energy states. Moreover, to decipher a relationship between mechanochromism and the change of molecular arrangement by external stimuli (grinding),^54^ we have employed PXRD measurements. The pristine powder of DPAPMI is found to exhibit several intense and sharp reflection peaks indicating a well-ordered microcrystalline structure (Fig. S19†). After grinding, a broad halo is found with the original signals in relatively lower intensity, indicating a poorly organized semi-crystalline (combination of crystalline and amorphous) state. Changes in the PXRD pattern before and after grinding suggest the modulation of crystallinity in the DPAPMI powder after applying an external mechanical force. Moreover, the broad emission spectrum under grinding conditions also suggests a structural modulation from crystalline to amorphous or *vice versa* (Fig. S15†). In addition, DSC measurements of the ground powder pose two transition peaks at 75 °C and 180 °C prior to melting at 238 °C (Fig. S20†). This clearly indicates that the ground powder is present in two metastable semi-crystalline states. Hence, pristine DPAPMI exhibits a thermodynamically stable crystalline state, while the grinding process changes it into two metastable semi-crystalline states.

The paramount importance of any mechanochromic material depends on its reversible switching ability from the ground state to the initial state, since it enables multiple reusing capabilities of the luminogen. Successively, an instant isothermal reversible color change is monitored by the naked eye by exposing a highly ground powder to DCM vapor (good solvent) (Fig. S21†). However, no color change was observed upon exposure to bad solvent (MeOH, water) vapor. Notably, owing to the huge void space (235 Å^3^) in DPAPMI, the good solvent (DCM) molecule can access inside that accessible void space resulting in the rearrangement of the crystalline state of the luminogen molecules. The PXRD measurements reveal the transformation from the metastable semi-crystalline state to the crystalline state upon DCM treatment (Fig. S21†). Moreover, thermal annealing (at 120 °C for 1 minute) of the ground DPAPMI powder recovers the initial color (Fig. 8). The sharp and intense peaks in PXRD measurements also reveal the recovery of the highly ordered crystalline state (Fig. S19†). Most intriguingly, the emission color of the ground powder of DPAPMI can also recover spontaneously at room temperature within 60 minutes without using any external stimuli like the DCM vapor and temperature. The self-recovered powder exhibits individual emission peak maxima at ∼535 nm (CT peak) and 425 nm (LE peak), which are very close to those of the pristine powder (Fig. S15†). Once it comes back to the initial condition, the optical properties do not change even after one month. This kind of self-reversibility is extremely rare and to the best of our knowledge until now only one report exists on spontaneous recovery based on the diphenyl benzofulvene derivative.^55^

In modern technological applications, mechanochromic luminogens are mostly used as thin films, where they often stay as thin layers or in segregated states.^9^,^56^ To check whether mechanochromic behavior is retained in the segregated state or not, a PMMA (polymethyl methacrylate, 20 wt%) polymer doped DPAPMI thin film has been prepared (for fabrication see the Experimental section in the ESI†), where PMMA acts as a segregating agent. The emission spectrum of the DPAPMI thin film consists of a dominating emission peak at ∼535 nm corresponding to the CT state (Fig. S15†). Grinding of the thin film with a spatula shows mechanochromism with a similar color change to pristine (Fig. 8). Interestingly, DCM vapor and thermal treatments effectively reverse back the fluorescence properties of the thin film (data not shown), suggesting that all properties seen in the bulk state are also retained in this segregated state.

The pristine powder of CPMI exhibits a single unstructured emission band centered at ∼510 nm corresponding to the CT state (Fig. S15†). Surprisingly, the crystal of CPMI also emits nearly at the same position (∼508 nm) (Fig. S15†). Thus, the above observation infers that the molecular packing modes in the pristine powder and crystalline form are nearly alike. Moreover, a similar type of solid-state UV-Vis absorption, and pico-second lifetime decay with nearly the same component of the pristine powder (5.27 ns) and the crystal (5.12 ns), (Fig. S22†) further suggest a similar structural arrangement in the powder and the crystal. Considering all these observations, we believe that CPMI maintains a highly stable ‘cross mode’ packing in the pristine powder likewise the crystal. To our surprise, upon mechanical grinding (with a mortar and pestle), the color of the CPMI pristine powder does not change at all (Fig. 8). Even after vigorous grinding with ball milling (1000 rpm, 10 minutes), emission spectra do not show any change in their shapes and peak positions. In addition, we have also attempted to study thermo-responsive mechanochromism by heating the sample at 180 °C (below the melting point) along with constant grinding with a mortar and pestle. However, we did not observe any thermo-responsive mechanochromic behavior of CPMI (Fig. 8). Moreover, we have also checked the emission feature of the pristine powder after soaking in DCM (good solvent) vapor, showing an unaltered emission feature. It must be mentioned here that CPMI contains the lowest amount of available void space (180 Å^3^), which rarely allows the molecule to take different metastable energy states under mechanical stress and external stimuli. Thus, we conclude that it is not possible to disturb the architecture of highly stable ‘cross mode’ molecular arrangements of CPMI with the aid of any external stimulus and stress, and hence, CPMI is considered as a mechano-inactive molecule. It is also clear from PXRD data that the crystalline feature of CPMI before and after the grinding almost remains intact (Fig. S19†). Additionally, no metastable state in DSC measurements is found in the case of the ground powder of CPMI (Fig. S20†), which also explains the mechano-inactive behavior of CPMI. Notably, this kind of mechano-inactive molecule, like CPMI, exhibiting a ‘cross’ molecular packing may be applicable for the fabrication of optical light emitting devices (OLEDs) owing to its high quantum yield (80(±10)%) and suitable band gap with appropriate push–pull features.

The pristine powder of DMAPMI exhibits a single unstructured emission band centered at ∼535 nm corresponding to the CT state only (Fig. S15†). Despite flexible acyclic donor substitution in DMAPMI, it does not contain any LE peak like DPAPMI, probably because of it's nearly planar conformation (2.99°), which facilitates efficient charge transfer from DMA to PMI core. Astonishingly, upon high grinding of the pristine powder of DMAPMI with a mortar and pestle, emission spectra remain nearly unaltered (Fig. S15†). Like for CPMI, we have checked the emission spectra after treatment with solvent vapor (acetone and DCM) and grinding under thermal treatment (180 °C) for DMAPMI, which also shows an unaltered emission feature (Fig. 8). PXRD measurements of the pristine powder of DMAPMI show a semi-crystalline (combination of crystalline and amorphous) state, however, it loses its crystallinity completely under grinding (Fig. S19†). Moreover, DSC measurements of the ground powder reveal a transition state at 62 °C before its melting at 270 °C, suggesting the presence of a single metastable state (Fig. S20†). Collectively, PXRD and DSC results suggest that the pristine powder exhibits a thermodynamically stable semi-crystalline state, while mechanical grinding changes it into the metastable amorphous state. Surprisingly, despite the existence of the metastable state, the emission maximum does not change at all, which clearly indicates that grinding induced metastable states are energetically nearly the same as those of the pristine powder (see the energy diagram in Fig. 8). This is probably because DMAPMI does not contain any twisting stress, due to its negligible angle (2.99°) between DMA (D) and the PMI (A) core.

### Outline from the mechanochromic and crystal study and structure–property relationship

Based on the mechanochromic behavior and accurate molecular level understanding of each luminogen, we have tried to build a structure–property relationship to design a mechanoactive luminogen. Considering all observations, we noticed that three factors namely the twisting and flexibility of the donor moiety and non-covalent interactions (mainly C–H···π and π···π interactions) play major roles in mechanochromism. The importance of the twisting of the donor moiety in mechanochromism can be understood by looking at the molecular structure, packing style and Hirshfeld surface analysis of DPAPMI and DMAPMI. Hirshfeld surface analysis reveals nearly the same % C–H···π and % π···π interactions for DPAPMI (C–H···π 29.9%, π···π 4.1%) and DMAPMI (C–H···π 25.8%, π···π 4.4%) (Fig. 7). Moreover, both luminogens exhibit the herringbone packing in the solid state along with nearly the same void space of 235 Å^3^ and 230 Å^3^ for DPAPMI and DMAPMI respectively. Hence, DMAPMI is expected to show mechanochromic behavior considering a similar kind of packing, void space and nearly the same % of non-covalent interactions likewise DPAPMI. However, mechanochromic studies reveal that DPAPMI is mechanoactive, while DMAPMI is a mechano-inactive luminogen. Thus, it is clear that twisting (of donor) plays a significant role in designing a mechanochromic material, since the twisting angle of the donor is the only structural difference between them (see Fig. S3(B)†). Secondly, the role of the flexibility of the donor in mechanochromism can be perceived by comparing the void space of DPAPMI and its cyclic analogue CPMI. CPMI has the least void space (187.4 Å^3^) owing to its cyclized donor unit and at the same time, it is also a mechano-inactive molecule. Thus, the mechano-inactivity of CPMI compared to its acyclic analogue DPAPMI suggests that a flexible donor unit is desirable to construct a mechanochromic material. Thirdly, Hirshfeld surface analysis of mechanochromic DPAPMI suggests that both C–H···π and π···π interactions are important, however, it seems that the C–H···π interaction is dominant over the π···π interaction to yield mechanochromism. To provide further insight, we have extensively mapped the Hirshfeld surface over the shape index and curvedness to get specifically the π···π interaction region. Interestingly we have noticed that the mechanoactive DPAPMI molecule contains the π···π interaction region in a small part residing at the central aryl ring with a minimum percentage of 4.1%, while other luminogens exhibit the π···π interaction in a much wider region with higher percentage (Fig. S23 and S24†). Higher π···π interactions enhance the possibility of planarity and exciton–phonon coupling in the luminogen^29^,^57^,^58^ which will subsequently reduce the possibility of mechanochromism and emission contrast respectively. Hence, mechanochromic luminogens should be engineered in such a way that the π···π interaction must be there to a minimum extent. To achieve such a type of luminogen, attachment of a flexible and twisted donor unit in the CT type luminogen is strongly recommended as such a conformation can minimize the π···π stacking interaction, likewise the DPAPMI luminogen. It must be noted that alone none of this parameter can produce a mechanochromic material. Hence, to design a mechanochromic material not only flexibility but also twisting of the donor unit along with multiple non-covalent interactions (especially major C–H···π and minor π···π interactions) is necessary in aromatic CT luminogens. Notably, as co-operatively twisting, flexibility and multiple non-covalent interactions (C–H···π is preferable over π···π interactions) control the metastable energy states under external stimuli, we believe that this strategy may also be beneficial as a general strategy to design multi-stimuli responsive luminogens.

### Solvatochromic study

To unveil the distinct optical properties in the solution state of donor substituted PMI derivatives, we have performed fluorescence studies in various solvents having different polarity and the results are summarized in Fig. S25–S28 and Table S1† (absorption spectra are given in Fig. S26†). The emission profiles of donor substituted PMI derivatives seem to be dictated by the flip-flop motion and charge transfer character induced by the donor moieties. DPAPMI exhibits two emission peaks at ∼400 nm and 485 nm in benzene (Δ*f* = 0.003) upon excitation at 350 nm (Fig. S25†). The high energy peak corresponds to the emission from the Frank–Condon or LE state (as observed in the case of the parent PMI molecule depicted in Fig. S27†); whereas the lower energy peak probably originates from a CT state arising from the charge transfer from –N(Ph)_2_ (D) to the PMI core (A). Here, It is pertinent to mention that unlike other conventional ICT/TICT molecules, where the CT state is generated from the LE state, here the CT state can be generated by direct excitation at ≥405 nm (Fig. S28†), and hence the CT state is not a dark state. Thus, we anticipate that with high energy excitation (*λ*_ex_ = 350 nm), the molecule probably reaches a high energy state, in which the diphenyl rings are not properly oriented to have an efficient charge transfer process. Now, the excited molecule can come back to the ground state by a radiative transition at 400–430 nm from that higher energy state. Alternatively, it can also reach the other stabilized state having a CT character. When the molecule emits from this stabilized CT state, it shows a red-shifted emission (CT) compared to the LE state. The formation of the CT character has also been justified by a redshift and dramatic decrease in the emission intensity of the CT peak in highly polar solvents. When the solvent polarity is further increased (Δ*f* > 0.22), the emission profile of DPAPMI consists of only a redshifted LE peak (Fig. S25, Table S1†). This is because of the very weak emission intensity of the CT state in highly polar solvents, which is also evident from the observed weak emission profile collected at selective excitation of the CT state (see the weakly emissive spectra of DPAPMI in Fig. S28†).

When two phenyl moieties are replaced by methyl groups, then the solvatochromic behavior dramatically changes. The methyl substituted derivative DMAPMI exhibits a low intensity peak at ∼430 nm and 470 nm in non-polar solvents, like *n*-heptane and benzene, respectively (Fig. S25†). Although spectral features remain the same (*i.e.*, it consists of a lower energy peak and a shoulder on the higher energy side), the lower energy peak shows a gradual red shift with increment of solvent polarity (Δ*f* > 0.014). In highly polar solvents (Δ*f* > 0.31), the lower energy peak exhibits a usual redshift and higher energy emission appears as a shoulder. The high energy peak in *n*-heptane may be attributed to the pyramidal conformation of the –N(CH_3_)_2_ group, which consists of a less charge character, as the –N(CH_3_)_2_ group is out of resonance with the PMI moiety. This claim is further supported by the absorption spectrum, where the absorption for the CT peak (>400 nm) is very much less. The lower energy peak is attributed to the CT emission and this CT nature is further verified by the emission profile collected upon selective excitation of the charge transfer band, *i.e.*, at 405 nm (Fig. S28†). The existence of dual emission (shoulder and lower energy peaks) nature in moderately to highly polar solvents can be rationalized in terms of the population of pyramidal conformation (non-planar) as well as planar structure having a CT character. Although CT is the most stable state in these solvents, flipping motion of methyl groups cannot be avoided, which may lead to some population of pyramidal conformation of DMAPMI. The drastic reduction of CT intensity in higher polarity solvents indicates that there is a new non-radiative deexcitation channel operating for this molecule in these solvents. We anticipate that the new deexcitation channel arises because of the involvement of the non-fluorescent TICT states, where both –N(CH_3_)_2_ and PMI stay in a perpendicular geometry, resulting in maximum charge transfer between donor and acceptor moieties.

The cyclized derivative of diphenyl, *i.e.*, CPMI also exhibits unique solvatochromic behavior. CPMI shows an emission maximum at ∼430 nm upon photo-excitation at 350 nm in non-polar solvent *n*-heptane (Fig. S25†). This high energy peak corresponds to the emission from the LE or Frank–Condon state of CPMI. Unlike diphenyl, here the CT peak is not found in this non-polar solvent probably due to the presence of the cyclized donor group. The cyclized donor restricts the existence of multiple conformers of the molecule, and hence, it exhibits one stabilized LUMO corresponding to that particular conformer. This is also evident from the crystal structure, where the carbazole moiety makes an angle of 43.1° with respect to the PMI core. With the increase of the solvent polarity (Δ*f* = 0.02 to 0.2), dual emission peaks appear in the emission profile, where the CT peak dominates the LE peak suggesting that LE and CT states are proximately closer in energy in this polarity region (Fig. S25†). Moreover, the longer wavelength emission (exhibiting spectral shifts) shows an obvious charge transfer (CT) characteristic whose transition dipole moment is affected by the solvent polarity (Fig. S28†). Drastic retardation of the emission intensity of the CT state in highly polar solvents is attributed to the formation of a highly stabilized CT state, which may lead to the formation of a non-fluorescent TICT state.

### Applications

The reversible mechanochromic behavior of DPAPMI may put forward this molecule as a potential candidate for application in optical storage media, deformation sensors, volatile organic compound (VOC) sensors and security ink. Moreover, highly emissive CPMI may be useful as a possible candidate for organic lasing materials and OLED fabrication. Here, we have demonstrated the applications of these newly developed luminogens in fluorescence thermometers, lighting up cells, rewritable media and acid–base induced fluorescence switching media.

### Fluorescence thermometers

**Fig. 9:**
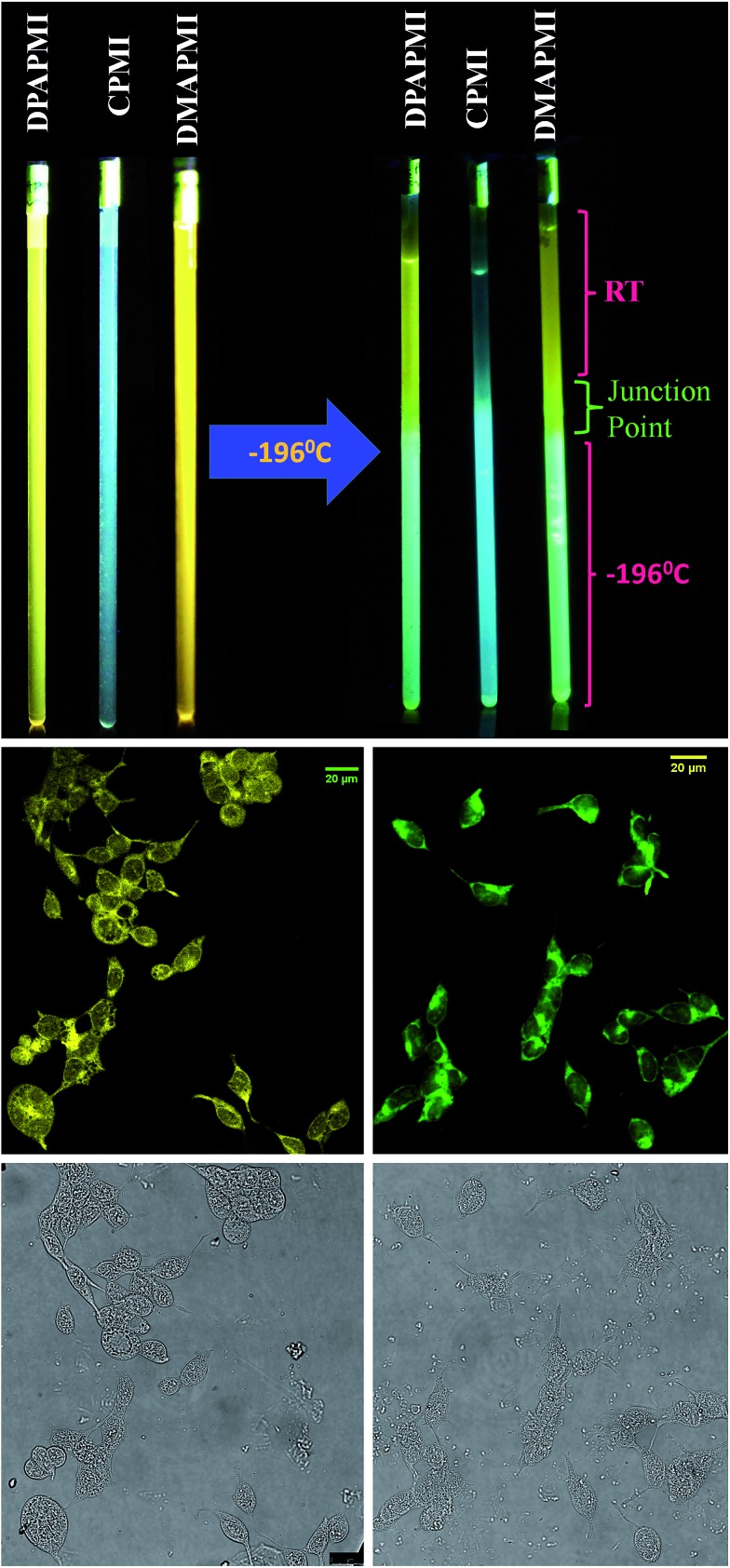
(Top) Fluorescence color switching with temperature. (Middle) Confocal images of HEK 293 cells labelled with DPAPMI (left) and CPMI (right). Bright field images are provided in the bottom row.

Charge transfer (CT) luminogens having fluorescence switching ability between LE and ICT states by varying temperature are ideal for the construction of fluorescence thermometers, which are often used in industrial, atmospheric and deep-sea research, where conventional thermometers can't be used.^59^ To check the temperature dependent fluorescence switching, we have measured the emission spectra of each donor substituted luminogen in a glass forming alcoholic mixture (4 : 1 methanol/ethanol) in a wide range of temperatures. Notably, when DPAPMI was brought from room temperature to 77 K, it switches the fluorescence from the pure CT state to a mixture of LE and ICT states, where the LE peak dominates the ICT state (Fig. S29†). Similarly, DMAPMI undergoes a switching from CT state to LE state conversion upon attenuation of temperature from 25 °C to –196 °C (Fig. S29†). At room or higher temperature efficient solvation stabilizes the CT state, while at lower temperature due to hampered solvation around the probe, the LE state is stabilized compared to the CT state. However, CPMI does not exhibit any fluorescence switching, and it only shows a typical intensity enhancement with lowering the temperature (Fig. S29†). To demonstrate this fluorescence switching in thermometer construction, we have applied a temperature gradient by taking each luminogen solution into quartz tubes (see Fig. 9 and ESI Movie†). Each luminogen filled quartz tube was cooled at its lower half portion by dipping into liquid nitrogen (–196 °C). Interestingly, the observed color change pattern nicely corroborates the measured emission spectra.

### Lighting up cells

Luminogens with aggregation-induced emission (AIE) have been recognized as potential candidates in recent years to light up a targeted part of the cell.^60^ Considering the AIE properties of DPAPMI and CPMI, we have stained both luminogens in human embryonic kidney (HEK) 293 cells to check their lighting up ability (see the Experimental section in the ESI† for the cell culture and fluorophore labeling part). From the confocal images, it is clear that the yellow and green luminescent DPAPMI and CPMI molecules are highly cell penetrable and they are very useful to light up the cell, predominantly the cytoplasm part (Fig. 9).

### Rewritable media and acid–base induced fluorescence switching

**Fig. 10:**
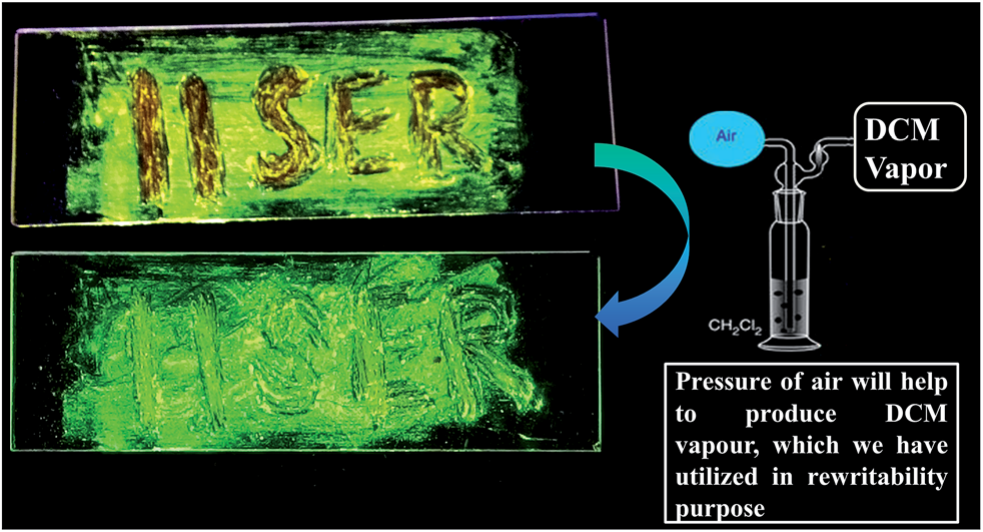
Demonstration of a rewritable data recording device based on mechanochromic and vapochromic luminescence of the DPAPMI luminogen. (Top) Color change upon grinding with a metal spatula. (Bottom) Color switching upon DCM vapor exposure (glassware used for the DCM vapor experiment is shown on the right side briefly). Both images were taken under the irradiation of 365 nm UV light.

To demonstrate rewritable media application, we have chosen DPAPMI, as this molecule only shows mechanoactivity. For this purpose, ‘IISERP’ has been written with a metal spatula on a glass substrate and the grinding induced emission color was monitored under UV irradiation (Fig. 10). The written word can be erased upon thermal treatment or DCM fuming, which demonstrates a potential application in a reproducible recording–erasing process (rewritable media).

Finally, dynamic fluorescence on–off switching of DPAPMI and CPMI has been monitored under TFA and NH_3_ vapor (Fig. S30†). Owing to the strong proton releasing capability of TFA, it blocks the electron flow from the donor to the acceptor moiety by protonation of the electron rich donor part of the molecule, and hence fluorescence is turned off. However, in the presence of NH_3_ exposure, the fluorescence turn-on may be attributed to the formation of a poorly stable conjugate base (CF_3_COO^–^NH_4_^+^).

## Conclusions

This article provides a new avenue regarding the structure–property relationship in order to design mechanochromic materials based on CT luminogens. To achieve our goal, we have developed a series of new isoindolinone based D–A dyes using a one pot synthetic strategy through a Ru metal catalyzed C–H bond activation approach. Slight tuning of the donor moiety is found to be very effective in controlling the molecular packing, and thus, the solid-state optical properties and mechanochromism as well. The crystal-induced emission enhancement (CIEE) effect is believed to be responsible for the observed high quantum yields of two of our synthesized luminogens, namely, DPAPMI and CPMI in the solid state. In DPAPMI (consisting of a diphenylamine group as a donor), the herringbone packing along with multiple non-covalent interactions and the flexible donor unit affords a loose herringbone molecular packing, enabling it to undergo reversible transformation under multiple stimuli. Cyclization of the donor unit (*i.e.* CPMI) leads to a completely different packing mode (cross mode), which subsequently gives rise to the mechano-inactive properties of the luminogen. Surprisingly, although DMAPMI (having a dimethylamine group as a donor) exhibits a comparable packing style and non-covalent interactions to DPAPMI, it does not show mechanoactivity like its close analogue. Besides, Hirshfeld surface analysis specifically infers that non-covalent (C–H···π and π···π) interactions are also responsible for the observed mechanochromic properties. Considering all these aspects, we conclude that the loose molecular packing, conformational twisting and flexibility of the donor along with numerous non-covalent interactions (especially C–H···π over π···π) are crucial for designing efficient CT mechanochromic luminogens. Moreover, these results establish the unique role of the flexible propeller-shaped donor unit in the self-reversible mechanochromism of CT luminogens. Although, we have chosen CT luminogens to establish the structure–property relationship for designing multi-stimuli responsive materials, we believe that this strategy may also be beneficial to use as a general strategy to obtain mechanochromism. Finally, the designed molecules are found to be potentially applicable for fluorescence thermometer construction, lighting up cells (HEK 293), rewritable devices, acid–base induced fluorescence switching, *etc.*

## Conflicts of interest

There are no conflicts to declare.

## Supplementary Material

Supplementary information

Supplementary movie

Crystal structure data
