# Supplementary material for: Developing the structure–property relationship to design solid state multi-stimuli responsive materials and their potential applications in different fields
Source: Chem Sci. 2018 Mar 5;9(14):3592–606. doi: 10.1039/c8sc00143j (PMC5935060; doi:10.1039/c8sc00143j)
Supplement: Supplementary file 1 [file SC-009-C8SC00143J-s001.pdf]

Electronic Supplementary Material (ESI) for Chemical Science.
This journal is © The Royal Society of Chemistry 2018

Electronic Supplementary Information (ESI)

Developing the Structure-Property Relationship to Design Solid State Multi-stimuli Responsive Materials and their Potential Applications in Different Fields

Bibhisan Roy,[a] Mallu Chenna Reddy[a] and Partha Hazra*[a#]

[a] Department of Chemistry, Indian Institute of Science Education and Research (IISER), Pune (411008), Maharashtra, India

#Centre for Energy Science, Indian Institute of Science Education and Research (IISER), Pune (411008), Maharashtra, India

* Corresponding author. E-mail: p.hazra@iiserpune.ac.in. Tel.: +91-20-2590-8077; Fax: +91-20-2589 9790.

Table of Contents

Contents	Page Number
General remarks (Instrumentation)	S3-S4
Materials	S4
Experimental Section	S4-S6
Electron Flow and Proposed Mechanism	S7-S8
Synthesis procedure	S9-S10
Characterization data	S11-S14
Characterization data (figures)	S15-S29
Cartesian co-ordinates of luminogen (DFT study)	S30-S37
Optical properties in THF and Aggregation-induced emission	S38-S39
Crystallographic data Table	S40-S45
Intramolecular H-bond and angle demonstration	S46-S48
Hirshfeld surface mapping (interaction demonstration)	S49-S51
Optical properties in crystalline forms and D-A distances	S52-S55, S62, S63, S69
2D fingerprint plots of Hirshfeld surface analysis	S56-S59
Unit cell of crystal	S60
Mechanochromic study (AFM, DSC, DCM induced color change, PXRD, Emission upon external stimuli)	S61-S68
Shape index and curvedness (π–π stacking region)	S70-S72
Solvatochromism and Applications in different fields	S73-S80

General Remarks (Instrumentation):

The NMR characterization of synthesized compounds were recorded on a JEOL ECS-400 and Bruker Ascend TM 400 spectrometer (^{1}H: 400 MHz; ^{13}C: 100 MHz) using deuterated chloroform ($CDCl_3$) solution with residual chloroform. Chemical shift in ^{1}H NMR (400 MHz) spectra has been measured relative to $CDCl_3$ as the internal standard (δ 7.26 ppm of ^{1}H NMR) and for ^{13}C NMR (100 MHz) it was obtained using $CDCl_3$ as internal standard (δ 77.16 ppm). The characteristic high-resolution mass spectra (HRMS) were obtained on Water's SYNAPT G2 mass spectrometer. The solid state and solution state infrared (IR) spectra were measured on the 'BRUKAR ALPHA' FT-IR Spectrometer (model number 1004790). The melting point of each compound was determined by the VEEGO (model: VMP-D) instrument. The differential scanning calorimetry (DSC) measurements were performed using TA Q20 DSC instrument. The experiment was recorded at the heating and cooling rate of 10 °C/min. During data analysis, first heating cycle was discarded since they possessed the prehistory of the sample. The powder X-ray diffraction (PXRD) measurement was performed by BRUKAR D8 advance X-ray diffractometer with CuKα radiation (λ = 1.5418 Å). The single crystal X-ray diffraction (SCXRD) measurements were performed on the Bruker KAPPA APEX II CCD diffractometer with Mo-Kα radiation source. The dynamic light scattering (DLS) experiment was performed using a Nano ZS-90 apparatus utilizing 633 nm red laser (at 90° angle) from Malvern Instruments. The Field Emission Scanning Microscopy (FESEM) images were recorded using the ZEISS instrument by drop casting the sample (THF/water) on copper grids. The Atomic Force Microscopy (AFM) imaging was performed on the Key Sight 5500 AFM instrument (Agilent Technologies) under tapping mode with silicon nitride tip. The confocal microscope images were taken using LSM confocal microscope. The steady state absorption and emission spectra were recorded using the UV-Vis spectrophotometer (Shimadzu, UV-2600) and Fluoromax-4 spectrofluorimeter (Horiba Jobin Yvon) respectively. Emission lifetime decays were collected by time-correlated single photon counting (TCSPC) set-up from Horiba Jobin Yvon, using 375 nm diode laser (IBH, UK, NanoLED-375L). Fluorescence signals were collected at the magic angle using MCP-PMT

(Hamamatsu, Japan) detector. The absolute quantum yield of powder and crystal were obtained on the HAMAMATSU C11347-01 spectrometer using an integrating sphere model.

Materials

For synthesis, we have used commercially available starting materials purchased from Sigma Aldrich. Most of the solvents used for the synthesis purpose were purchased from Sigma Aldrich and were degassed by the three freeze-pump-thaw cycles followed by dried over the molecular sieves (4 Å) before using them in metal-catalyzed reaction. For spectroscopic measurements, spectroscopic grade solvents (Spectrochem Pvt. Ltd. India) have been used. Few important chemicals, such as [{$RuCl_2$ (*p*-cymene)}$_2$] (CAS No. 52462-29-0), $AgSbF_6$, (CAS No. 26042-64-8), $Cu(OAc)_2 \cdot H_2O$, phenyl vinyl sulfone (PVS) (CAS No. 5535-48-8), benzonitrile**,** 4-fluorobenzonitrile, polymethyl methacrylate (PMMA) were purchased from Sigma Aldrich and were used as received. The substituted starting materials such as 4-(diphenyl amino) benzonitrile (2b) and 4-(9H-carbazol-9-yl) benzonitrile (2c) were synthesized by the reported procedure.[1,2] The starting materials 4-(Dimethylamino) benzonitrile (2d) (CAS No. 1197-19-9) was purchased from Sigma-Aldrich.

Experimental Section

Density Functional Theory (DFT) Calculation:

The quantum mechanical calculations were conducted using Gaussian 09' program (revision D.01) suite using a High-Performance Computing Cluster facility of IISER PUNE. All the calculations carried out by density functional theory (DFT) with Becke's three-parameter hybrid exchange functional and the Lee-Yang-Parr correlation functional (B3LYP) and 6-31G (d,p) basis set. Each luminogen was optimized in the gas phase, and the nature of stationary point (in these case minima on the potential energy surface) was confirmed by the normal-mode analysis. Molecular orbital contributions were determined using Gauss Sum 2.2.program package. All the coordinates for each luminogens are provided in this ESI.

Fabrication of Nano-Aggregates in THF/Water Binary Mixture (for AIE Study)

Nanoaggregates of each molecule were fabricated by the simple precipitation method without using any surfactant. For different fraction of water, distilled water was slowly added in THF solution containing luminogen (concentration 8 μM), under vigorous stirring. Fabricated nanoaggregates were characterized by different techniques, such as AFM, DLS and FESEM.

Fabrication of Polymer (PMMA) Coated Thin Film

Polymer coated thin films were fabricated on 20 × 20 mm quartz slides by spin coating method. For this purpose, a solution of 20% PMMA polymer in THF solution containing luminogen concentration of 8×10^{-4} (M) were used (~10 drops) on the quartz slide under constant rotation at 3000 rpm. Fabricated films were properly dried in vacuo before taking spectroscopic and mechanochromic measurements.

Cell culture Procedure

Human embryonic kidney (HEK) 293 cells were maintained in Dulbecco's Modified Eagle's medium (DMEM, a high glucose) supplemented with 10% Fetal Bovine Serum, penicillin (100 units/ml), and streptomycin (100μg/ml) and maintained in a humidified condition containing 5% CO_2 at 37^oC.

Luminogens (CPMI and DPAPMI) Labelling

2 ml of HEK 293 cells (0.5×10^6/ml) were seeded in 6 well plates and allowed to attach overnight on to sterile glass coverslips. The CPMI and DPAPMI (this two luminogens are AIE active, and, hence they are selected for lighting up cells) luminogens stock (160 μM) were prepared in phosphate buffered saline (PBS) containing 4.2% DMSO, and 100 μl of this stock was used for each experiment to attain a final concentration of 8 μM . Cells were incubated with corresponding luminogens for 24 hours at cell culture conditions mentioned above. After incubation, cells were washed once with PBS, followed by fixation by methanol: acetic acid (3:1) for 1 hour at 4^oC. These coverslips were washed again with PBS for three times and fixed on to glass slides with 70% glycerol as mounting medium, and the cells were imaged using the confocal microscope by exciting at 405 nm (blue channel) laser. All the images were analyzed using ImageJ analysis software.

Dynamic Fluorescence Switching under Acid-Base Vapor

To demonstrate fluorescence switching under acid-base vapor, we have chosen DPAPMI and CPMI luminogens as both of them are highly emissive in solid state due to AIE property. For this application, at first two filter paper (for two luminogen) written with 'IISERP' (using THF solution of both luminogen) dried properly. Both filter papers were found to be highly emissive at 365 nm UV light exposure (Figure S30). After that, both filter papers were kept under TFA (strong acid) exposure for a while (less than 1 minute) and the emission was found to be turned-off immediately (Figure S30). Interestingly, the fluorescence emission can be recovered by exposing NH_3 vapor (base) for nearly 1 minute, demonstrating a clear reversible fluorescence switching ability of luminogen under acid-base vapor. Owing to strong proton releasing capability of TFA, it may block the electron flow from donor to acceptor moiety by protonation of electron rich donor part of the molecule, and hence fluorescence will turned off. However, fluorescence turn on in presence of NH_3 exposure may be due to the formation of poorly stable conjugate base ($CF_3COO^-NH_4^+$).

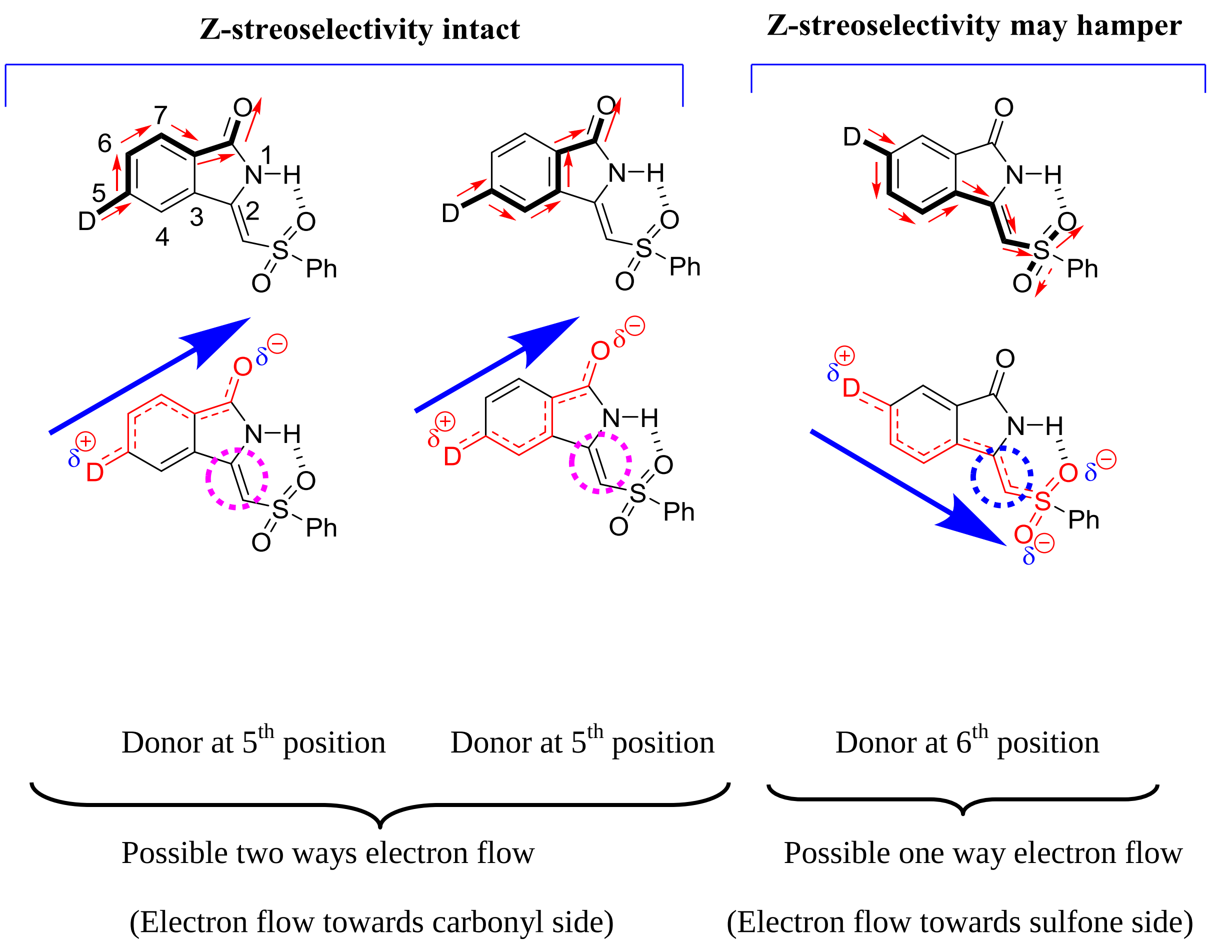

Scheme S1: Representation of electron flow direction with change of donor substitution position. Donor at 5th and 6th position leads to the electron flow towards carbonyl side (left and middle) and sulfone side (right) respectively. The one headed arrow (red color) represents the direction of electron flow.

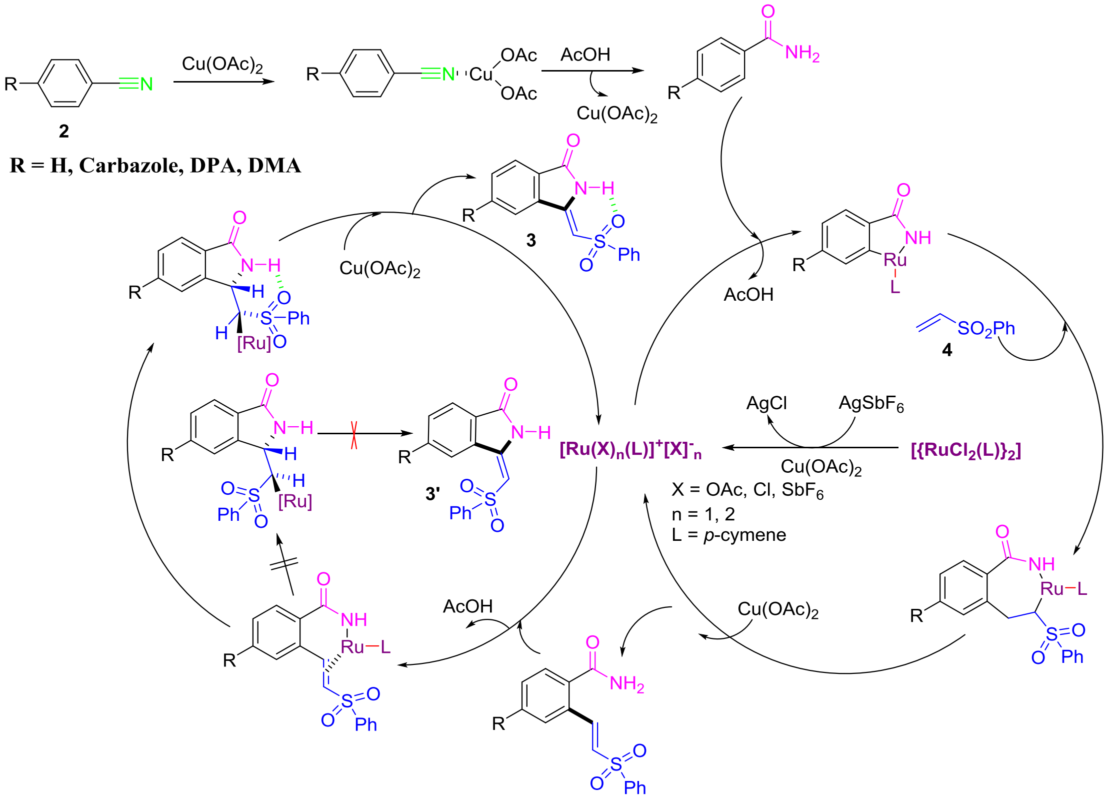

Scheme S2: Proposed mechanism of isoindolinone based CT luminogen formation via Ru metal catalyzed C-H bond activation pathway.

Synthetic Procedure

General Procedure for Synthesis of 2b-2c

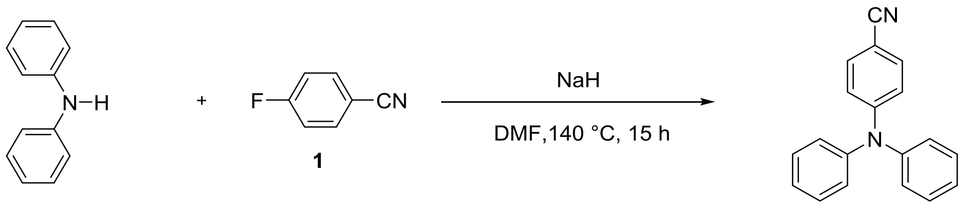

1b 2b

- **Synthesis of 2b**

5.0 g (29.55 mmol) of diphenylamine (1b), 3.936 g (32.505 mmol) of 4-fluorobenzonitrile (1) and 1.24 g (32.505 mmol) of sodium hydride (60 % dispersion in mineral oil) were added to a one-neck round bottom flask and dissolved in 100 mL of DMF. Then, the reaction mixture was allowed to stir at 140 °C for 15 h. After 15 h, the reaction mixture was poured into to ice-cooled water (700 mL) with stirring. Precipitate was filtered and washed with water. Precipitated product was further purified by column chromatography using hexane: ethyl acetate (9:1) as eluent to yield 4-(diphenyl amino) benzonitrile 2b as white solid (5.11 g, 64%).

- **Synthesis of 2c**

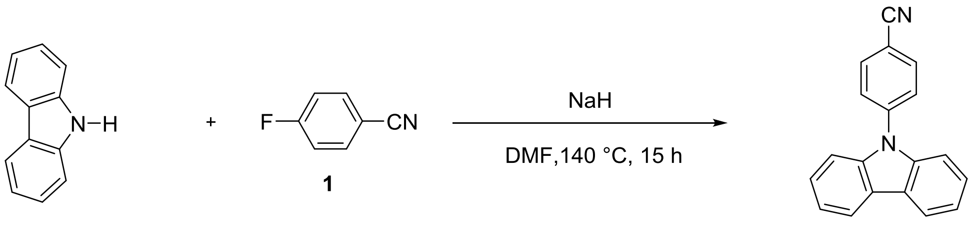

1c 2c

5.0 g (29.90 mmol) of Carbazole (1c), 3.98 g (32.89 mmol) of 4-fluorobenzonitrile (1) and 1.31 g (20 mmol) of sodium hydride (60 % dispersion in mineral oil) were added to a one-necked round bottom flask and dissolved in 100 mL of DMF. After 15 h of reaction, the reaction mixture was poured into to ice-cooled water (700 mL) with stirring. Precipitate was filtered and washed with water. Precipitated product was further purified by column chromatography using hexane: ethyl acetate (9:1) as eluent to yield 4-(9*H*-carbazol-9-yl) benzonitrile 2c as colorless solid (4.89 g, 61%).

Details Procedure for Synthesis of 3a-3d

Ruthenium-catalyzed annulations of substituted benzonitrile with Phenyl vinyl sulfone

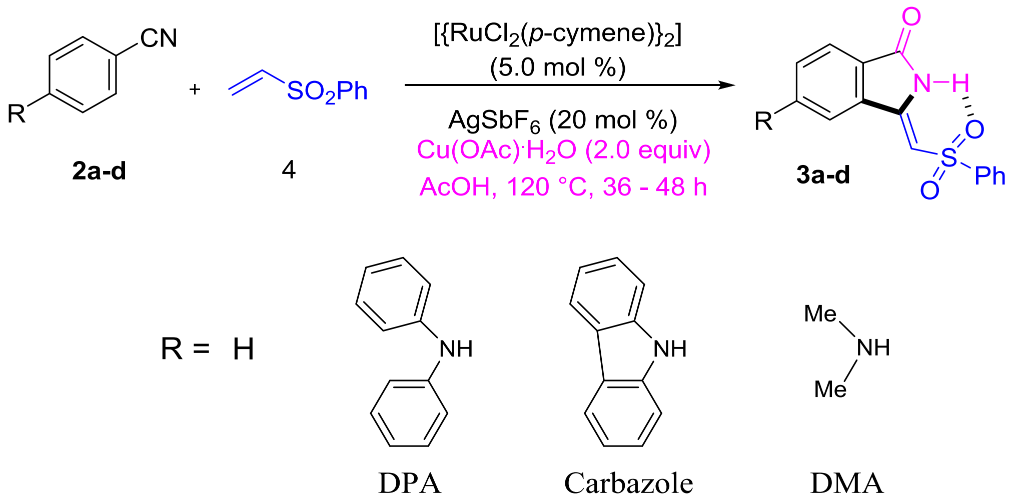

Substituted nitriles **2** (75 mg), phenyl vinyl sulfone **4** (1.20 equiv)), [{$RuCl_2$(*p*-cymene)}$_2$] (5 mol %), $Cu(OAc)_2.H_2O$ (2.0 equiv), $AgSbF_6$ (20 mol %) were taken in a 15-mL pressure tube equipped with a magnetic stirrer and septum (Note: as $AgSbF_6$ is moisture sensitive, thus, $AgSbF_6$ was taken inside the nitrogen glove box). The tube was evacuated and purged with nitrogen gas three times. Then acetic acid (3.0 mL) was added into the reaction mixture and allowed to stir at room temperature for few minutes and again the reaction tube was evacuated and purged with nitrogen gas. This purging of nitrogen gas was repeated three times. Then, the reaction mixture was allowed to stir at 120 °C for 36-48 h and after that the reaction was monitored by TLC checking. After maximum conversion observed by TLC, the reaction mixture was cooled to ambient temperature and the reaction mixture was diluted with CH_2Cl_2, and filtered through the Celite and silica gel and finally collected filtrate was concentrated by rota-evaporator. The crude residue was purified through the silica gel column using hexanes and ethyl acetate as eluent to give pure 3a to 3d.

Spectral Data (Characterization)

4-(Diphenylamino) benzonitrile (2b)

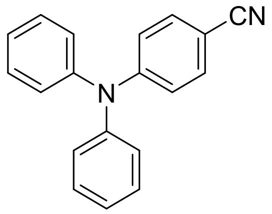

^{1}H NMR ($CDCl_3$, 400 MHz): δ7.44 (d, *J* = 8.8 Hz,2 H), 7.38 – 7.34 (m, 4 H), 7.20 – 7.16 (m, 6 H), 6.98 (d, *J* = 8.8 Hz,2 H).

^{13}C NMR ($CDCl_3$, 100 MHz): δ151.71, 146.08, 133.30, 129.90, 126.29, 125.26, 119.81, 102.61.

HRMS (ESI): calc. for [($C_{19}H_{14}N_2$)H] (M+H) 271.1235, measured 271.1242.

4-(9*H*-Carbazol-9-yl) benzonitrile (2c)

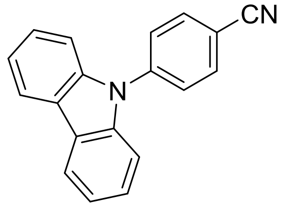

^{1}H NMR ($CDCl_3$, 400 MHz): δ 8.16 (d, *J* = 7.6 Hz,2 H), 7.89 (d, *J* = 8.0 Hz,2 H), 7.72 (d, *J* = 8.0 Hz,2 H), 7.47 – 7.43 (m, 4 H), 7.38 – 7.33 (m, 2 H).

^{13}C NMR ($CDCl_3$, 100 MHz): δ142.14, 139.98, 134.01, 127.17, 126.48, 124.10, 121.11, 120.69, 118.48, 110.55, 109.63.

HRMS (ESI): calc. for [($C_{19}H_{12}N_2$)H] (M+H) 269.1079, measured 269.1067.

(Z)-3-((Phenylsulfonyl)methylene)isoindolin-1-one (3a)

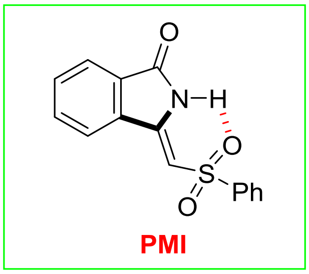

Off white solid; **m.p.** 164-166 °C, eluent (20% ethyl acetate in hexanes).The reaction scale is 75 mg (**2a** (75 mg), **4** (1.2equiv)), 149 mg of **3a** was isolated and yield is 72%. The reaction was done for 36 h at 120 °C.

^{1}H NMR ($CDCl_3$, 400 MHz): δ 9.43 (bs, 1 H), 7.96 – 7.93 (m, 2 H), 7.88 – 7.86 (m, 1 H), 7.64 – 7.53 (m, 6 H), 6.07 (s, 1 H).

^{13}C NMR ($CDCl_3$, 100 MHz): δ167.53, 143.98, 141.58, 135.87, 133.88, 133.28, 132.51, 129.64, 129.04, 127.22, 124.44, 121.48, 100.34.

HRMS (ESI): calc. for [($C_{15}H_{11}NO_3S$)H] (M+H) 286.0538, measured 286.0544.

IR (ATR)$\tilde{\nu}$ (cm^{-1}): 3394, 3063, 2924, 2857, 1728, 1632, 1450, 1379, 1290, 1142, 834, 735, 689.

R_f :0.20(20% ethyl acetate in hexanes).

(Z)-5-(Diphenylamino)-3-((phenylsulfonyl)methylene)isoindolin-1-one(3b)

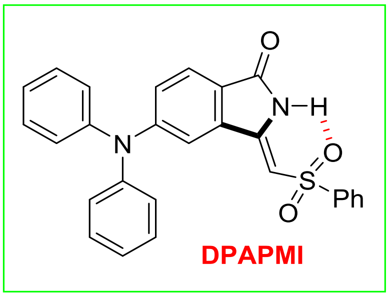

Deep yellow solid; **m.p.** 224-226 °C, eluent (25% ethyl acetate in hexanes). The reaction scale is 75 mg (**2b** (75 mg), **4** (1.2 equiv)), 80 mg of **3b** was isolated and yield is 63 %. The reaction was done for 48 h at 120 °C.

^{1}H NMR ($CDCl_3$, 400 MHz): δ 9.24 (bs, 1 H), 7.92 (d, *J* = 7.2 Hz,2 H), 7.62 (t, *J* = 8.4 Hz,2 H), 7.54 (t, *J* = 7.6 Hz,2 H), 7.33 (t, *J* = 8.0 Hz,4 H), 7.18 – 7.05 (m, 8 H), 5.79 (s, 1 H).

^{13}C NMR ($CDCl_3$, 100 MHz): δ167.45, 153.01, 146.22, 144.41, 141.75, 137.80, 133.72, 130.05, 129.53, 127.20, 126.12, 125.36, 123.71, 120.30, 112.06, 99.42.

HRMS (ESI): calc. for [($C_{27}H_{20}N_2O_3S$)H] (M+H) 453.1273, measured 453.1273.

IR (ATR)ṽ (cm^{-1}): 3395, 3060, 1721, 1588, 1483, 1282, 1142, 1079, 831, 741, 684.

R_f : 0.15 (20% ethyl acetate in hexanes).

(*Z*)-5-(9*H*-Carbazol-9-yl)-3-((phenylsulfonyl)methylene)isoindolin-1-one(3c)

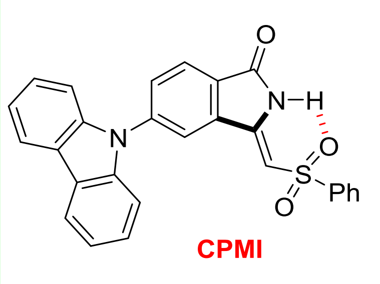

yellow solid; **m.p.** 248-250 °C, eluent (20% ethyl acetate in hexanes). The reaction scale is 75 mg (**2c** (75 mg), **4** (1.2 equiv)), 83 mg of **3c** was isolated and yield is 66 %. The reaction was done for 48 h at 120 °C.

^{1}H NMR ($CDCl_3$, 400 MHz): δ 9.57 (bs, 1 H), 8.13 – 8.11 (m, 3 H), 7.98 – 7.96 (m, 2 H), 7.87 (dd, *J* = 7.6, 1.6 Hz,1 H), 7.82 (d, *J* = 1.2 Hz,1 H), 7.67 – 7.63 (m, 1 H), 7.57 (t, *J* = 7.6 Hz,2 H), 7.44 – 7.38 (m, 4 H), 7.34 – 7.30 (m, 2 H), 6.11 (s, 1 H).

^{13}C NMR ($CDCl_3$, 100 MHz): δ166.66, 143.14, 142.92, 141.34, 140.24, 137.96, 134.06, 130.73, 129.72, 127.36, 127.23, 126.58, 126.20, 124.10, 121.20, 120.78, 119.60, 109.54, 101.29.

HRMS (ESI): calc. for [($C_{27}H_{18}N_2O_3S$)H] (M+H) 451.1116, measured 451.1109.

IR (ATR)ṽ (cm^{-1}): 3391, 3062, 2925, 2857, 1730, 1632,1489, 1447, 1367, 1294, 1227, 1143, 1081, 829, 746, 688.

R_f : 0.23(20% ethyl acetate in hexanes).

(Z)-5-(Dimethylamino)-3-((phenylsulfonyl)methylene)isoindolin-1-one (3d)

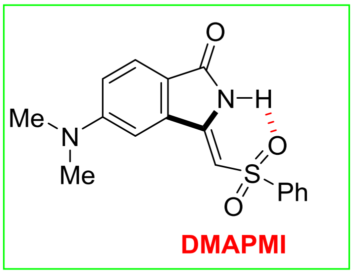

Light brown solid; **m.p.** 268-270 °C, eluent (35% ethyl acetate in hexanes). The reaction scale is 75 mg (**2d** (75 mg), **4** (1.2 equiv)), 89 mg of **3d** was isolated and yield is 53 %. The reaction was done for 48 h at 120 °C.

^{1}H NMR ($CDCl_3$, 400 MHz): δ 9.10 (bs, 1 H), 7.97 – 7.95 (m, 2 H), 7.68 – 7.53 (m, 4 H), 6.80 (dd, *J* = 7.6, 2.0 Hz, 1 H), 6.70 (d, *J* = 2.0 Hz, 1 H), 5.97 (s, 1 H), 3.06 (s, 6 H).

^{13}C NMR ($CDCl_3$, 100 MHz): δ168.25, 153.91, 145.50, 142.17, 138.43, 133.62, 129.54, 127.13, 125.54, 115.64, 114.85, 103.06, 98.25, 40.61.

HRMS (ESI): calc. for [($C_{17}H_{16}N_2O_3S$)H] (M+H) 329.0960, measured 329.0966.

IR (ATR)$\tilde{\nu}$ (cm^{-1}): 3405, 3062, 2924, 1717, 1609, 1445, 1371, 1292, 1143, 830, 738, 693.

R_f : 0.05 (20% ethyl acetate in hexanes).

Note S1:

- ‘R_f’ value indicate that DMAMI (3d) is highest polar, however CPMI (3c) is lowest polar molecule among donor substituted isoindolinone derivatives.
- Melting point of DMAPMI (3d) is highest probably because of high cohesive interactions between the oppositely charged poles of highly polar 3d molecules.

Characterization Figures:

^{1}H NMR data of compound **3a**

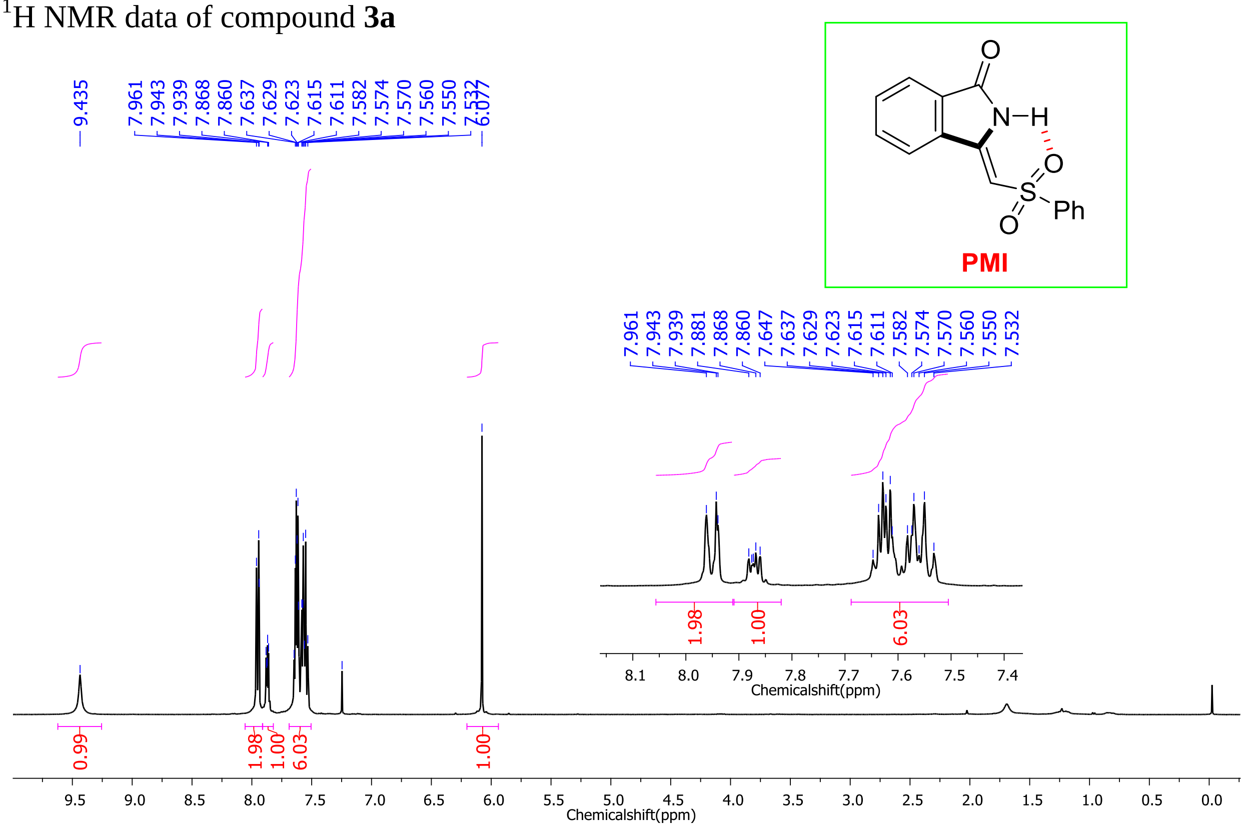

^{13}C NMR data of compound **3a**

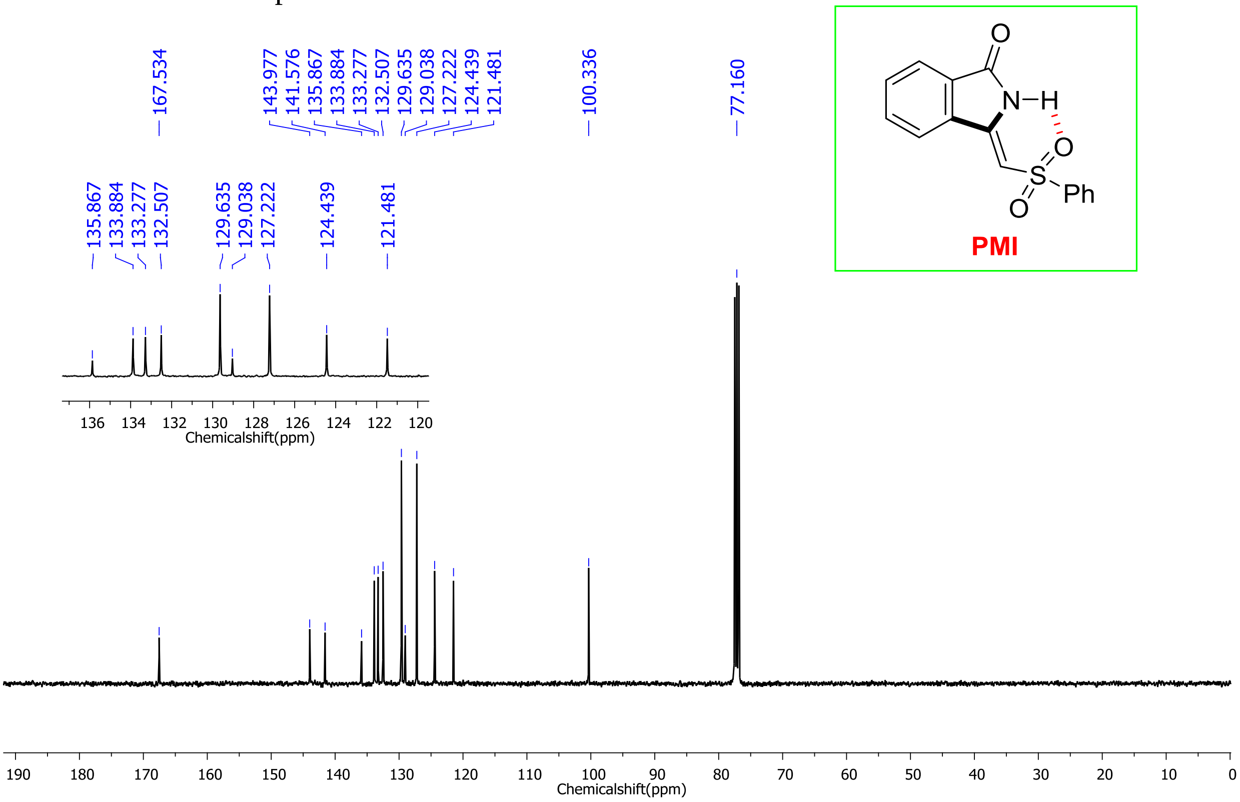

DEPT-135 data of compound **3a**

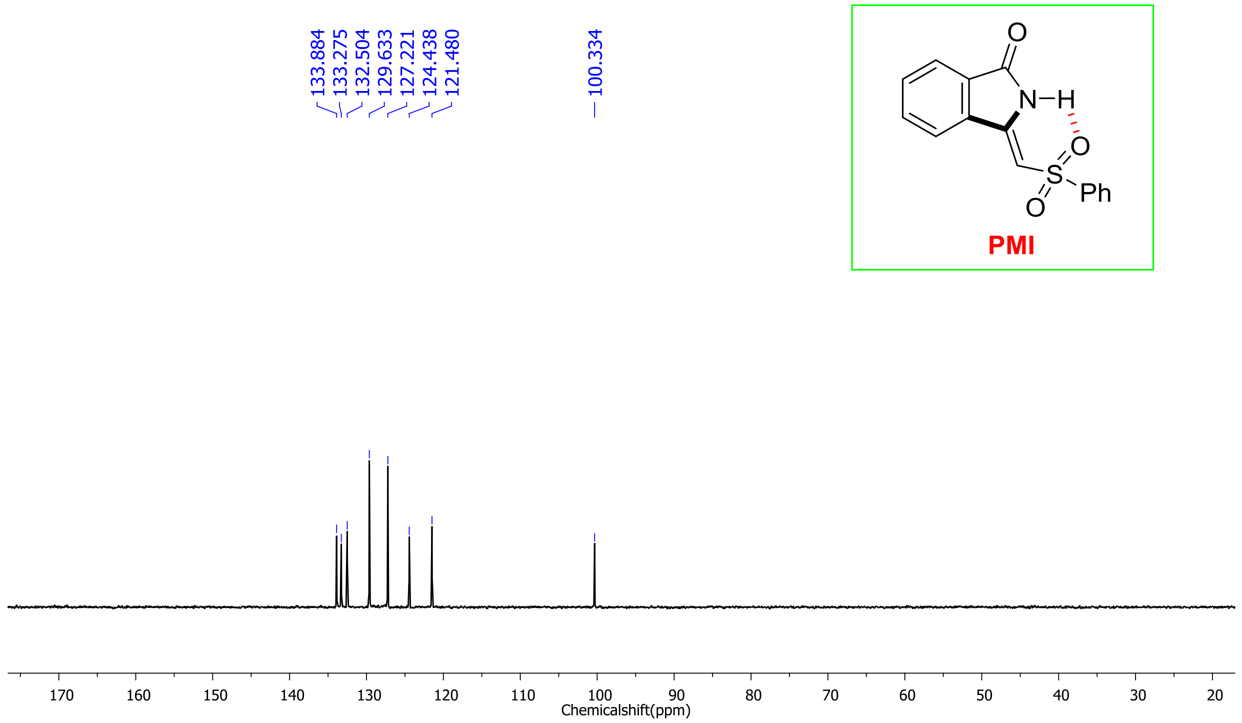

IR data of compound **3a**

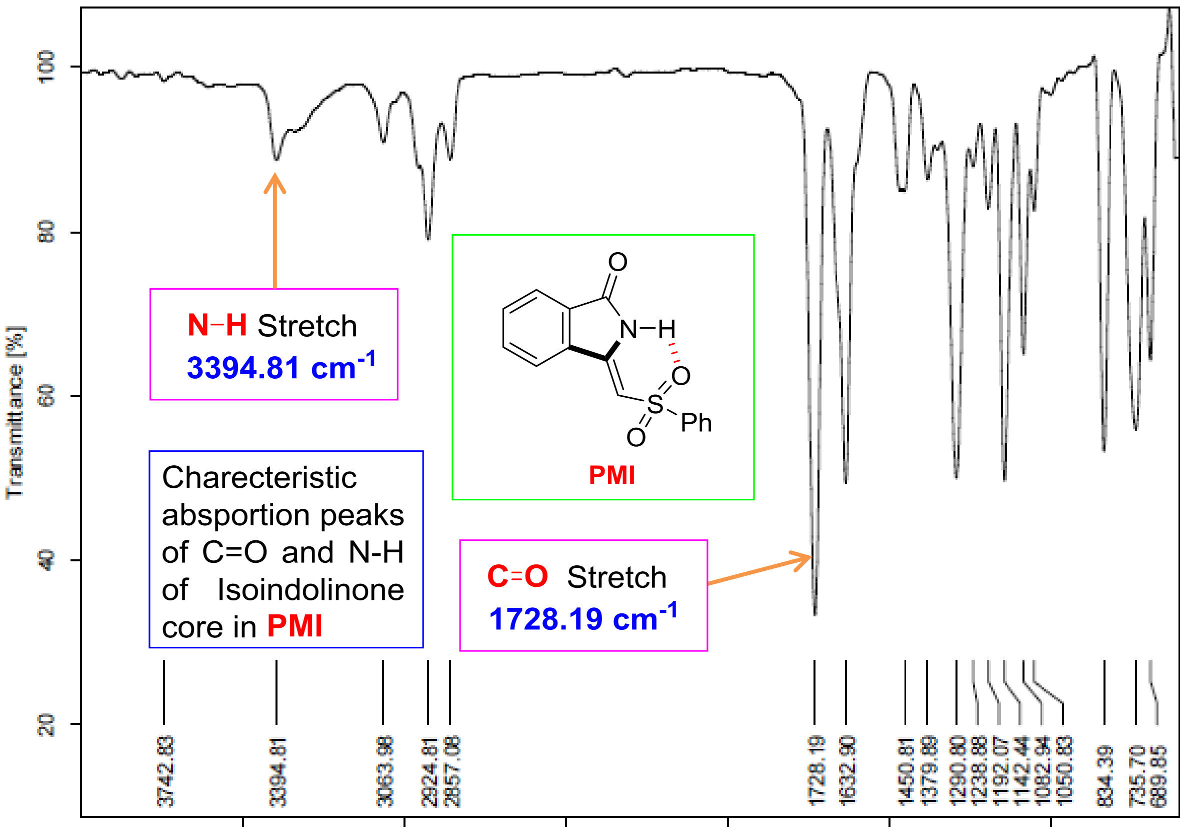

HRMS data of compound **3a**

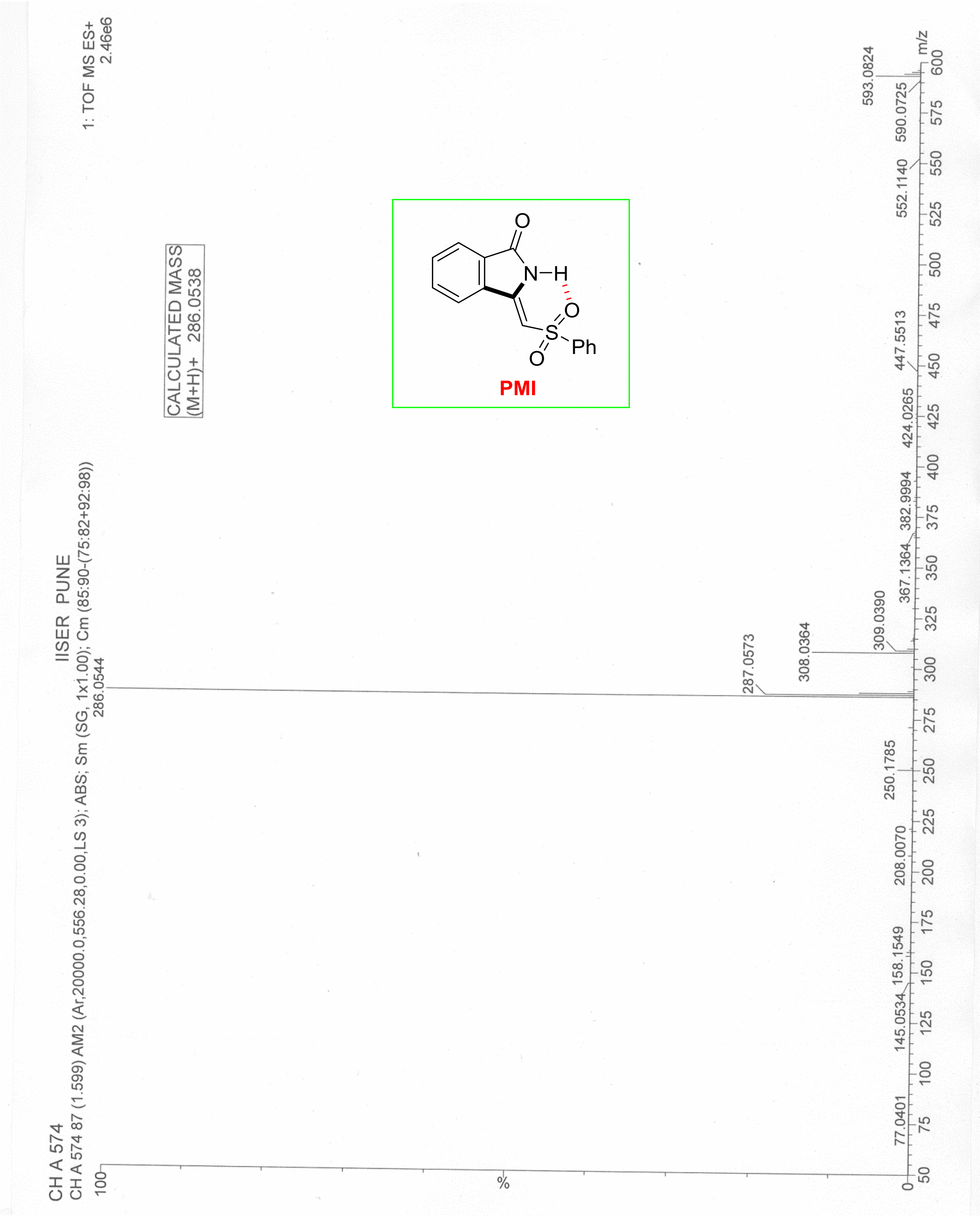

^{1}H NMR data of compound **3b**

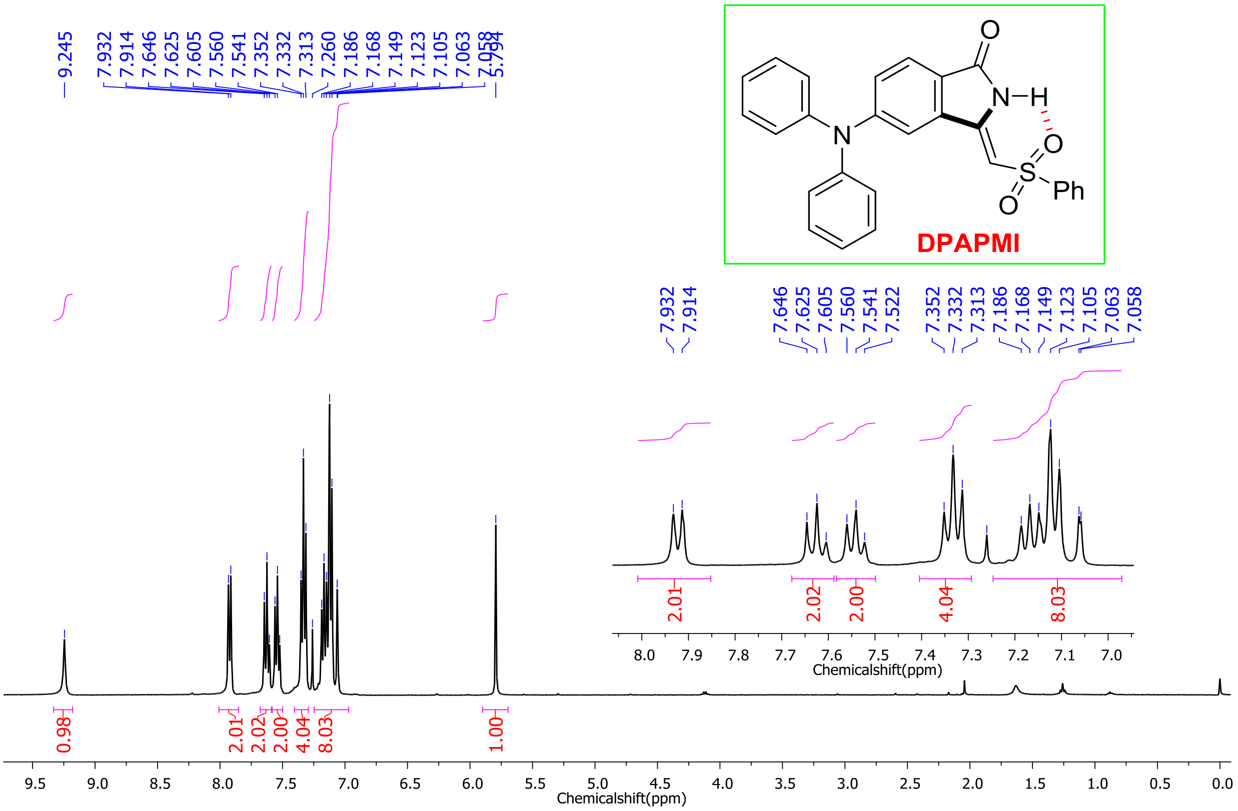

^{13}C NMR data of compound **3b**

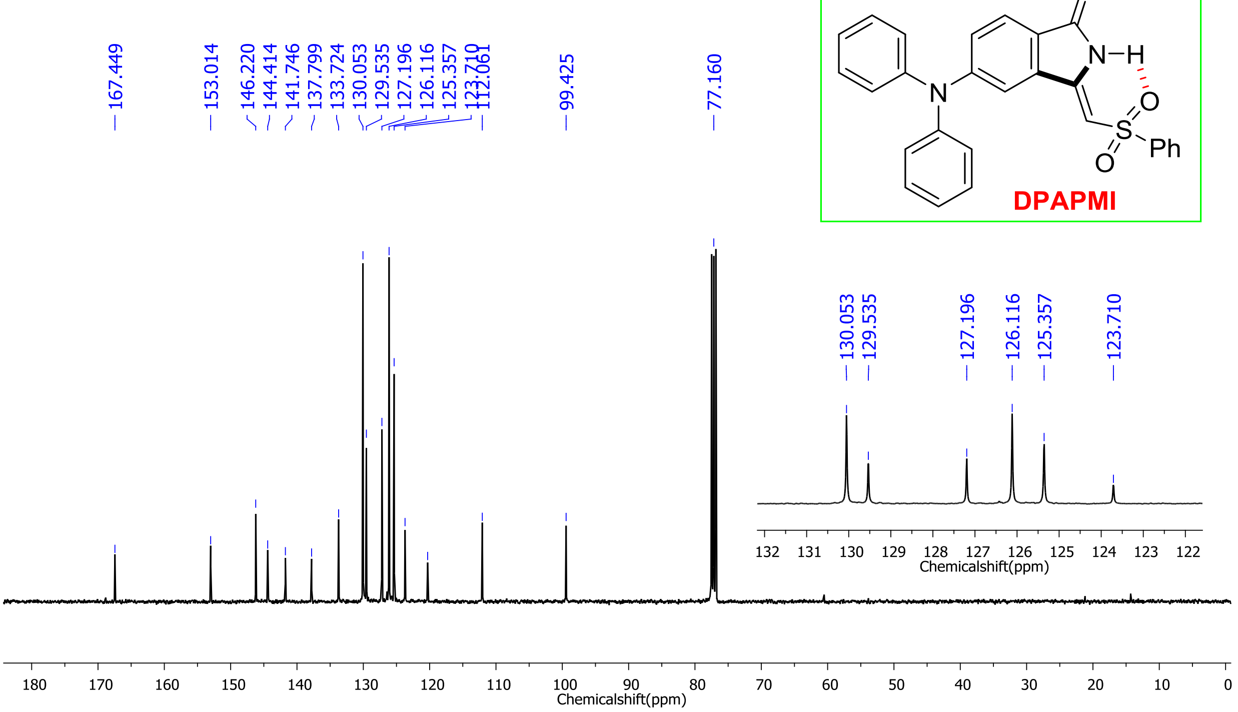

DEPT-135 data of compound **3b**

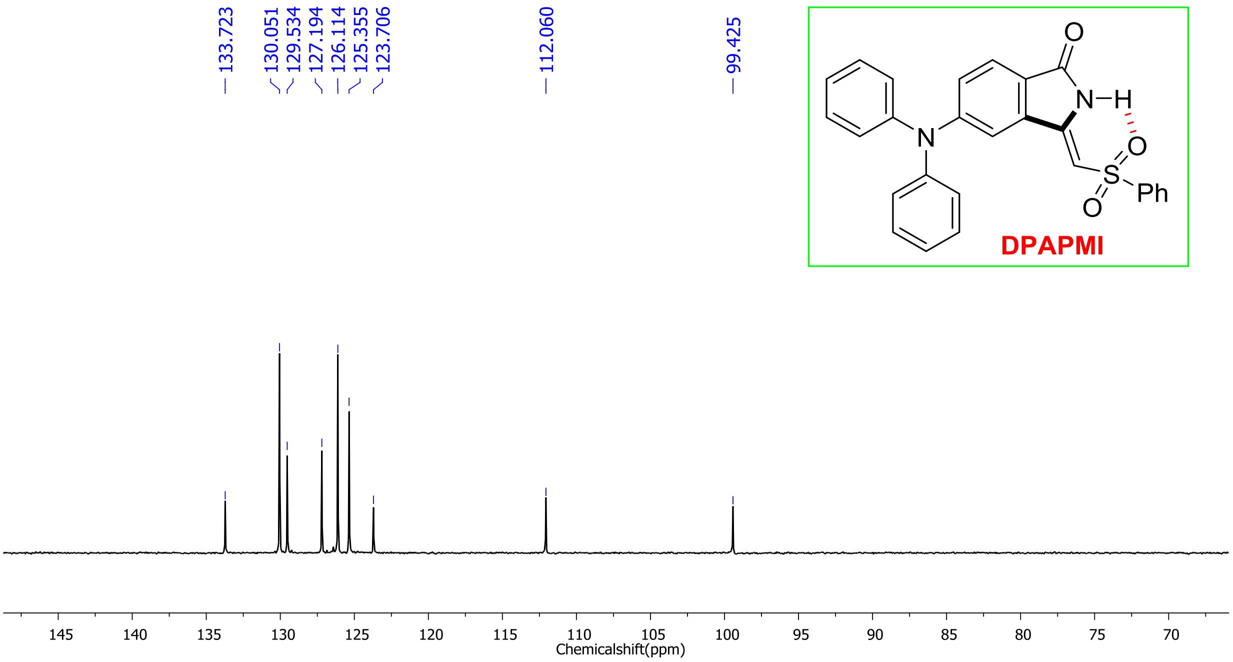

IR data of compound **3b**

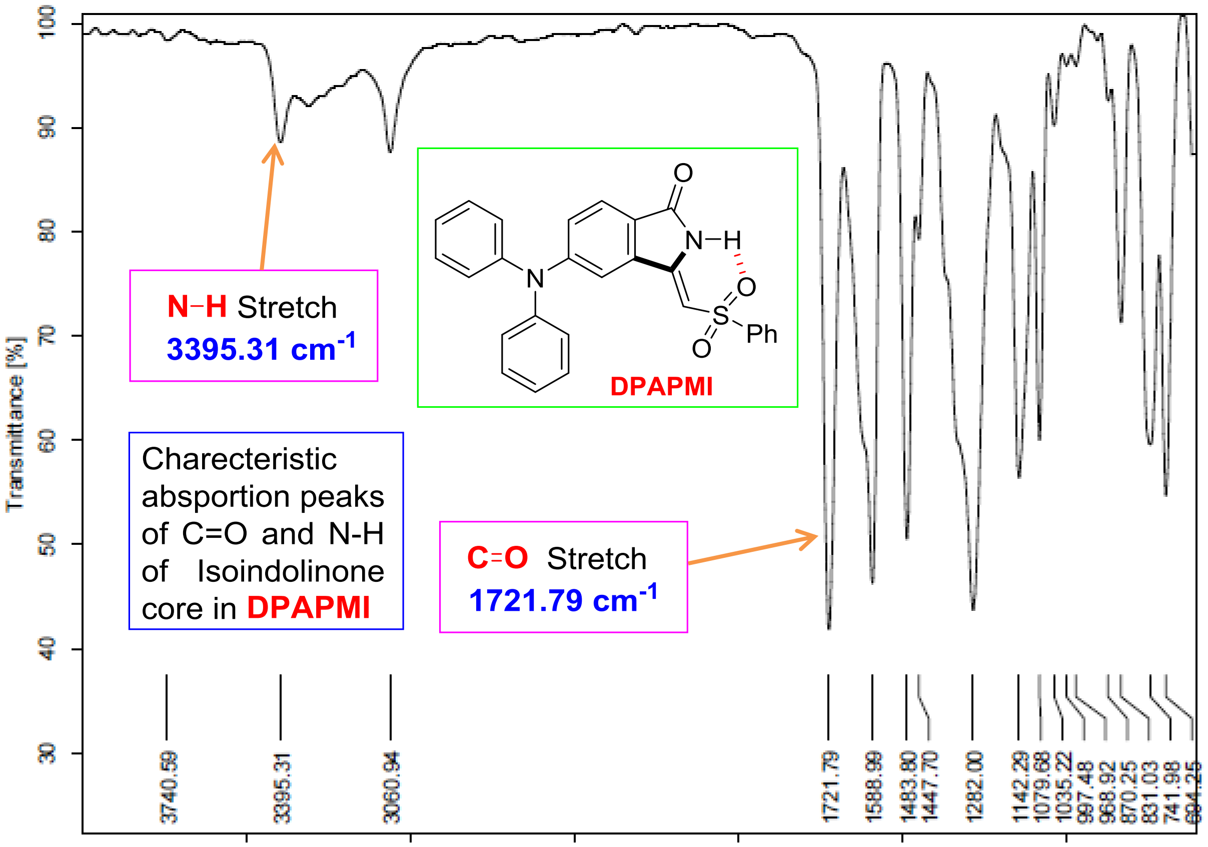

HRMS data of compound **3b**

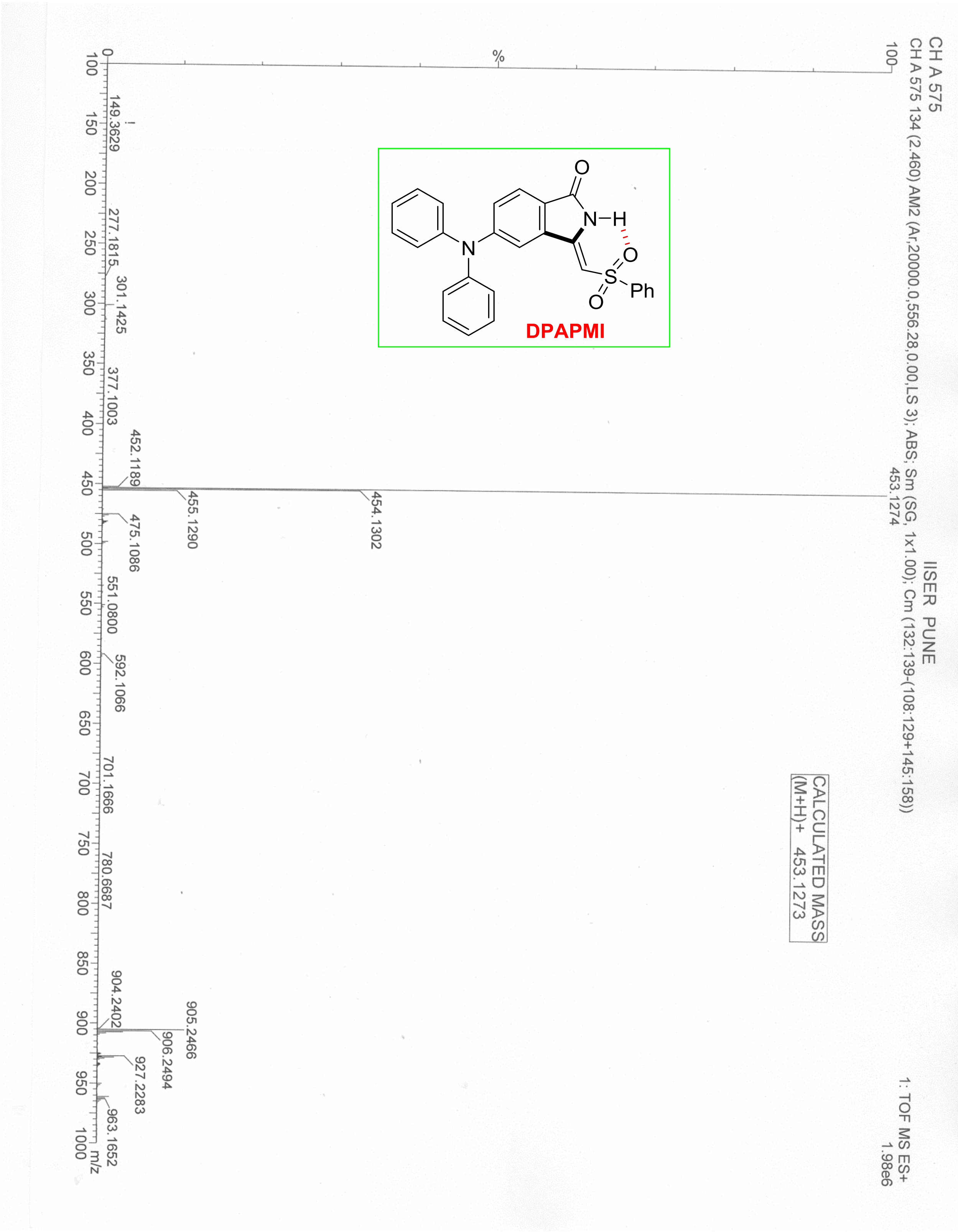

^{1}H NMR data of compound **3c**

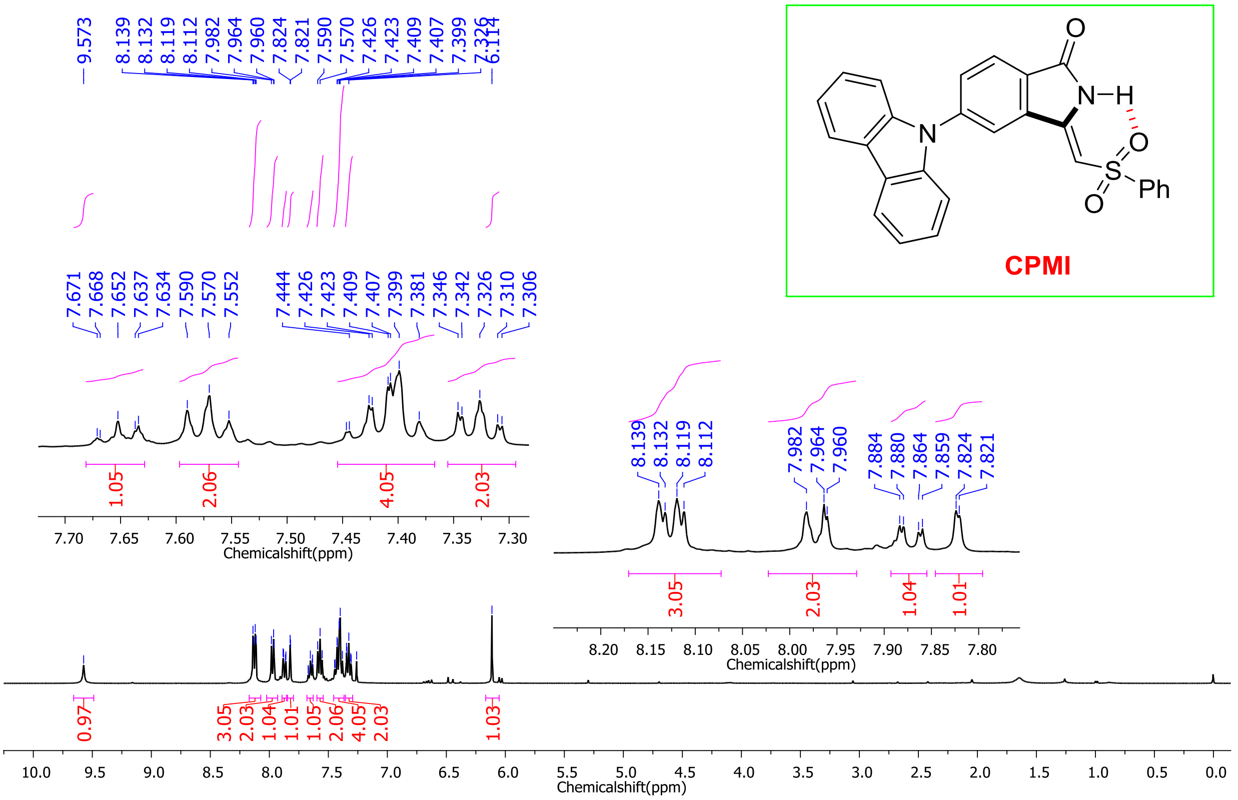

^{13}C NMR data of compound **3c**

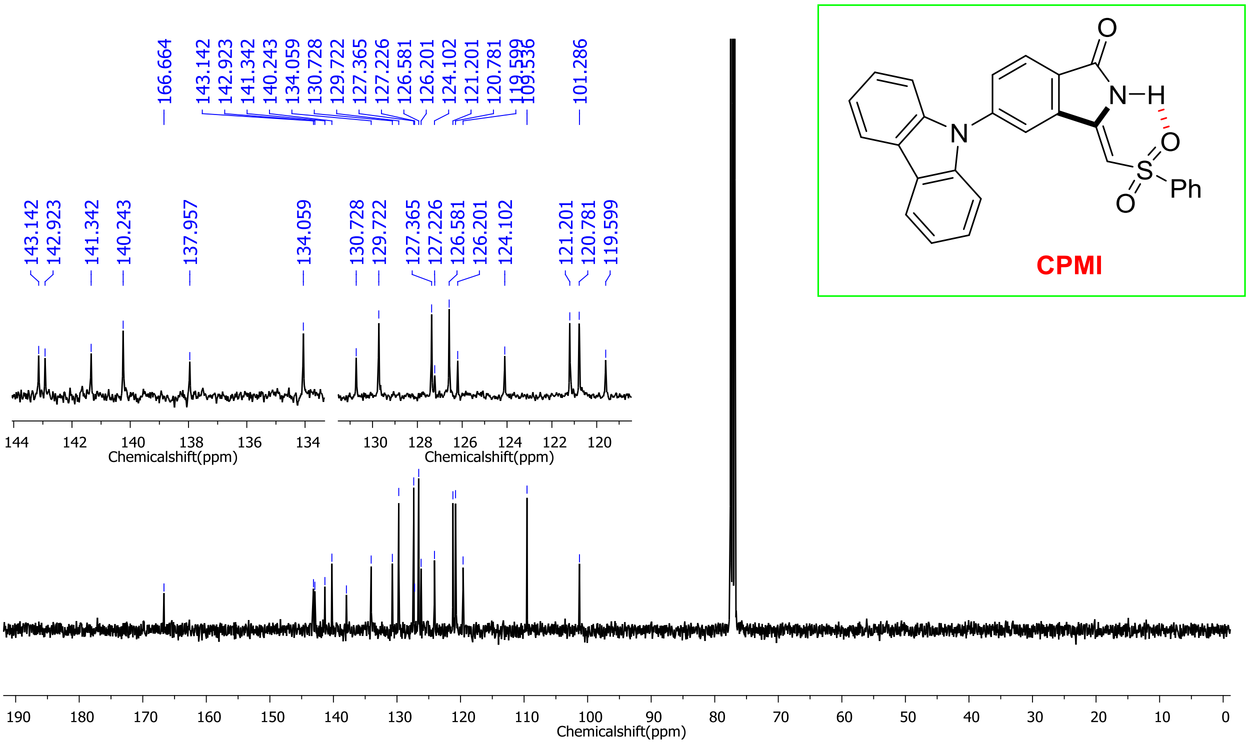

DEPT-135 data of compound **3c**

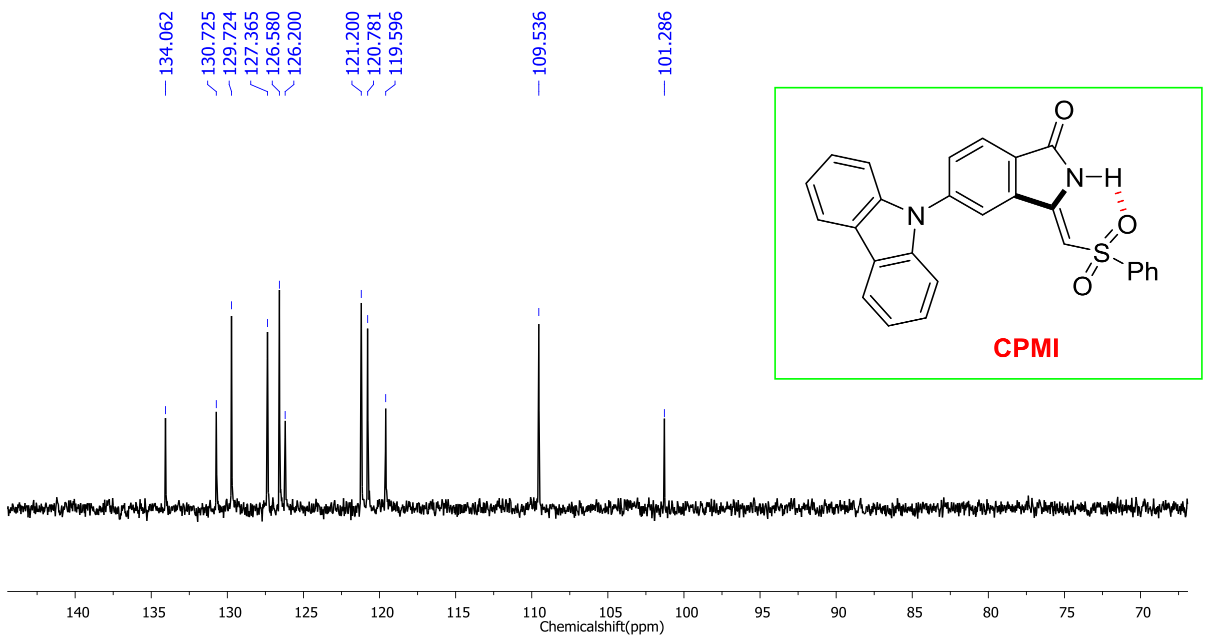

IR data of compound **3c**

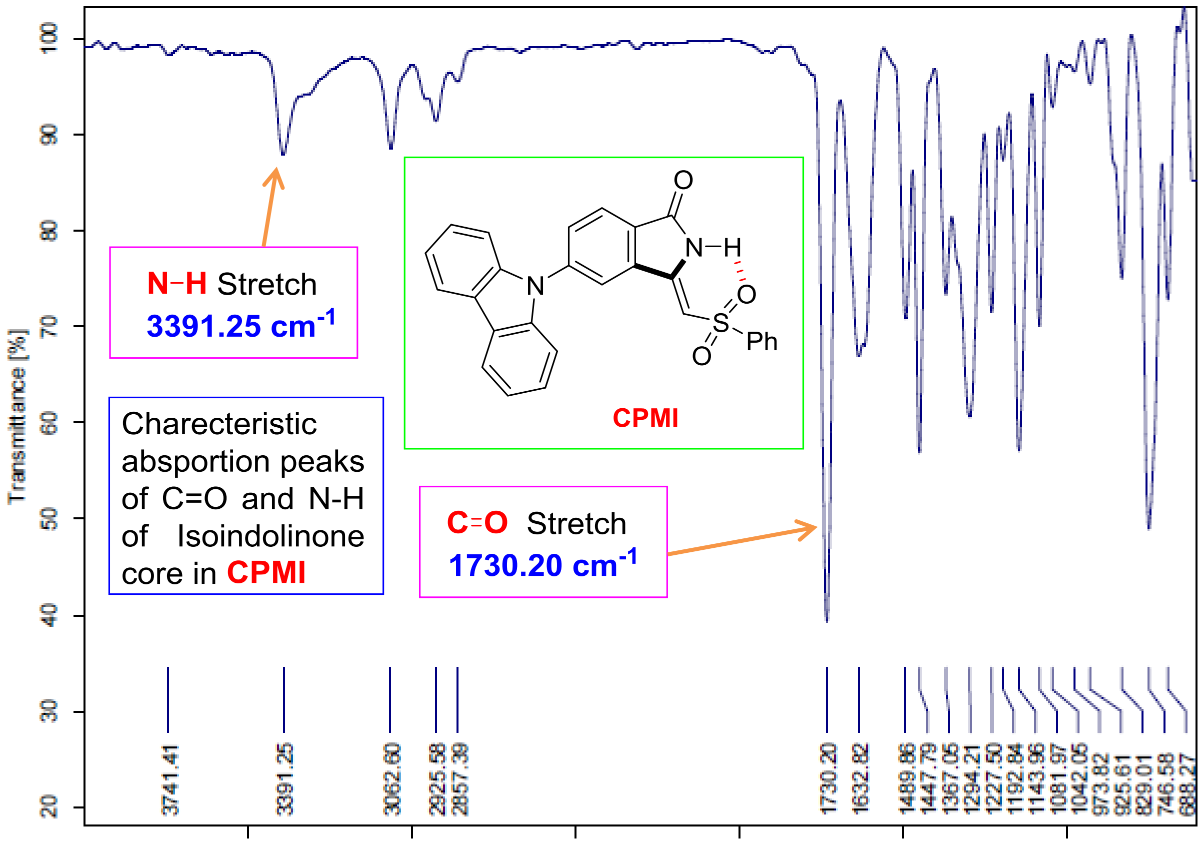

HRMS data of compound **3c**

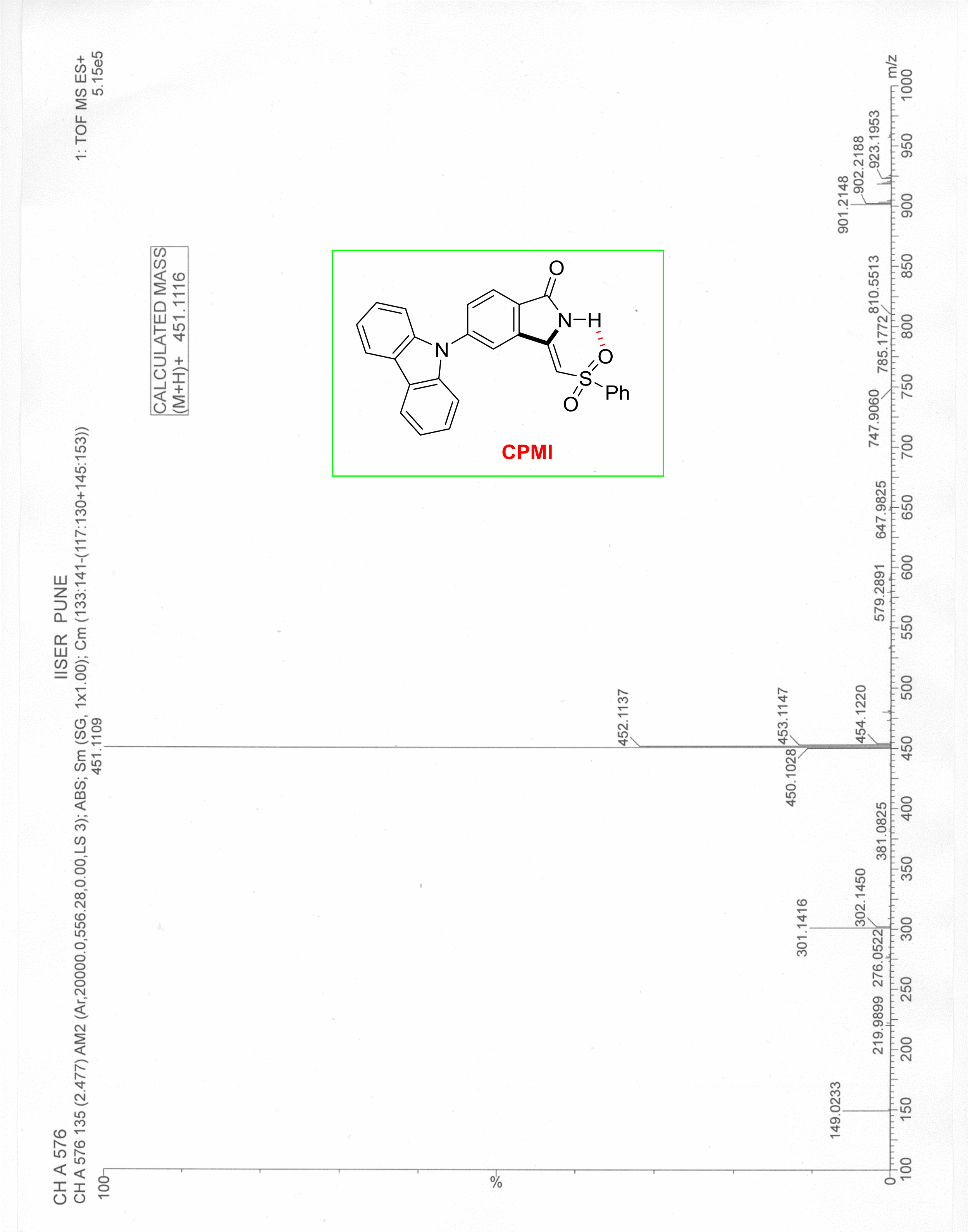

^{1}H NMR data of compound **3d**

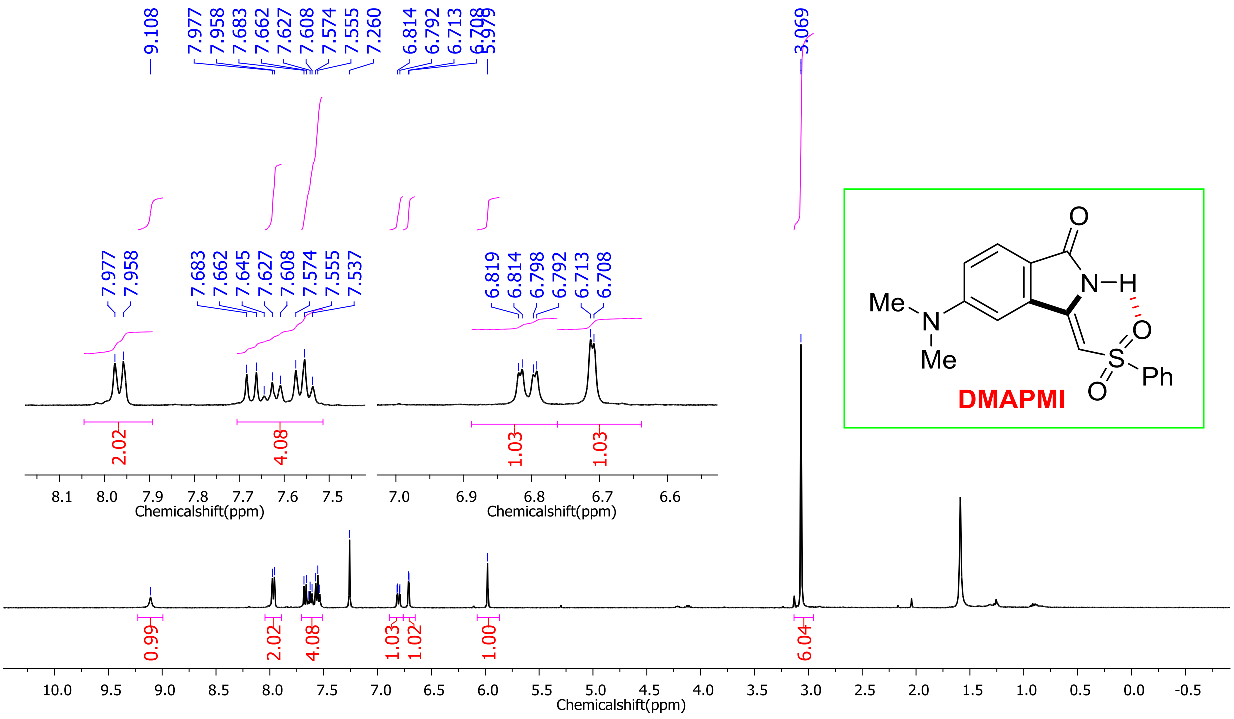

^{13}C NMR data of compound **3d**

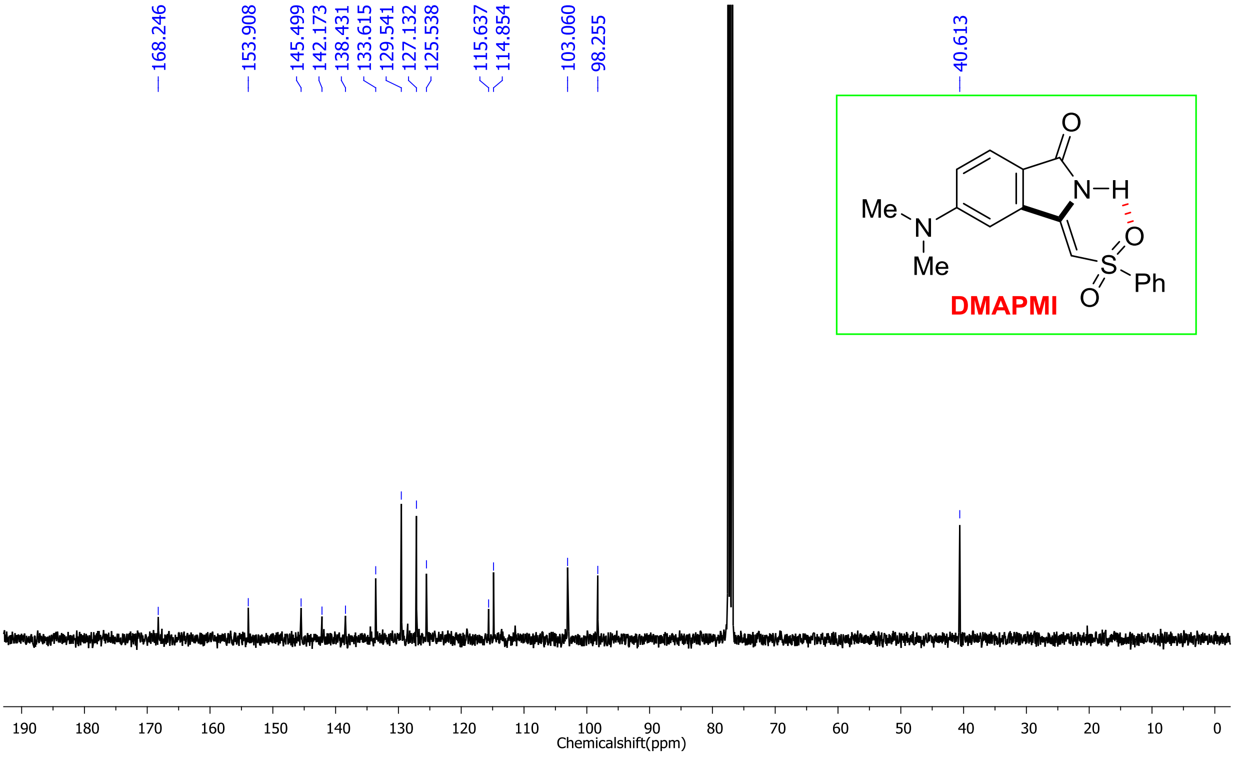

DEPT-135 data of compound **3d**

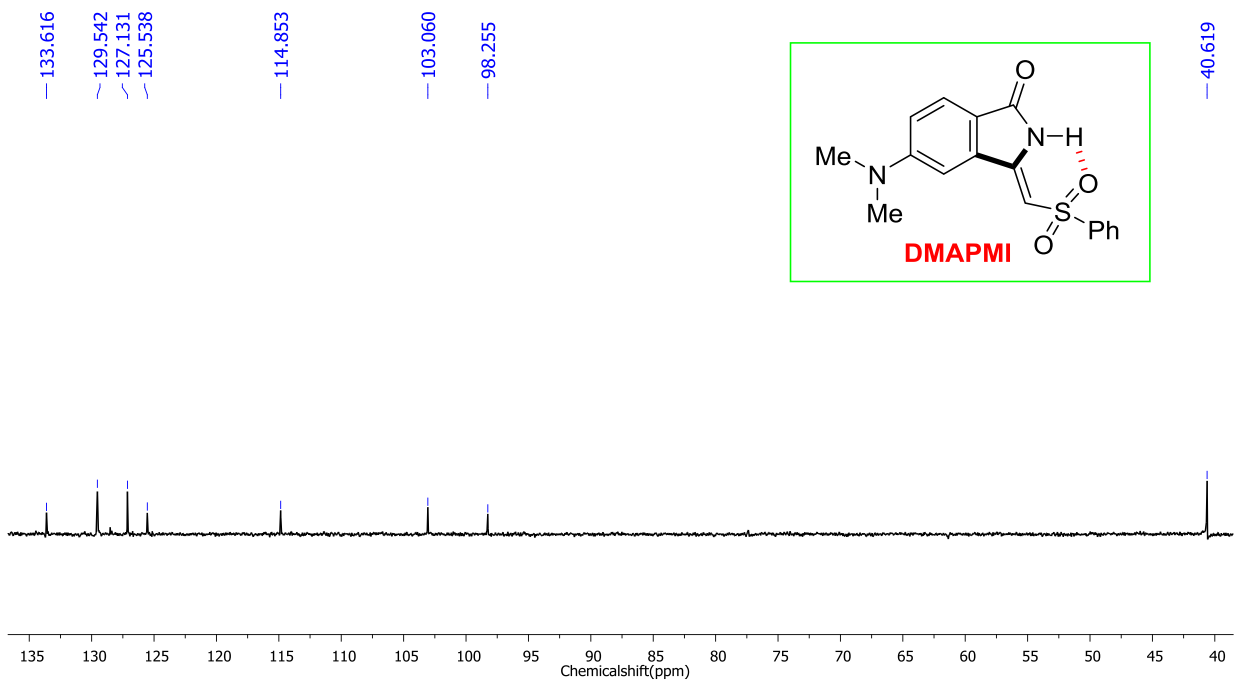

IR data of compound **3d**

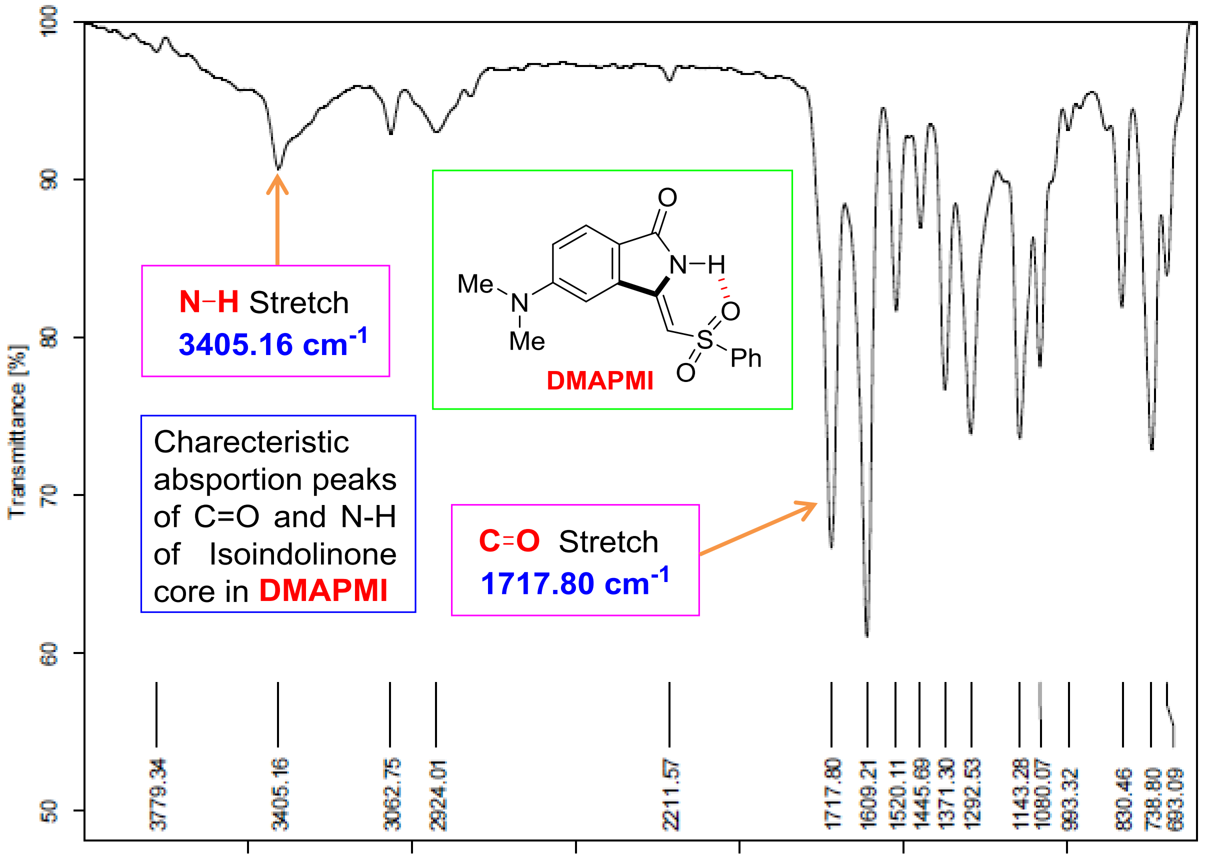

HRMS data of compound **3d**

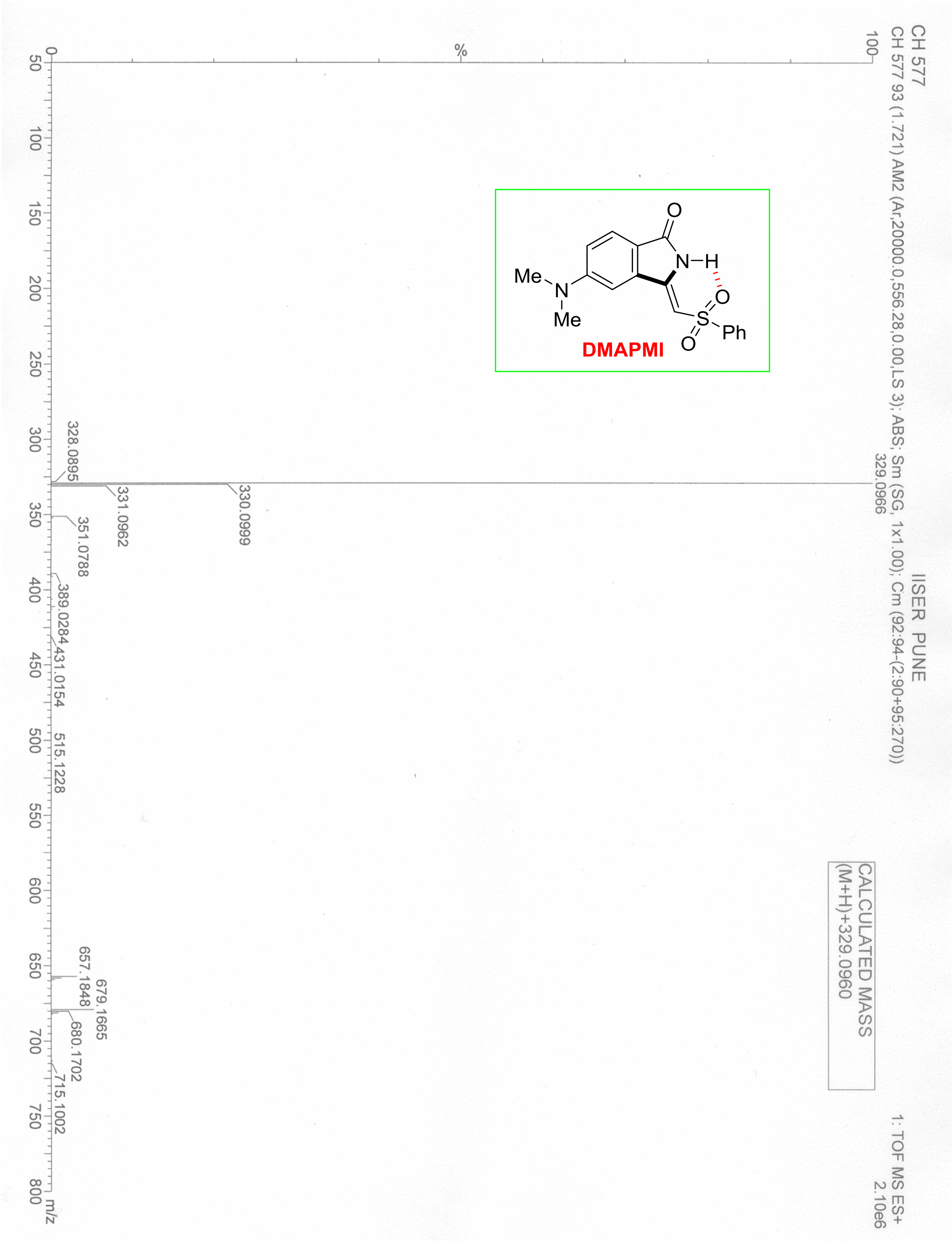

^{1}H NMR data of compound **2b**

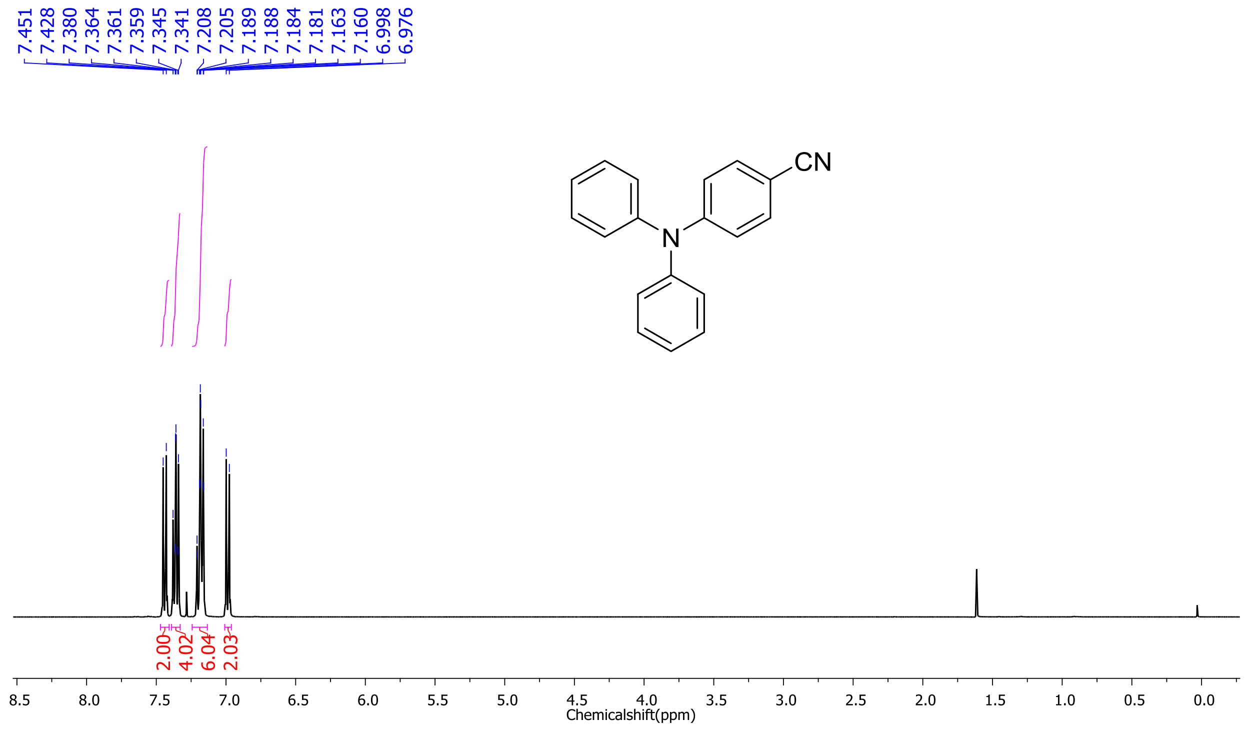

^{13}C NMR data of compound **2b**

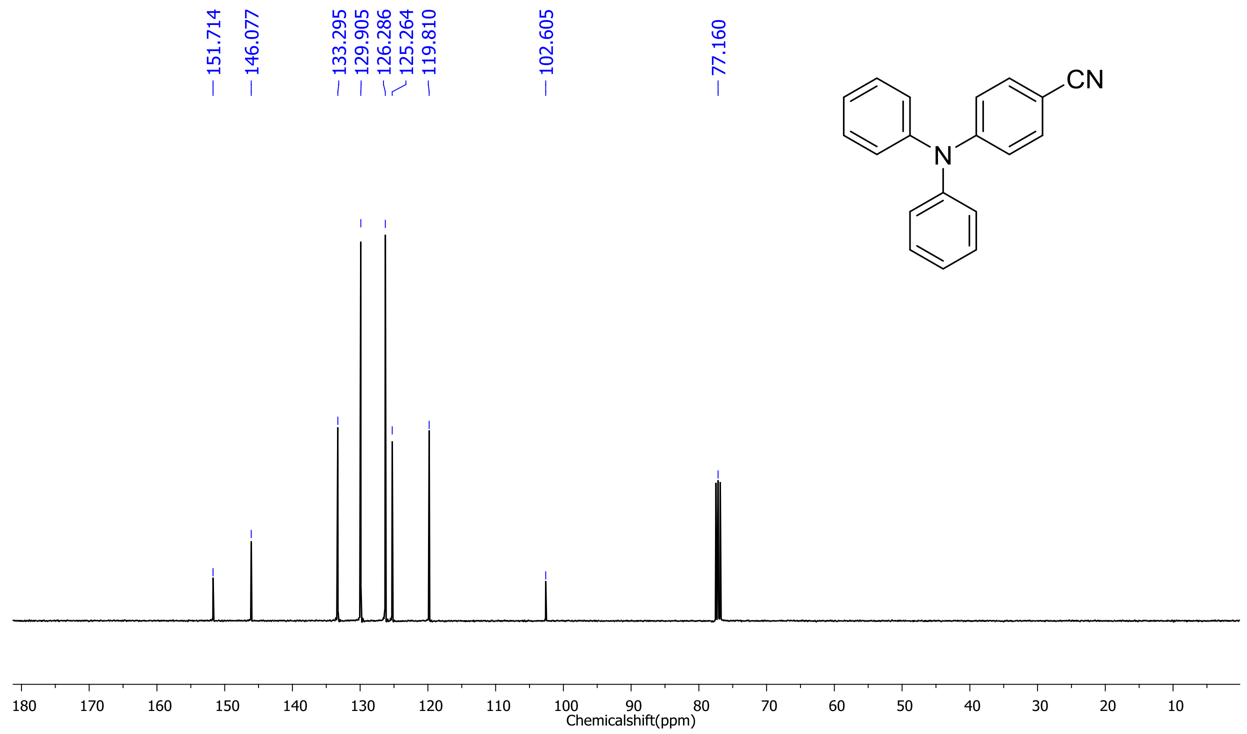

DEPT-135 data of compound **2b**

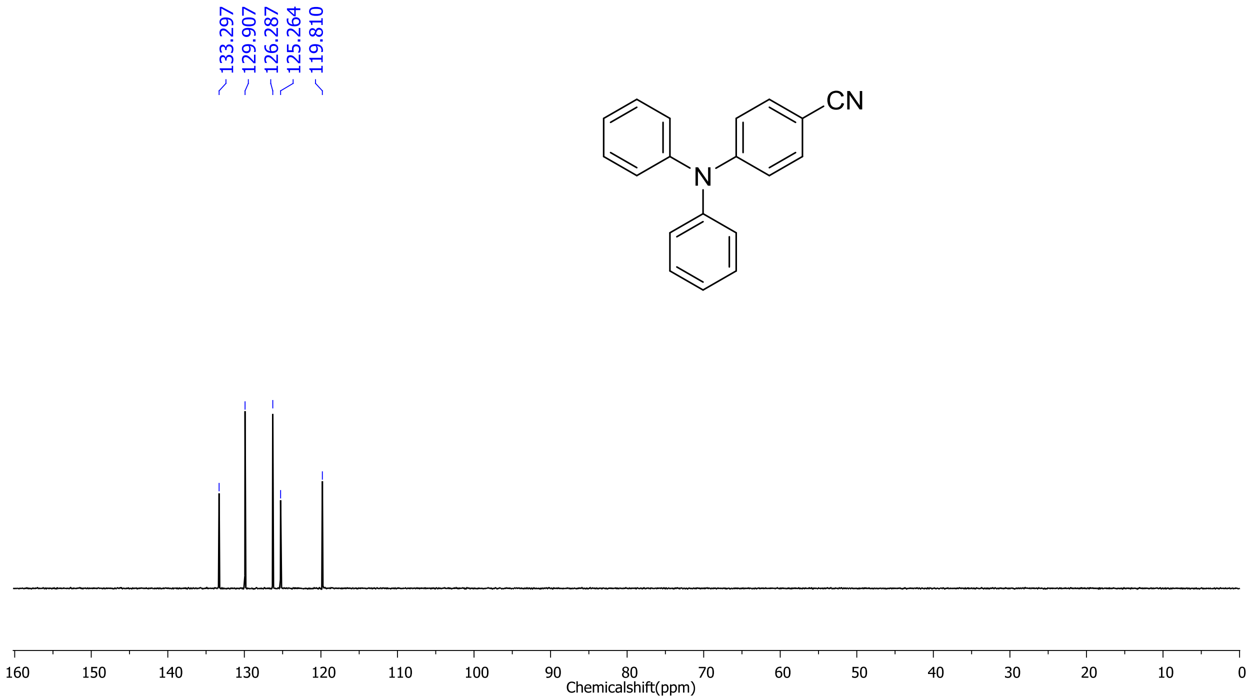

^{1}H NMR data of compound **2c**

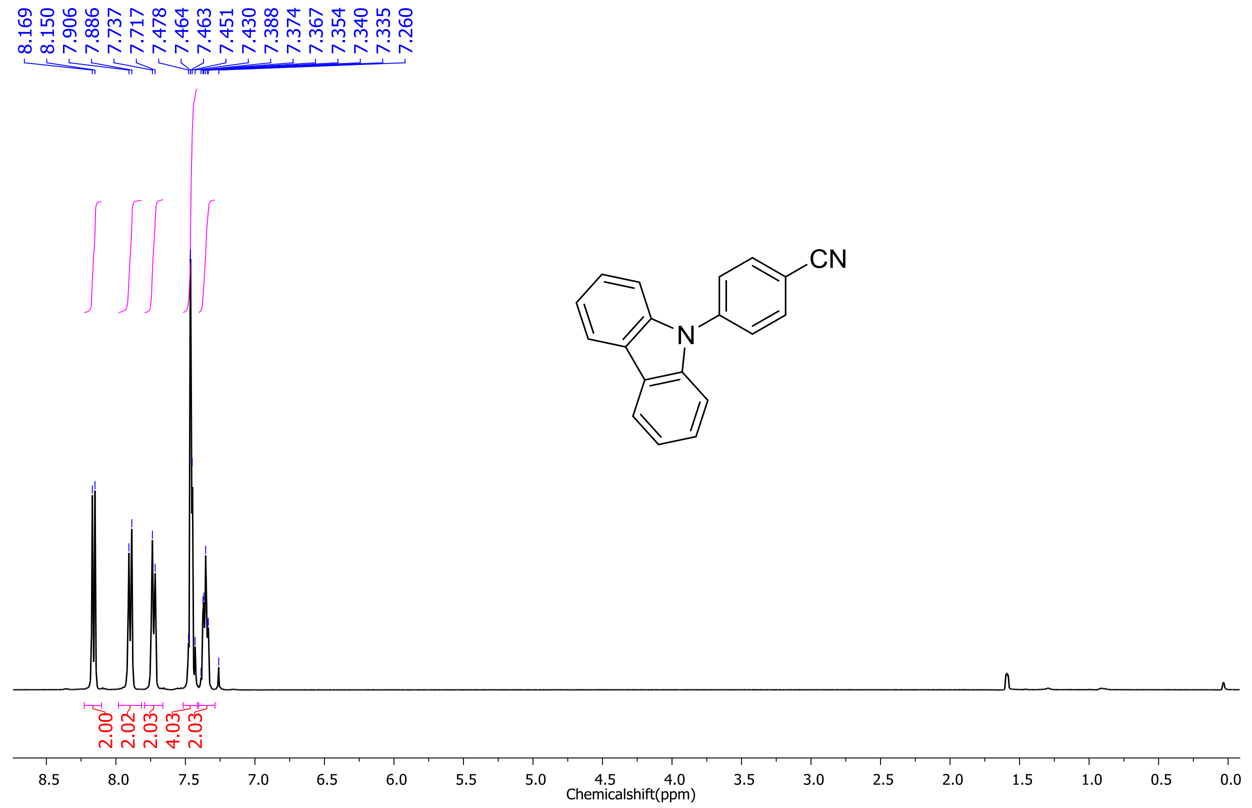

^{13}C NMR data of compound **2c**

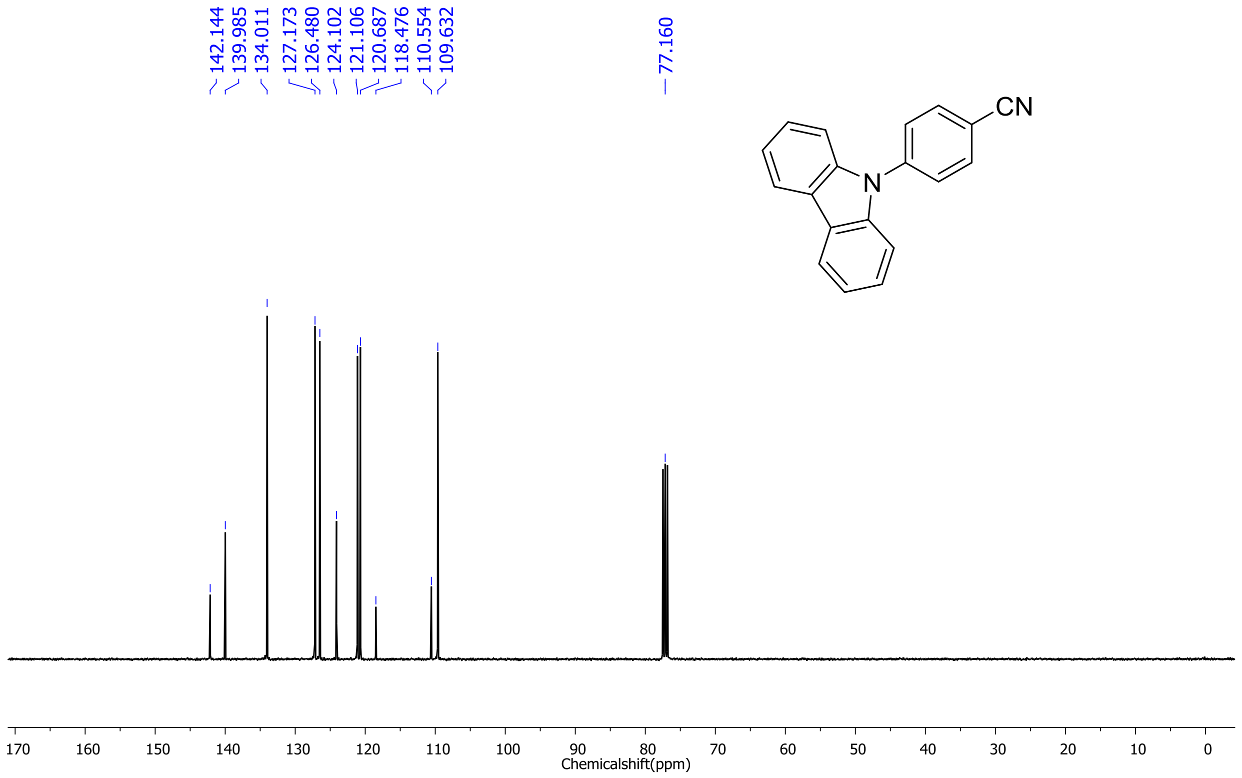

DEPT-135 data of compound **2c**

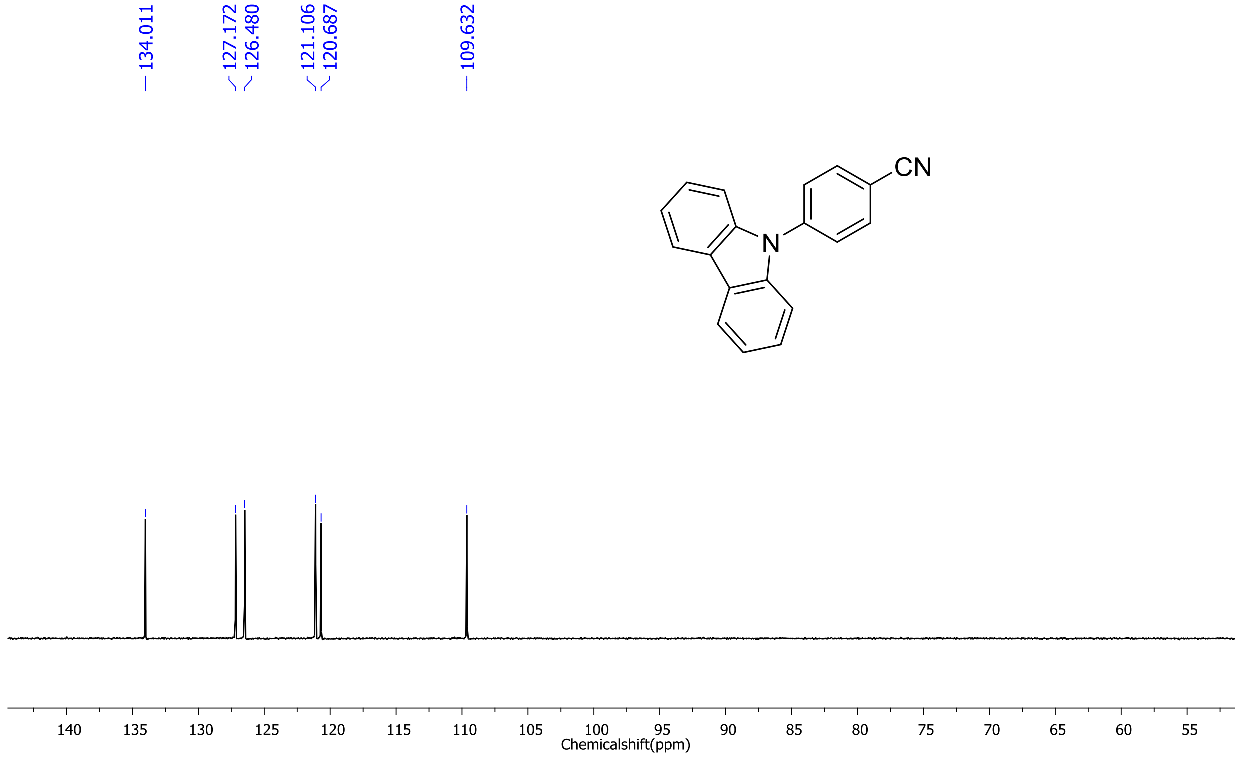

Cartesian Coordinates of the B3LYP/6-31G (d, p) Optimized Structures of Various Isoindolinone Derivatives in Standard XYZ Format (blue circle number indicates charge (0) and spin (1) respectively).

PMI:

0 1	**X**	**Y**	**Z**
C	4.85727	1.61351	-0.30364
C	3.88977	2.42537	0.30414
C	2.60471	1.94508	0.57394
C	2.31345	0.6303	0.22118
C	3.28438	-0.17608	-0.38483
C	4.56166	0.29412	-0.65737
C	1.07874	-0.17725	0.35771
N	1.37614	-1.42001	-0.17217
C	2.6909	-1.51879	-0.64405
O	3.19351	-2.50246	-1.14805
C	-0.09308	0.23865	0.89009
S	-1.5275	-0.77633	1.06985
O	-1.22147	-2.09959	0.48007
O	-1.99971	-0.66191	2.4574
C	-2.76106	0.00669	0.01979
C	-3.64164	0.93151	0.58398
C	-4.59325	1.54213	-0.23312
C	-4.65748	1.22427	-1.59136

C	-3.77594	0.28941	-2.14046
C	-2.81997	-0.32749	-1.33512
H	5.84602	2.01689	-0.49902
H	4.14297	3.44674	0.57145
H	1.86642	2.58565	1.0463
H	5.29608	-0.35137	-1.12807
H	0.70323	-2.17884	-0.16406
H	-0.20021	1.23606	1.29779
H	-3.58852	1.14484	1.64599
H	-5.28836	2.25914	0.1927
H	-5.40148	1.7004	-2.22296
H	-3.83767	0.03534	-3.19411
H	-2.14088	-1.07028	-1.73909

DPAPMI:

0 1	**X**	**Y**	**Z**
C	2.42145	-2.16197	0.2913
C	2.1179	-0.78089	0.23911
C	0.77645	-0.35896	0.36074
C	-0.20533	-1.32163	0.53834
C	0.10604	-2.68606	0.57779
C	1.42157	-3.11632	0.45117

C	-1.67526	-1.19299	0.69407
N	-2.15796	-2.48191	0.81172
C	-1.14717	-3.45717	0.76049
O	-1.32136	-4.65658	0.84853
C	-2.37589	-0.03533	0.71509
S	-4.12022	0.07332	0.95472
O	-4.65538	-1.30507	1.03481
O	-4.37984	1.04347	2.0291
C	-4.73489	0.79652	-0.57489
C	-4.88649	2.18177	-0.65767
C	-5.35336	2.73965	-1.84807
C	-5.66492	1.91666	-2.93234
C	-5.51865	0.53067	-2.83163
C	-5.05171	-0.03914	-1.64819
N	3.1452	0.16646	0.06907
C	4.31173	-0.14903	-0.69114
C	5.58289	0.16431	-0.18908
C	3.32596	2.60495	-0.11735
C	3.03897	1.46669	0.64968
C	4.20084	-0.75552	-1.95124
C	5.34624	-1.05478	-2.68656
C	6.61116	-0.73625	-2.18831
C	6.72175	-0.12146	-0.93974
C	3.24265	3.87326	0.45437

C	2.85753	4.02505	1.78772
C	2.56548	2.89287	2.55098
C	2.66408	1.61958	1.99301
H	3.45623	-2.47365	0.2107
H	0.53633	0.69671	0.31179
H	1.66127	-4.174	0.49391
H	-3.14063	-2.67402	0.97075
H	-1.87728	0.92302	0.64111
H	-4.66143	2.79921	0.20508
H	-5.48111	3.81502	-1.92528
H	-6.03034	2.35524	-3.85611
H	-5.77469	-0.10696	-3.67216
H	-4.94979	-1.11381	-1.54404
H	5.66975	0.63147	0.78622
H	3.61571	2.48773	-1.1563
H	3.21805	-0.98895	-2.34749
H	5.24657	-1.52531	-3.6603
H	7.50067	-0.9639	-2.76735
H	7.70026	0.12676	-0.53931
H	3.4687	4.74658	-0.15039
H	2.78718	5.01471	2.22812
H	2.2732	2.99744	3.59163
H	2.4528	0.74032	2.59276

CPMI:

0 1	**X**	**Y**	**Z**
C	2.16386	2.44543	-0.10638
C	1.972	1.05164	-0.17339
C	0.68541	0.5137	-0.34255
C	-0.38183	1.39641	-0.45157
C	-0.18461	2.77971	-0.37256
C	1.08202	3.32052	-0.19488
C	-1.83449	1.15812	-0.62828
N	-2.42394	2.40925	-0.64632
C	-1.5041	3.4566	-0.50321
O	-1.77109	4.64095	-0.49062
C	-2.43685	-0.04697	-0.7432
S	-4.17062	-0.27678	-0.99917
O	-4.81133	1.05757	-0.96503
O	-4.34856	-1.16749	-2.15489
C	-4.71812	-1.17348	0.46081
C	-4.77004	-2.56796	0.41992
C	-5.18753	-3.26023	1.55684
C	-5.54837	-2.56016	2.70997
C	-5.50095	-1.16381	2.73296
C	-5.08452	-0.45987	1.60425
N	3.08477	0.18502	-0.06737
C	3.41304	-0.84575	-0.96259

C	4.59192	-1.48784	-0.5085
C	4.99261	-0.82208	0.71403
C	4.04563	0.2038	0.95634
C	2.7854	-1.2246	-2.15211
C	3.34405	-2.27873	-2.87227
C	4.50213	-2.9374	-2.42741
C	5.13236	-2.54395	-1.25073
C	6.04653	-1.01508	1.61428
C	6.13766	-0.19745	2.73661
C	5.18026	0.80327	2.97079
C	4.12161	1.01659	2.09016
H	3.17252	2.82869	4.36E-04
H	0.55153	-0.562	-0.36939
H	1.22016	4.39565	-0.14468
H	-3.42088	2.52773	-0.79179
H	-1.86189	-0.96467	-0.73914
H	-4.50805	-3.08956	-0.49413
H	-5.23802	-4.3445	1.5389
H	-5.87481	-3.10351	3.59163
H	-5.79467	-0.62301	3.62723
H	-5.05925	0.62426	1.59593
H	1.90057	-0.71352	-2.51466
H	2.87312	-2.59184	-3.79919
H	4.91076	-3.75598	-3.01153

H	6.03532	-3.04512	-0.91432
H	6.78003	-1.79694	1.44019
H	6.95163	-0.33599	3.44126
H	5.26168	1.42429	3.8578
H	3.38172	1.78383	2.28841

DMAPMI:

0 1	**X**	**Y**	**Z**
C	4.31869	-0.36807	-0.58862
C	3.68045	0.8028	-0.09063
C	2.30551	0.72932	0.25688
C	1.64197	-0.47673	0.10277
C	2.28664	-1.61829	-0.3865
C	3.63096	-1.56664	-0.73566
C	0.2292	-0.84496	0.37571
N	0.11204	-2.17691	0.03455
C	1.31755	-2.73455	-0.43337
O	1.46365	-3.8888	-0.78545
C	-0.731	-0.01995	0.85611
S	-2.39196	-0.50543	1.19226
O	-2.52632	-1.93965	0.85029
O	-2.75539	-0.01311	2.52983
C	-3.37937	0.41882	0.00246
C	-3.92913	1.64422	0.38262

C	-4.6863	2.36144	-0.54417
C	-4.8874	1.85172	-1.82853
C	-4.33917	0.61902	-2.19254
C	-3.58042	-0.10694	-1.27584
N	4.38048	1.98183	0.05165
C	5.78609	2.04941	-0.31441
C	3.7145	3.16893	0.56134
H	5.36565	-0.33344	-0.86034
H	1.78447	1.5979	0.63827
H	4.13385	-2.45019	-1.11614
H	-0.74908	-2.69312	0.17326
H	-0.51086	1.00895	1.11122
H	-3.77907	2.00872	1.393
H	-5.12499	3.3132	-0.26044
H	-5.4795	2.41216	-2.54591
H	-4.50871	0.21964	-3.18774
H	-3.16366	-1.07436	-1.53375
H	5.9443	1.82331	-1.37734
H	6.15555	3.05773	-0.12803
H	6.39635	1.35286	0.27551
H	3.31577	3.01112	1.57227
H	4.43068	3.98908	0.60945
H	2.88484	3.48417	-0.08603

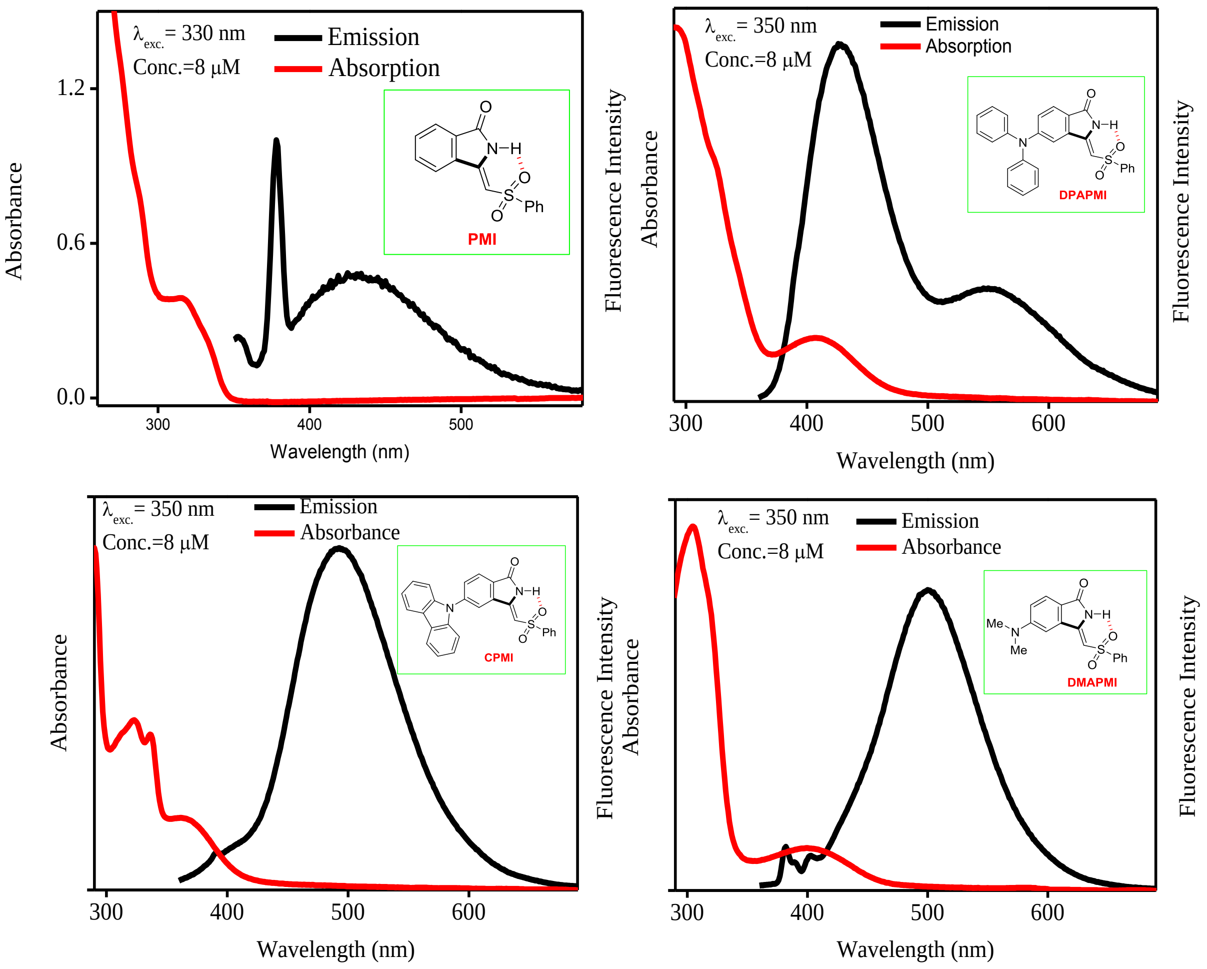

Figure S1: Absorption and emission spectra of PMI and its derivatives in THF solvent. The excitation wavelength is given in each case. The concentration of each luminogen kept constant 8 μM during all measurements.

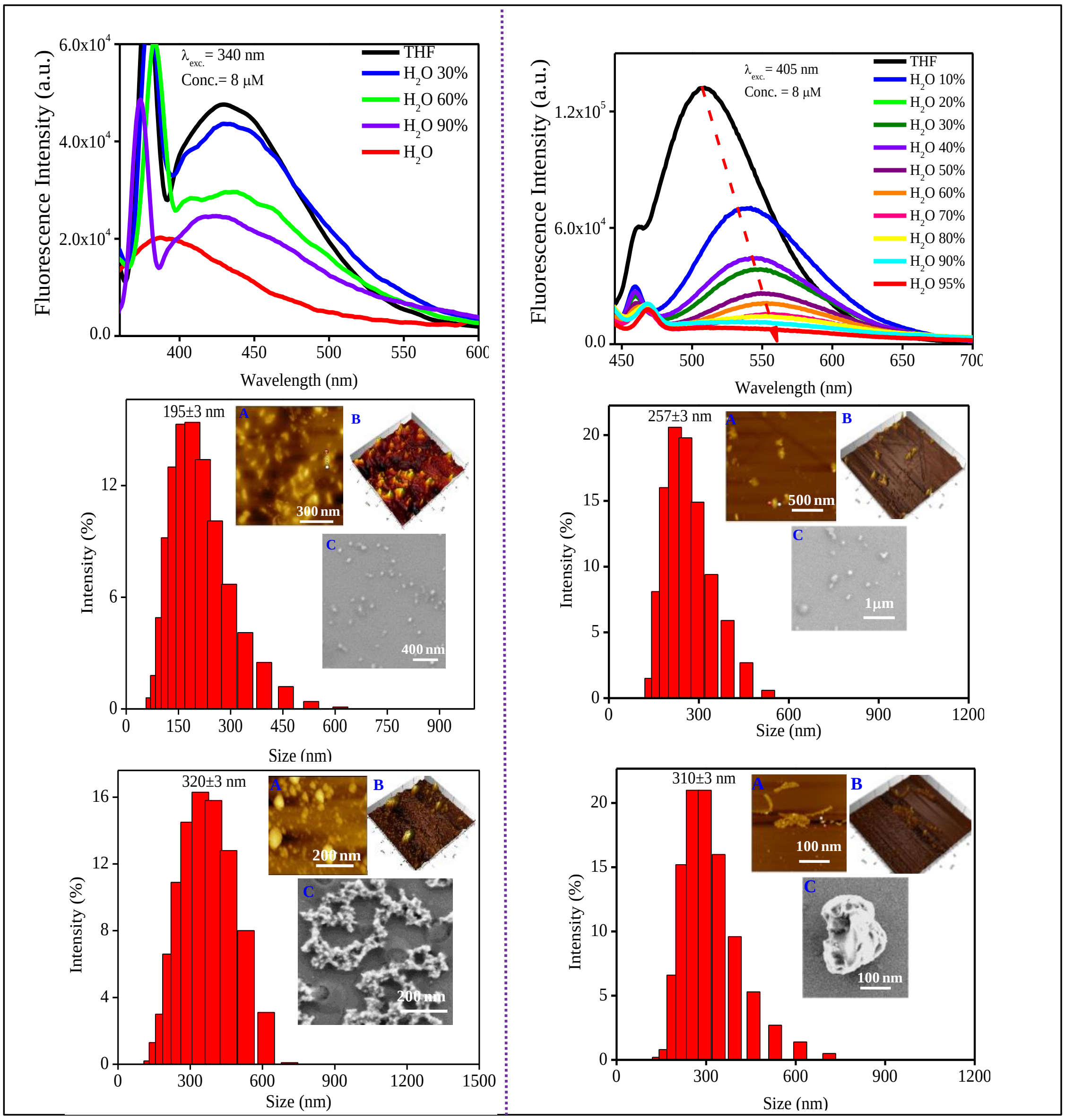

Figure S2: Aggregation caused quenching (ACQ) studies of parent PMI (left column) and DMAPMI (right column) luminogens. (Top row) steady-state emission spectra. The characterization (DLS, SEM and AFM) at low water (10%) content (middle row) and high water (90%) content (bottom row). Here, A,B,C represents AFM morphology, AFM 3D height profile, and SEM image respectively.

Crystallographic data

➢ **Parent PMI (3a):**

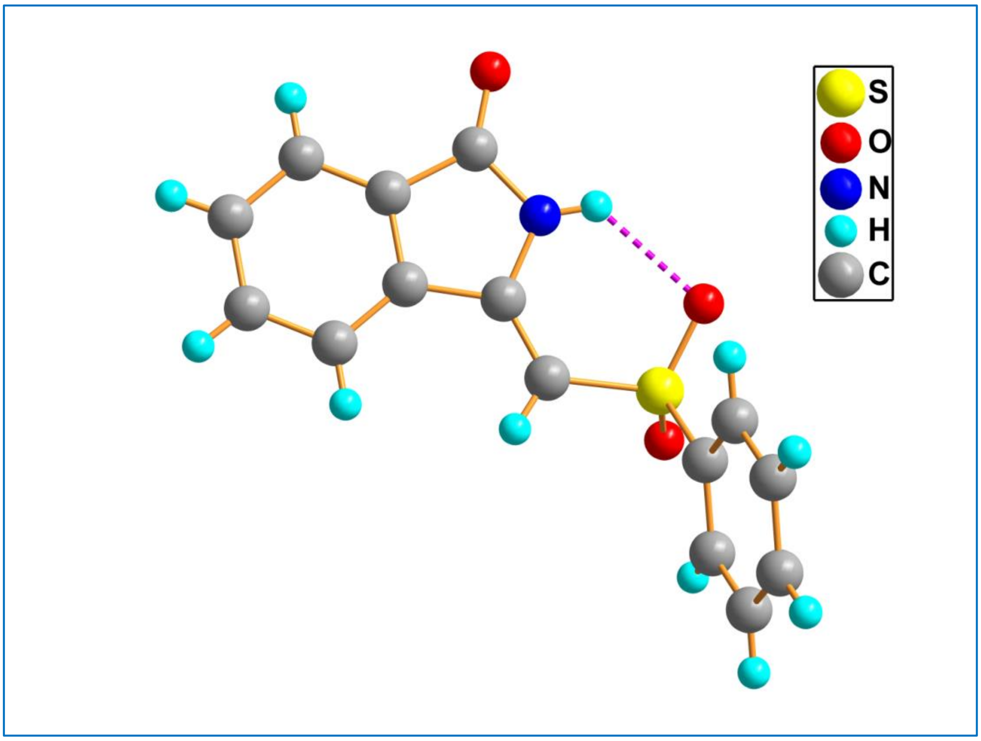

Table 1: Crystal data and structure refinement for CH-574_a

Identification code	CH-574_a	
CCDC	1572548	
Empirical formula	$C_{15} H_{11} N O_3 S$	
Formula weight	285.31	
Temperature	296(2) K	
Wavelength	0.71073 Å	
Crystal system	triclinic	
Space group	P -1	
Unit cell dimensions	a = 7.089(8)Å	α= 85.46(3)°.
	b = 7.256(8)Å	β= 77.90(3) °.
	c = 13.569(15)Å	γ =70.37(3) °.
Volume	642.8(12) $Å^3$	
Z	2	
Density (calculated)	1.474 Mg/m^3	
Absorption coefficient	0.258 mm^{-1}	
F(000)	296.0	
Theta ranges for data collection	1.535 to 28.525 °.	

Index ranges	-9<=h<=9, -5<=k<=9, -18<=l<=18
Reflections collected	11089
Independent reflections	3275 [R(int) = 0.1089]
Completeness to theta = 25.242 °	98.7 %
Refinement method	Full-matrix least-squares on F^2
Data / restraints / parameters	3233 / 0 / 181
Goodness-of-fit on F^2	0.812
Final R indices [I>2sigma(I)]	R1 = 0.0577, wR2 = 0.1364
R indices (all data)	R1 = 0.1190, wR2 = 0.1751
Extinction coefficient	n/a
Largest diff. peak and hole	0.453 and -0.633 e.Å^{-3}

- **Crystallographic Data of Compound DPAPMI (3b):**

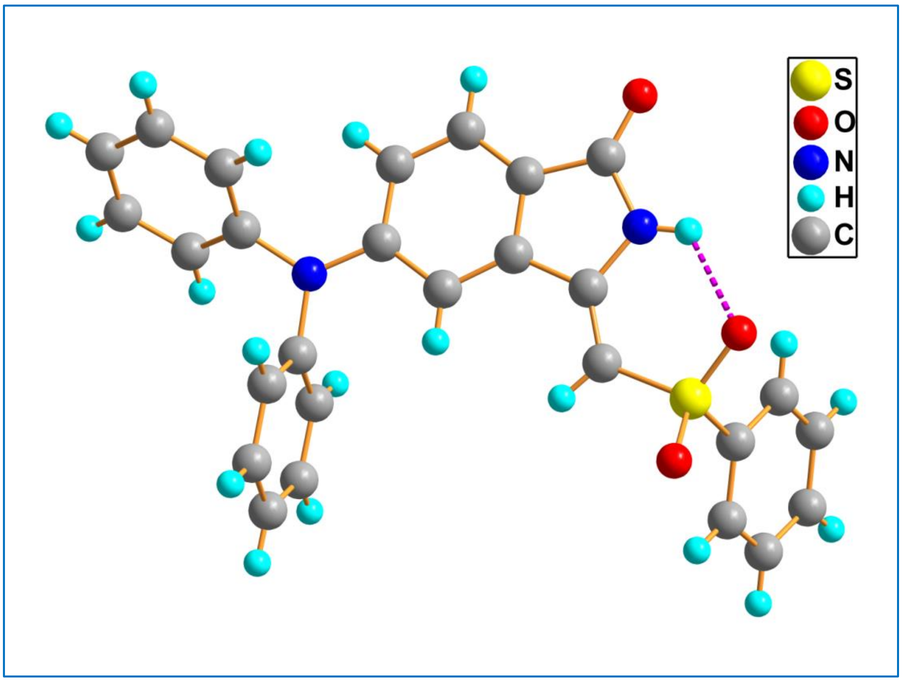

Table 1: Crystal data and structure refinement for CH-575_a

Identification code	CH-575_a
CCDC	1536146
Empirical formula	C_{27} H_{20} N_2 O_3 S
Formula weight	452.51
Temperature	293(2) K

Wavelength	0.71073 Å	
Crystal system	monoclinic	
Space group	P 21/c	
Unit cell dimensions	a = 12.6727(18)Å	α= 90°.
	b = 15.398 (3) Å	β= 110.633(6)°.
	c = 11.9061 (15) Å	γ = 90°.
Volume	2174.3(6) $Å^3$	
Z	4	
Density (calculated)	1.382 Mg/m^3	
Absorption coefficient	0.182 mm^{-1}	
F(000)	944.0	
Theta ranges for data collection	2.414 to 28.411°.	
Index ranges	-16<=h<=16, -20<=k<= 20, -15<=l<=15	
Reflections collected	69292	
Independent reflections	5445 [R(int) = 0.1526]	
Completeness to theta = 25.242°	99.6 %	
Refinement method	Full-matrix least-squares on F^2	
Data / restraints / parameters	5423 / 0 / 298	
Goodness-of-fit on F^2	0.917	
Final R indices [I>2sigma(I)]	R1 = 0.0508, wR2 = 0.1309	
R indices (all data)	R1 = 0.0929, wR2 = 0.1574	
Extinction coefficient	n/a	
Largest diff. peak and hole	0.297 and -0.452 e.$Å^{-3}$	

➢ **Crystallographic Data of Compound CPMI (3c):**

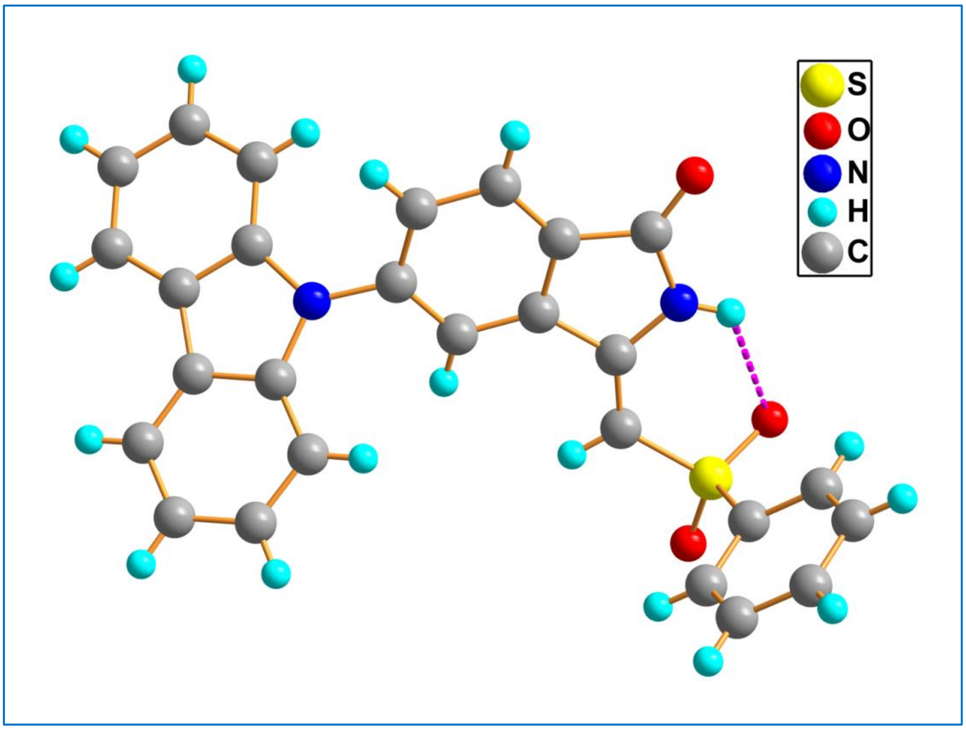

Table 1: Crystal data and structure refinement for CH-576_a

Identification code	CH-576_a	
CCDC	1571721	
Empirical formula	$C_{27} H_{18} N_2 O_3 S$	
Formula weight	450.49	
Temperature	296(2) K	
Wavelength	0.71073 Å	
Crystal system	monoclinic	
Space group	C 2	
Unit cell dimensions	a = 25.719(7) Å	α= 90 °.
	b = 7.5368(18) Å	β= 116.390(10) °.
	c = 11.759(3) Å	γ = 90°.
Volume	2041.8(9) $Å^3$	
Z	4	
Density (calculated)	1.465 Mg/m^3	
Absorption coefficient	0.194 mm^{-1}	
F(000)	936.0	
Theta ranges for data collection	2.84 to 24.41 °.	
Index ranges	-34 <=h<=34, -10 <=k<=9, -15 <=l<=15	
Reflections collected	47733	

Independent reflections	5174
Completeness to theta = 25.242°	98.5 %
Refinement method	Full-matrix least-squares on F^2
Data / restraints / parameters	5062 /1 / 298
Goodness-of-fit on F^2	0.887
Final R indices [I>2sigma(I)]	R1 = 0.0521, wR2 = 0.1205
R indices (all data)	R1 = 0.1027, wR2 = 0.1454
Extinction coefficient	n/a
Largest diff. peak and hole	0.275 and -0.354 e.Å^{-3}

- **Crystallographic Data of Compound DMAPMI (3d):**

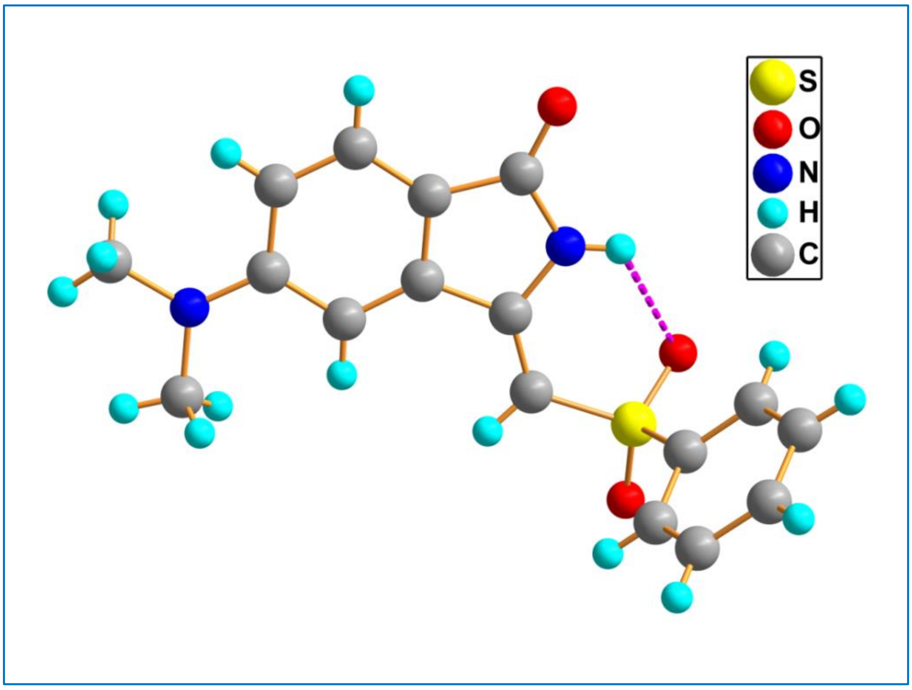

Table 1: Crystal data and structure refinement for CH_A_577_0m_a

CCDC	1572060
Identification code	CH_A_577_0m_a
Empirical formula	$C_{17}H_{16}N_2O_3S$
Formula weight	328.38
Temperature	296(2) K
Wavelength	0.71073 Å
Crystal system	monoclinic
Space group	P 21/n

Unit cell dimensions	a = 8.172(3) Å	α= 90 °.
	b = 9.666(3) Å	β= 99.512(11)°.
	c = 20.305(7) Å	γ = 90°.
Volume	1581.9(9) Å^3	
Z	4	
Density (calculated)	1.379 Mg/m^3	
Absorption coefficient	0.221mm^{-1}	
F(000)	688.0	
Theta ranges for data collection	2.34 to 24.66 °.	
Index ranges	-9<=h<=9, -11<=k<=11, -24<=l<=24	
Reflections collected	50889	
Independent reflections	2859	
Completeness to theta = 25.242°	100 %	
Refinement method	Full-matrix least-squares on F^2	
Data / restraints / parameters	2860 /0 / 210	
Goodness-of-fit on F^2	1.015	
Final R indices [I>2sigma(I)]	R1 = 0.0469, wR2 = 0.0762	
R indices (all data)	R1 = 0.1295, wR2 = 0.1534	
Extinction coefficient	n/a	
Largest diff. peak and hole	0.249 and -0.389 e.Å^{-3}	

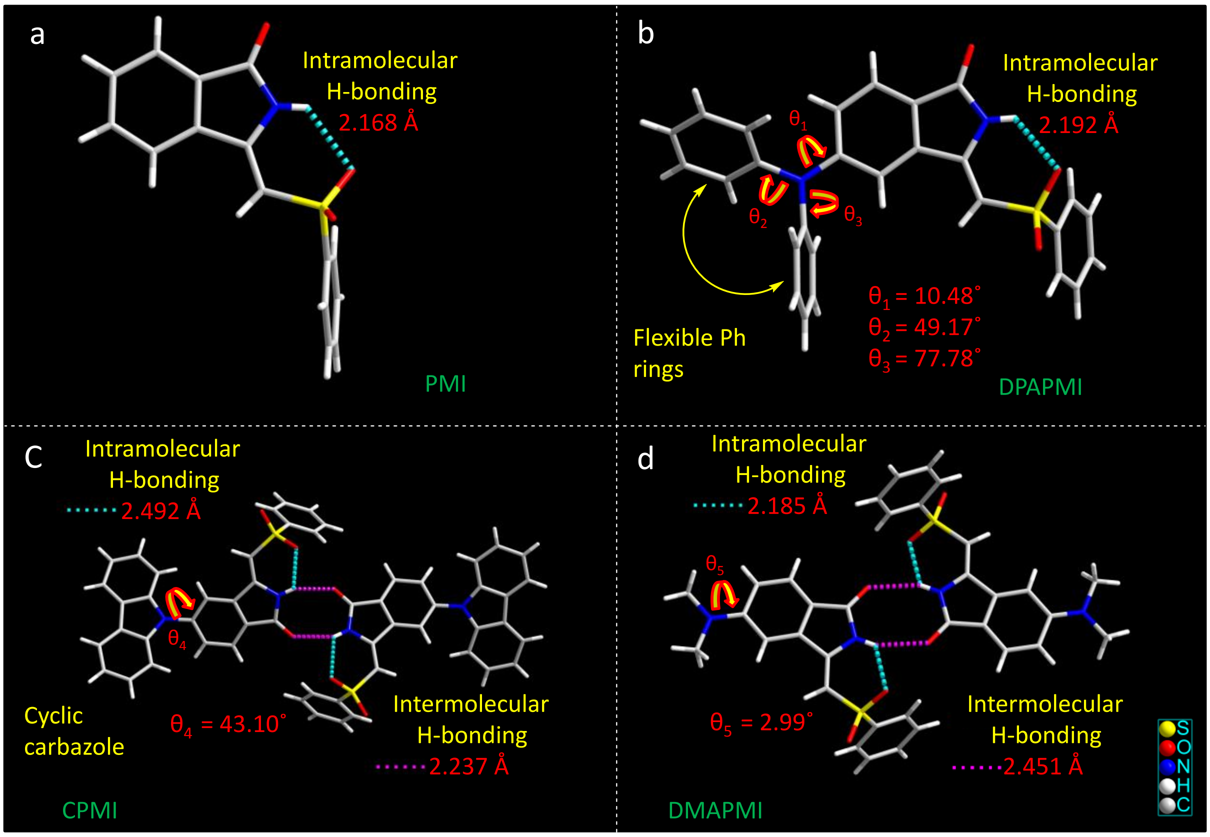

Figure S3 (A): Representation of intramolecular H-bond and angle between donor and acceptor moiety of PMI and its donor substituted derivatives. For better clarification of angle between donor and acceptor we have provided separate image provided in Figure S3 (B).

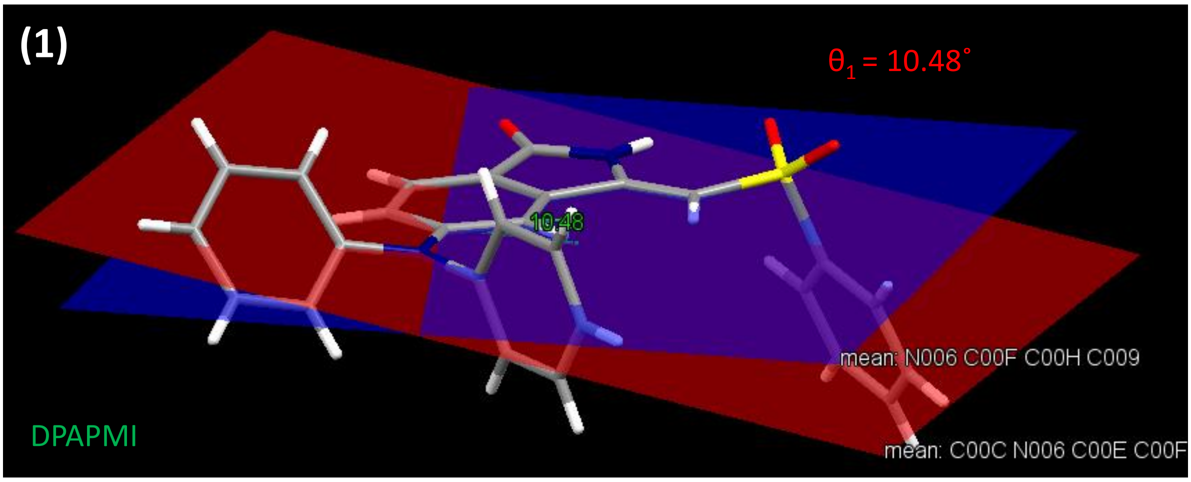
(1)
θ1 = 10.48˚
10.48
mean: N006 C00F C00H C009
mean: C00C N006 C00E C00F
DPAPMI

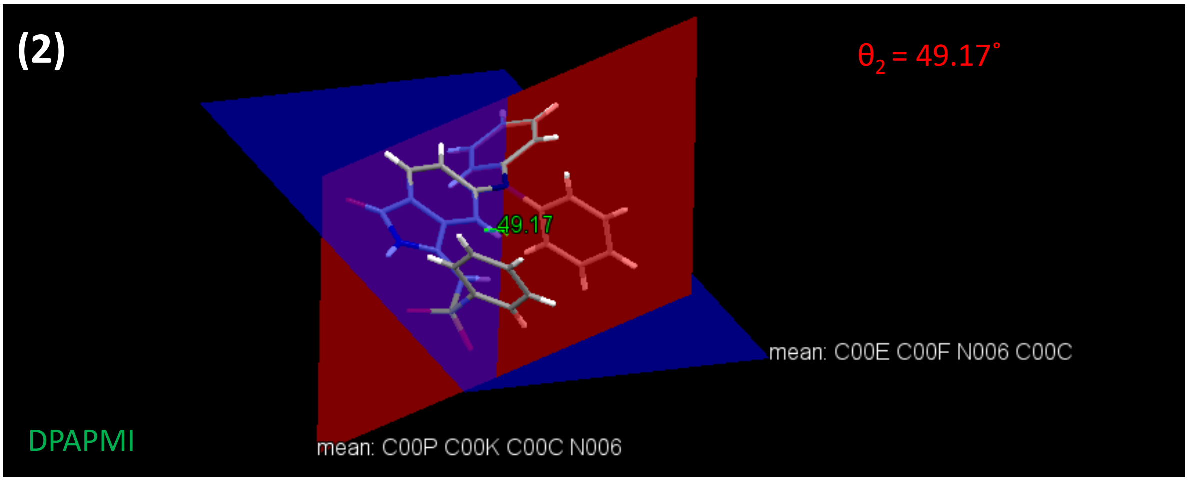
(2)
θ2 = 49.17˚
49.17
mean: C00E C00F N006 C00C
mean: C00P C00K C00C N006
DPAPMI

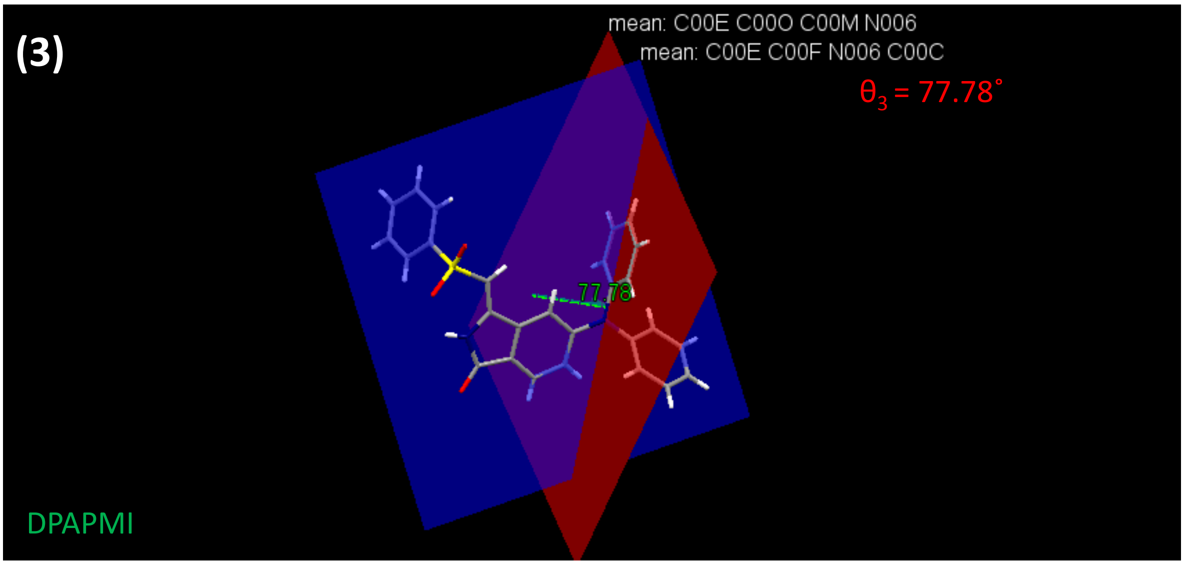
(3)
mean: C00E C00O C00M N006
mean: C00E C00F N006 C00C
θ3 = 77.78˚
77.78
DPAPMI

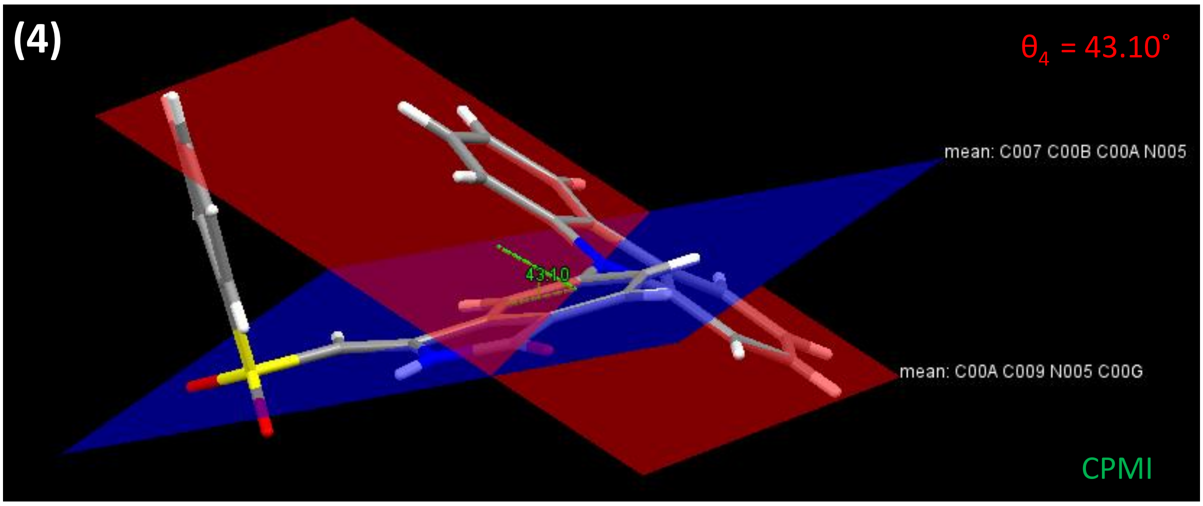

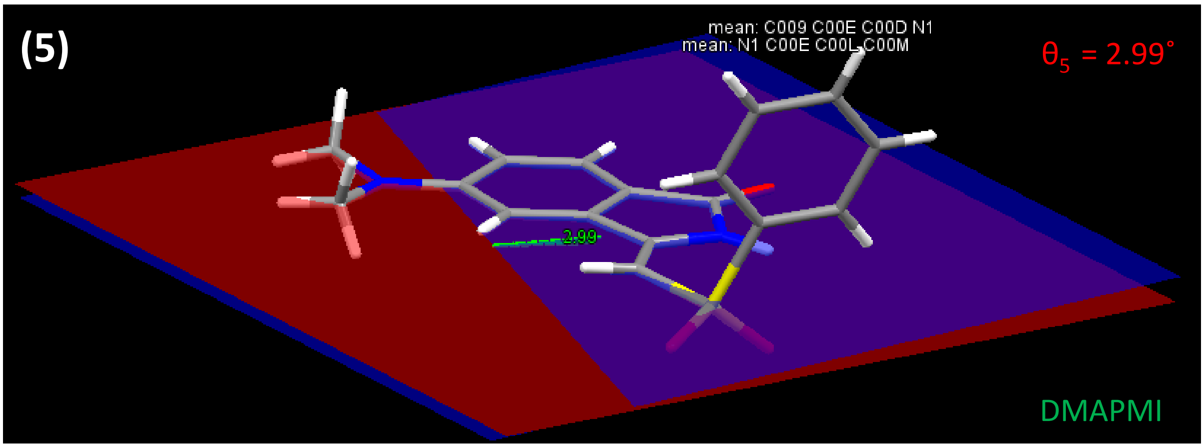

Figure S3 (B): Representation of different angles of interest for each luminogen. (1) Angle between donor DPA unit (red plane) and acceptor PMI (blue plane) in DPAPMI luminogen. (2) Twisted angle of 1st phenyl ring (θ_2) (red plane) with PMI core (blue plane) in DPAPMI. (3) Twisted angle of 2nd phenyl ring (θ_3) (red plane) with PMI core (blue plane) in DPAPMI. (4) Twisted angle of carbazole donor (red plane) with central acceptor (PMI) core (blue plane) in CPMI luminogen. (5) Twisted angle of DMA donor (red plane) with PMI core (blue plane) in DMAPMI luminogen.

Note S2: Hirshfeld Surface Analysis

The Hirshfeld surface analysis is an effective way to decipher the intermolecular interactions in the solid state. Interactions to be mapped on the Hirshfeld surface are d_e and d_i, the distances of an atom external or internal to the generated Hirshfeld surface.[3-5, 6] Together these two values results in a pair (d_e and d_i), and generates the 2D finger print plot, where the different colors on the finger print plot represent the frequency of occurrence of interaction. When two Hirshfeld surfaces of neighboring molecule touch, it will display by a red spot identical in color intensity with equal size and shape. The normalized contact distance (d_{norm}) constructed over Hirshfeld surface based on de, d_i and the van der Waals radii of the molecule.[3-5] The d_{norm} values are mapped onto the Hirshfeld surface by using a red, white and blue color scheme; where red, white and blue regions corresponding to the strong, medium and weak interactions respectively.[3-5, 6] Moreover, all the Hirshfeld surfaces were generated using Crystal Explorer 3.1 software with iso-value of 0.5 au. Notably, the void spaces were calculated using Crystal Explorer 3.1 software with the iso-value of 0.002 au.

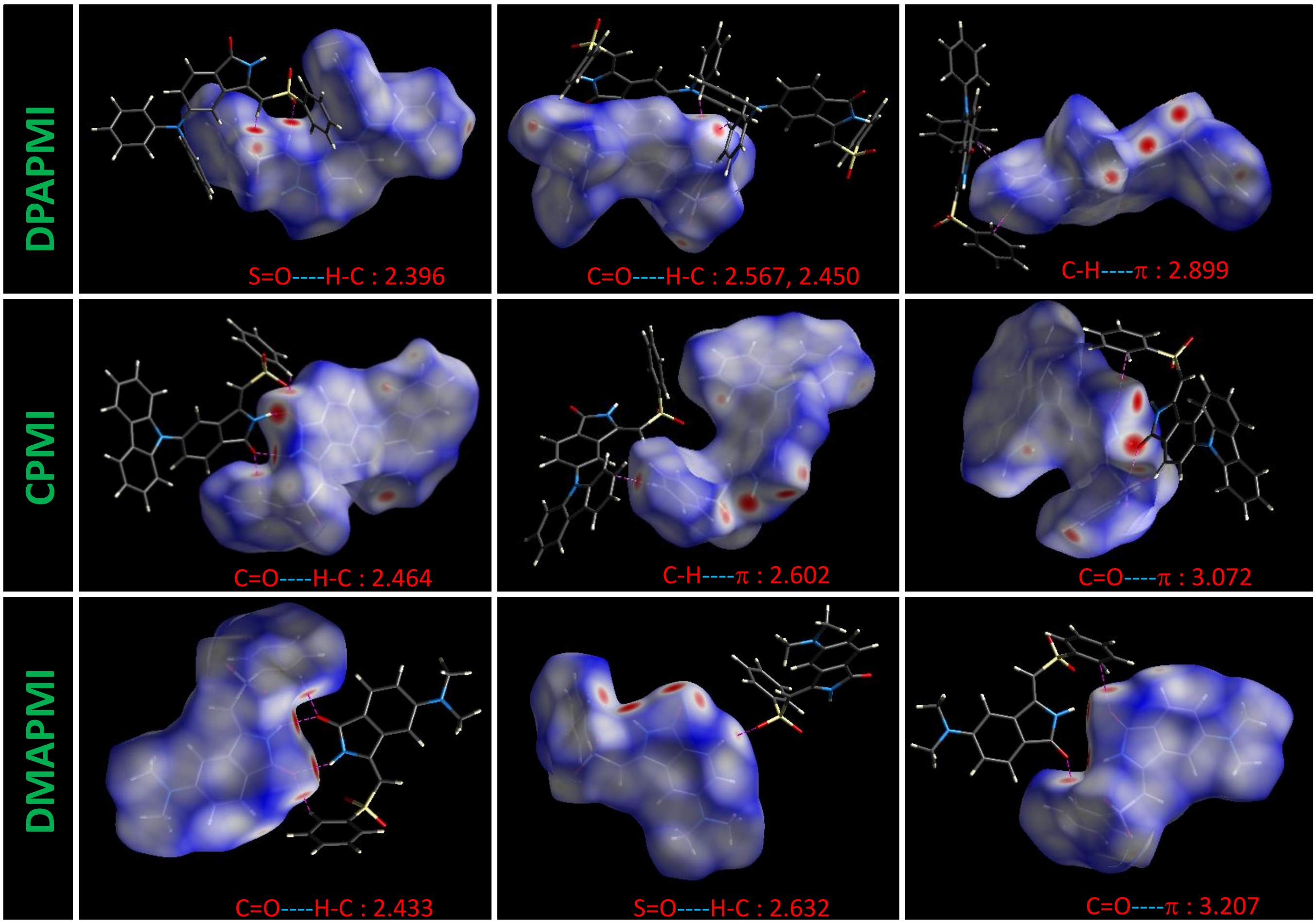

Figure S4(A): The d_{norm} mapped over the Hirshfeld surface using universal red, blue and white color code to show important interactions taking a neighbouring molecule. The red, white, blue and color code indicates strong, medium and weak interactions respectively

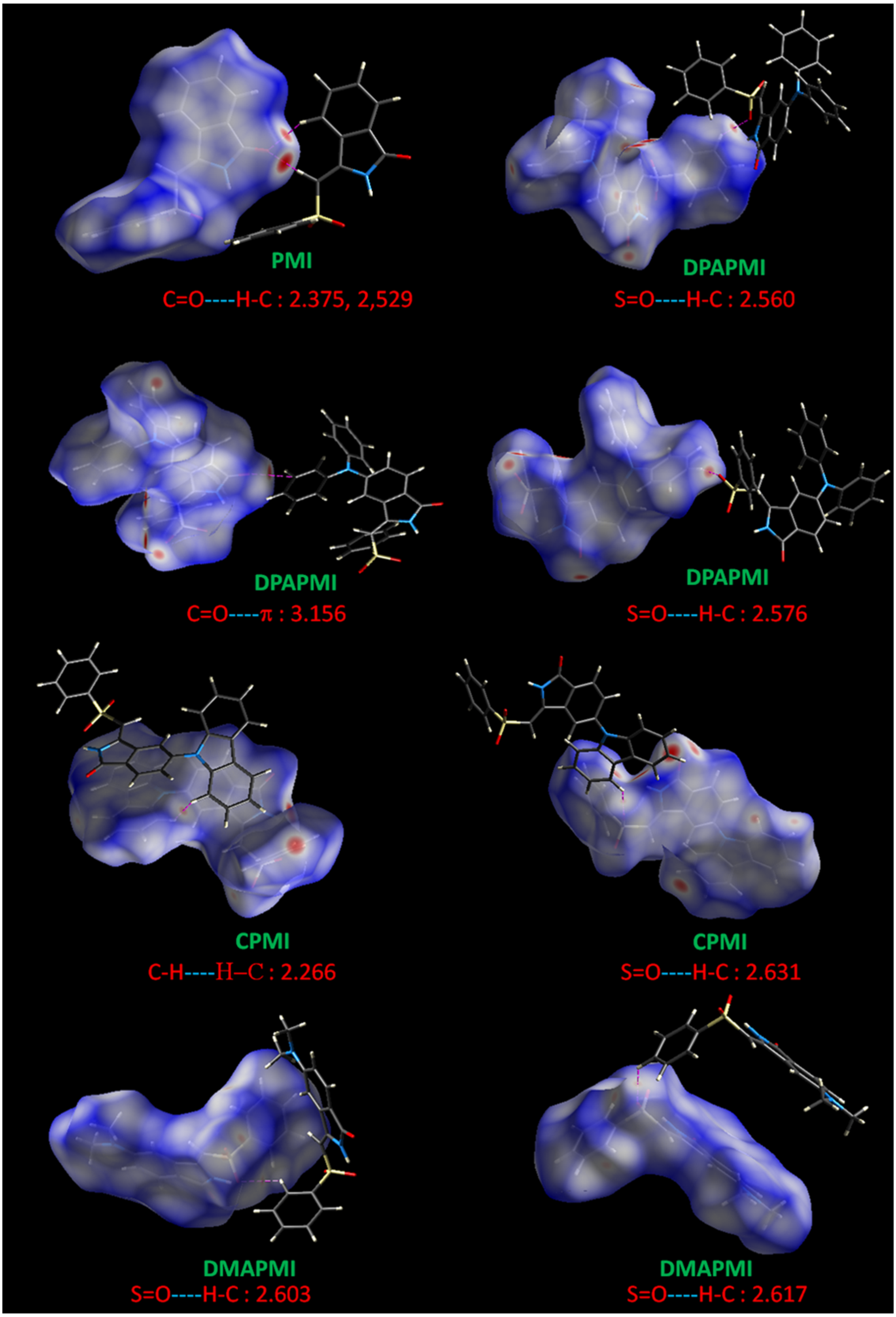

Figure S4(B): d_{norm} mapped over the Hirshfeld surface using universal red, white and blue color code, which indicates strong, medium and weak interactions, respectively (for details see note S2).

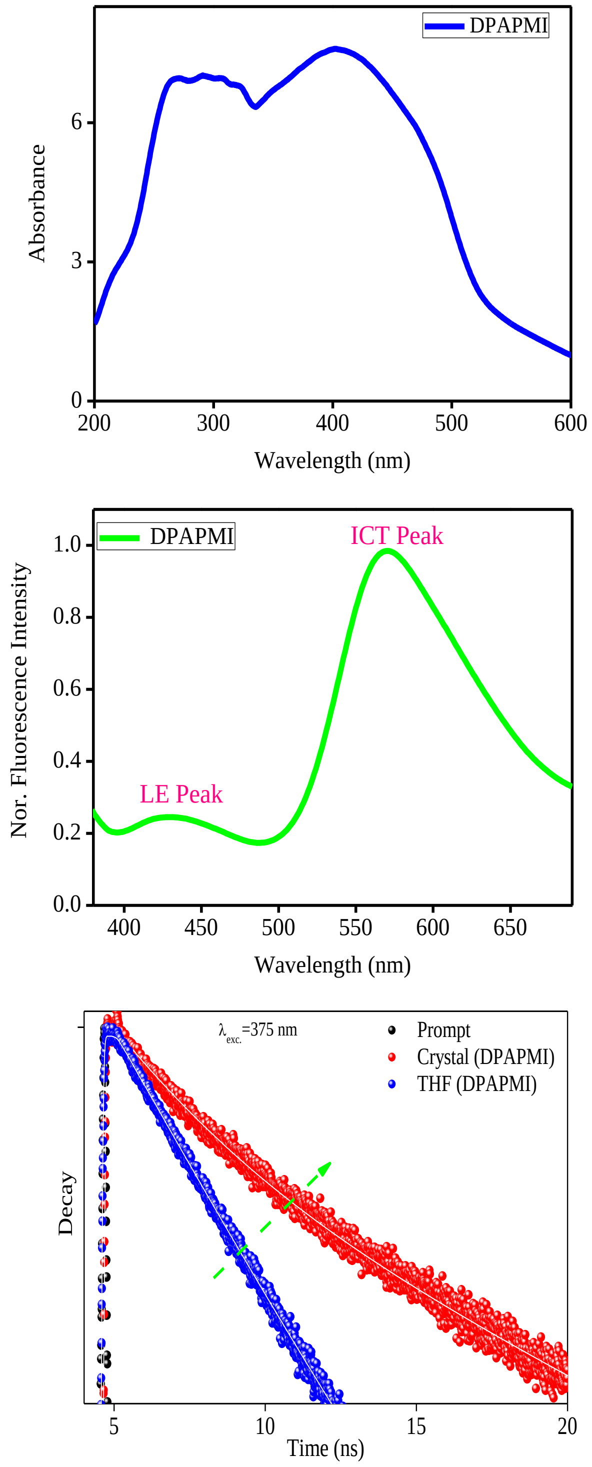

Figure S5: Absorption (top), emission spectra (middle) and time-resolved decay (bottom) of the crystal of DPAPMI molecule. For comparison purpose, the time-resolved decay in THF solvent also provided. Excitation wavelength used for emission and time-resolved data are, $\lambda_{ex.}$ = 350 nm and $\lambda_{ex.}$ = 375 nm, respectively.

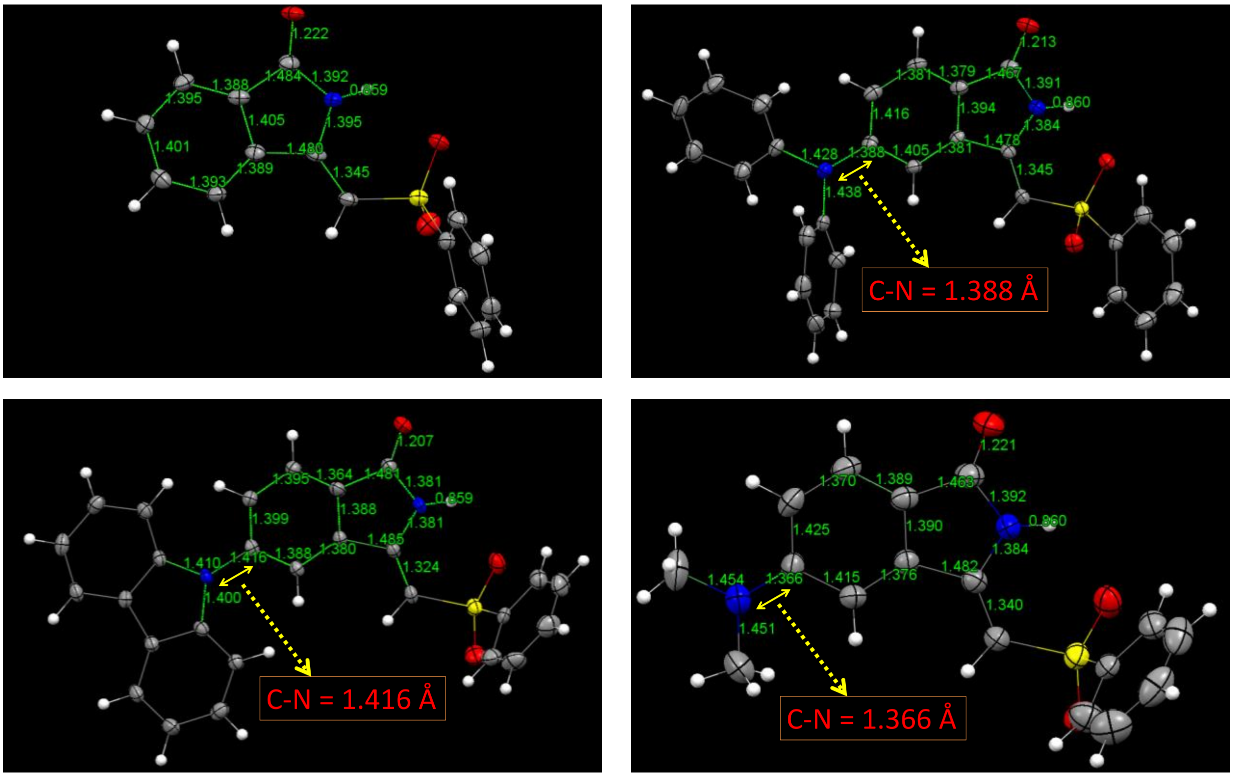

Figure S6: Comparison of bond distances between PMI (top left) and its donor substituted analogues DPAPMI, (top right), CPMI (bottom left) and DMAPMI (bottom right). The calculated C-N bond distances between donor and acceptor shows decreasing trend of CPMI>DPAPMI>DMAPMI shown by yellow arrow. Lowest D-A bond distance (1.36 Å) in DMAPMI indicates the highest charge transfer ability among all.

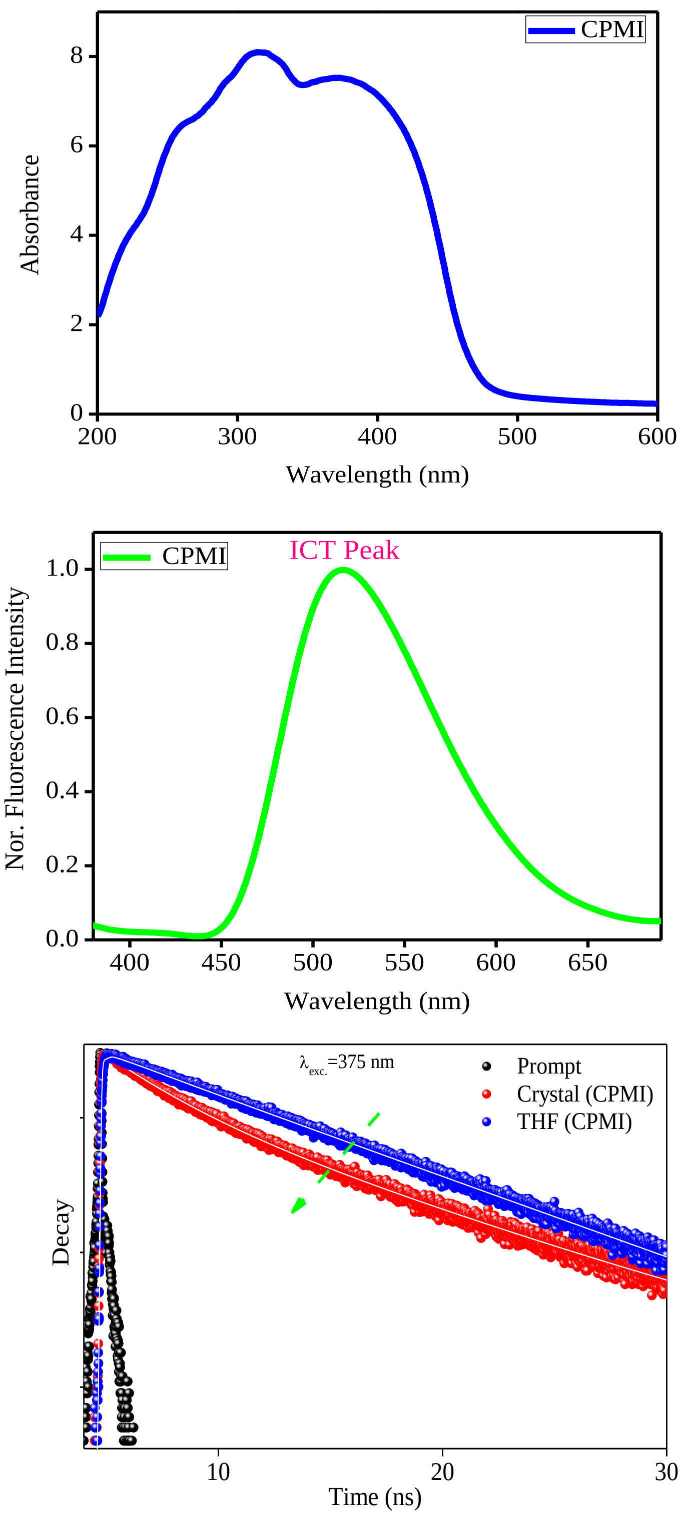

Figure S7: Absorption (top), emission spectra (middle) and time-resolved decay (bottom) of the crystal of CPMI molecule. For comparison purpose, the time-resolved decay in THF solvent also provided. Excitation wavelength used for emission and time-resolved data are, $\lambda_{ex.}$ = 350 nm and $\lambda_{ex.}$ = 375 nm respectively.

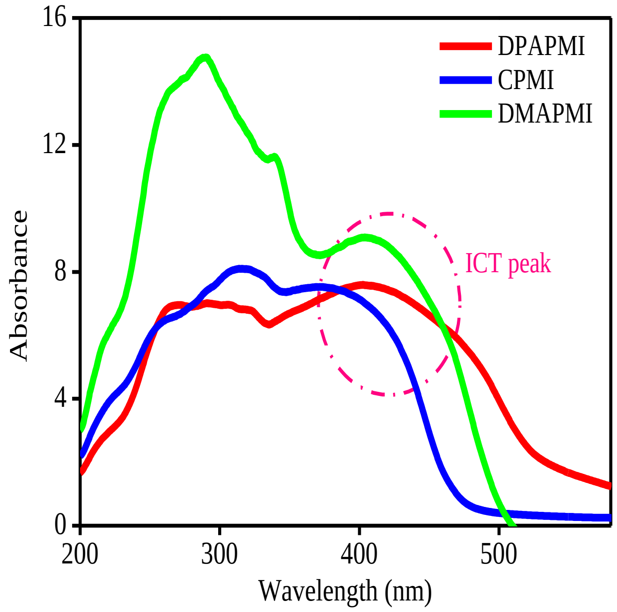

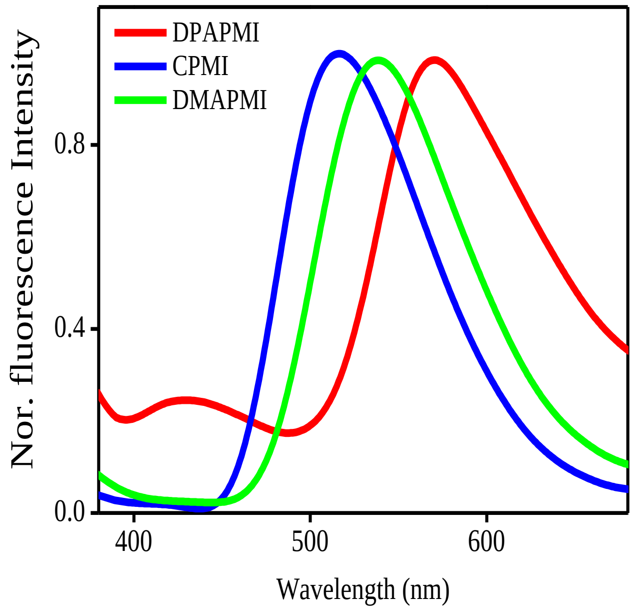

Figure S8: Comparison of absorption (top) and emission spectra (bottom) of the crystals of donor substituted DPAPMI, CPMI and DMAPMI luminogens.

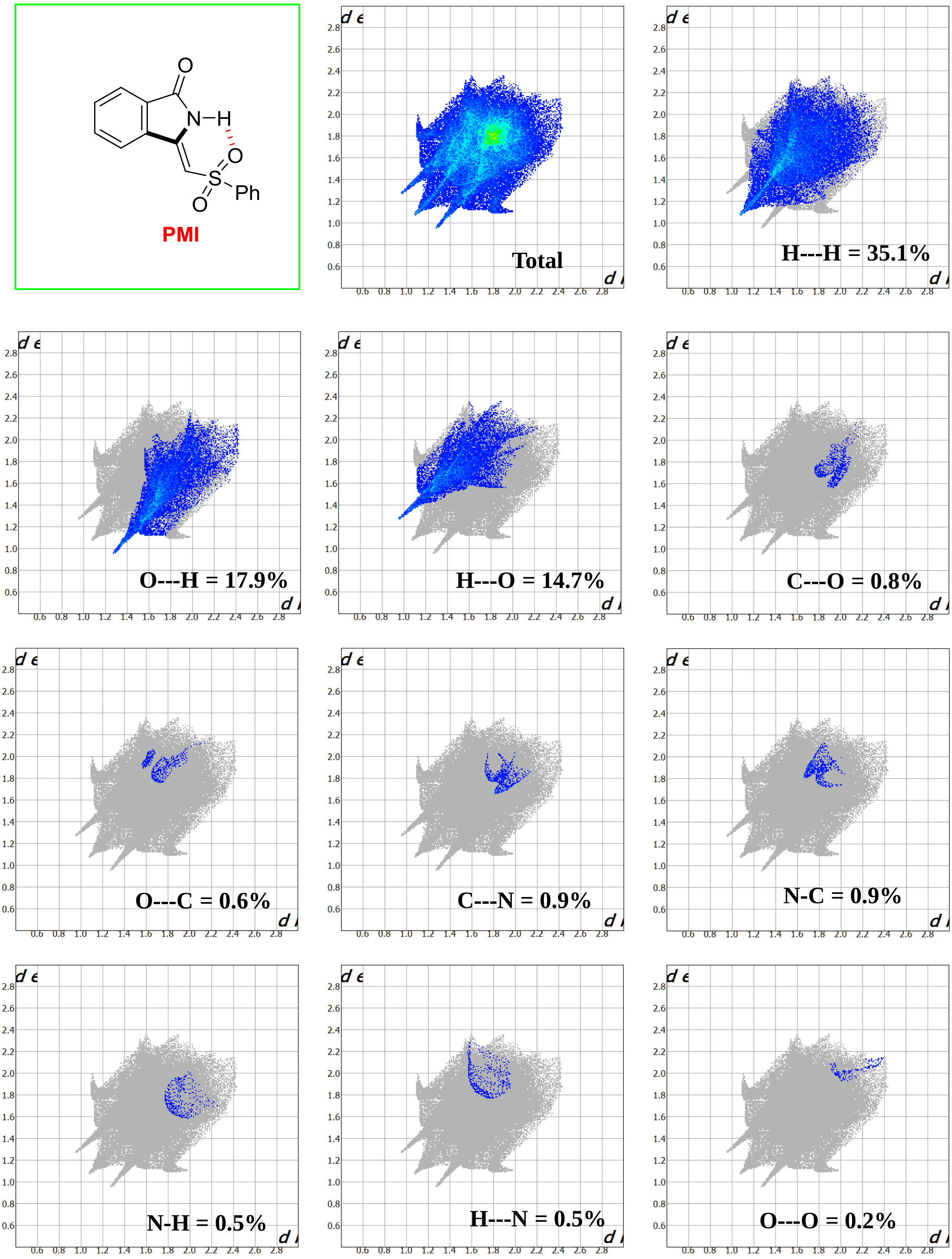

Figure S9: 2D fingerprint plots of parent PMI to obtain quantitative non-covalent interactions outlined from Hirshfeld surface analysis (Except C-H and C-C, all other interactions are shown here).

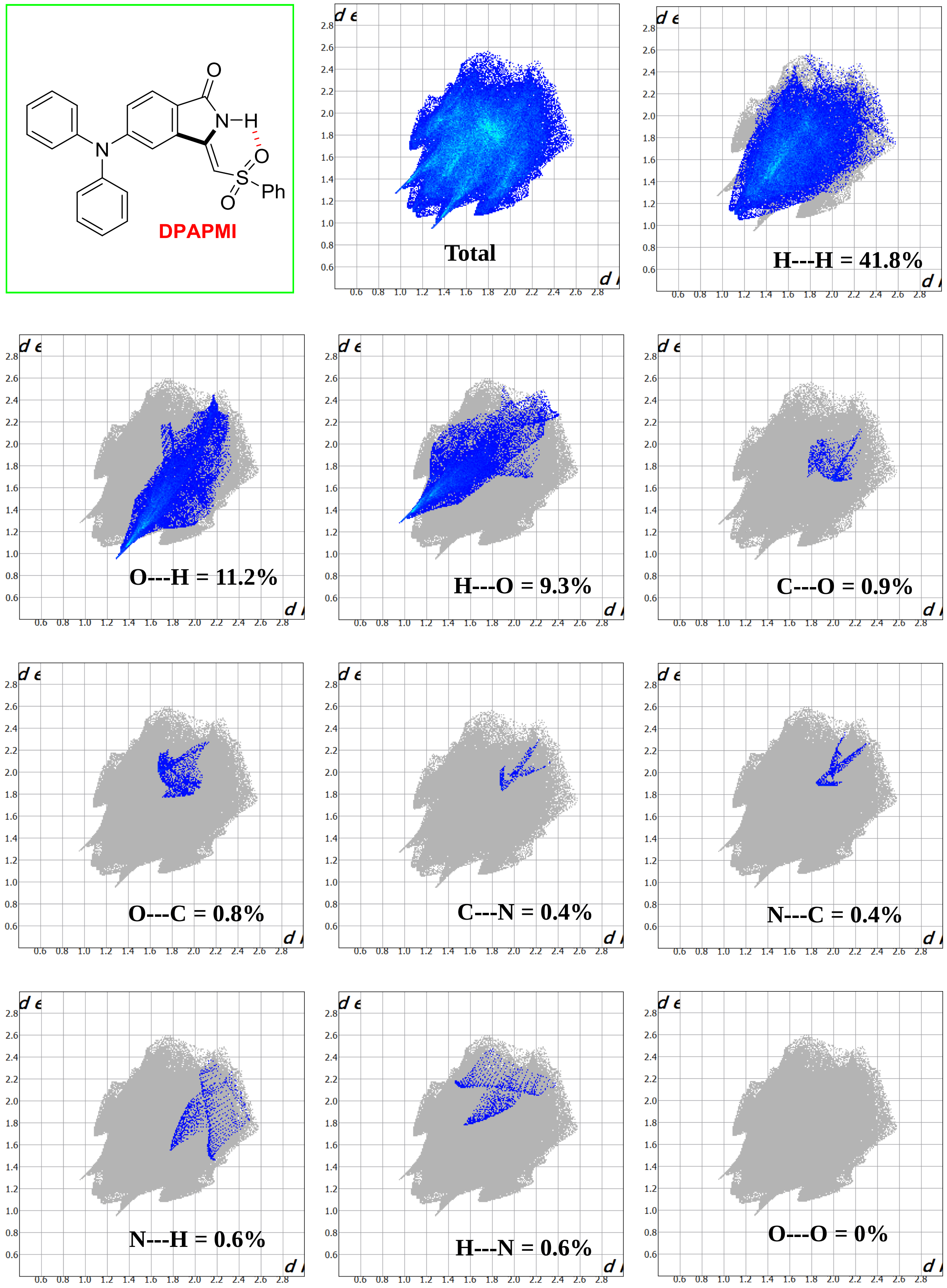

Figure S10: 2D fingerprint plots of DPAPMI luminogen to obtain quantitative non-covalent interactions outlined from Hirshfeld surface analysis (Except C-H and C-C, all other interactions are shown here).

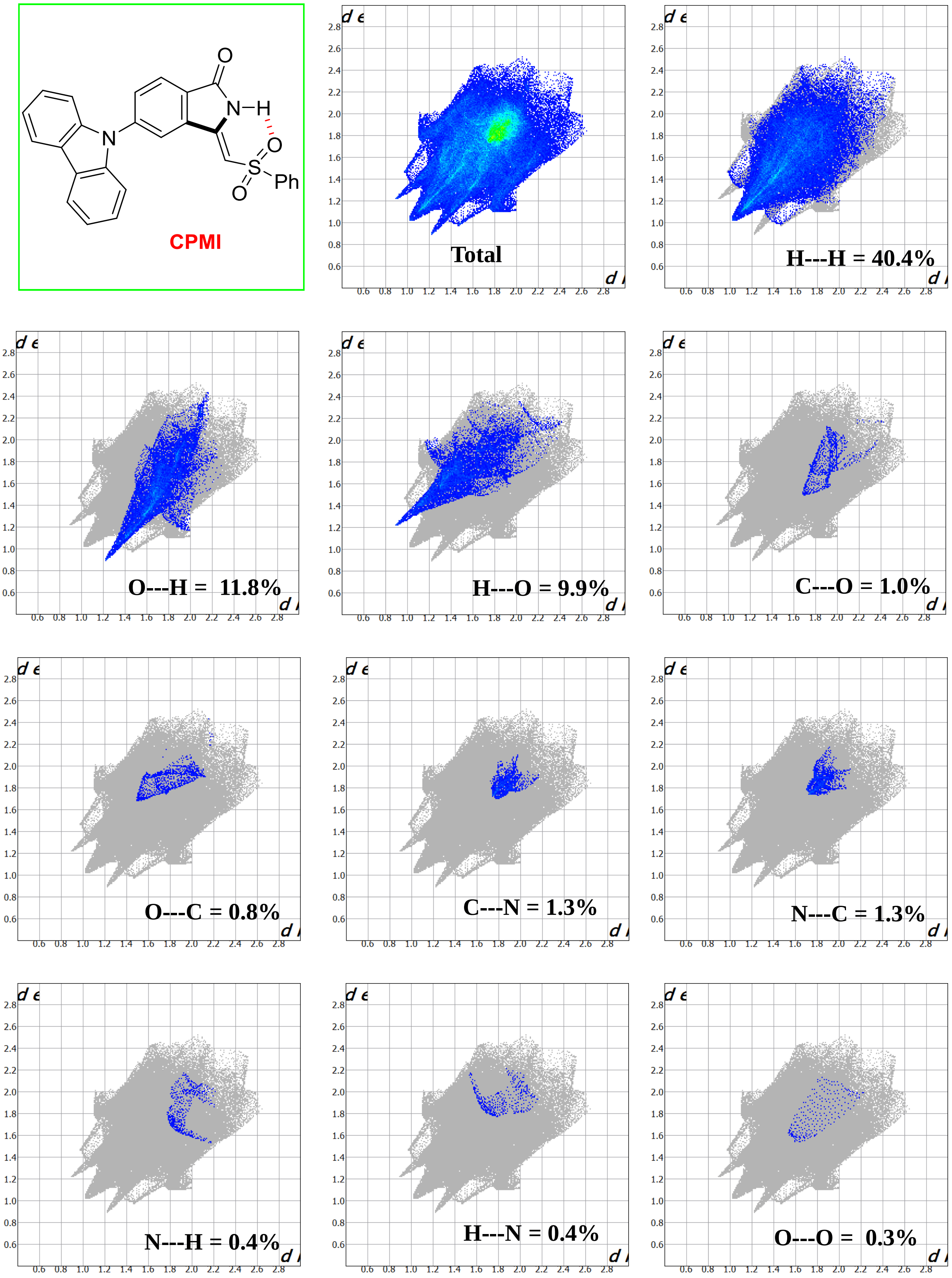

Figure S11: 2D fingerprint plots of CPMI luminogen to obtain quantitative non-covalent interactions outlined from Hirshfeld surface analysis (Except C-H and C-C, all other interactions are shown here).

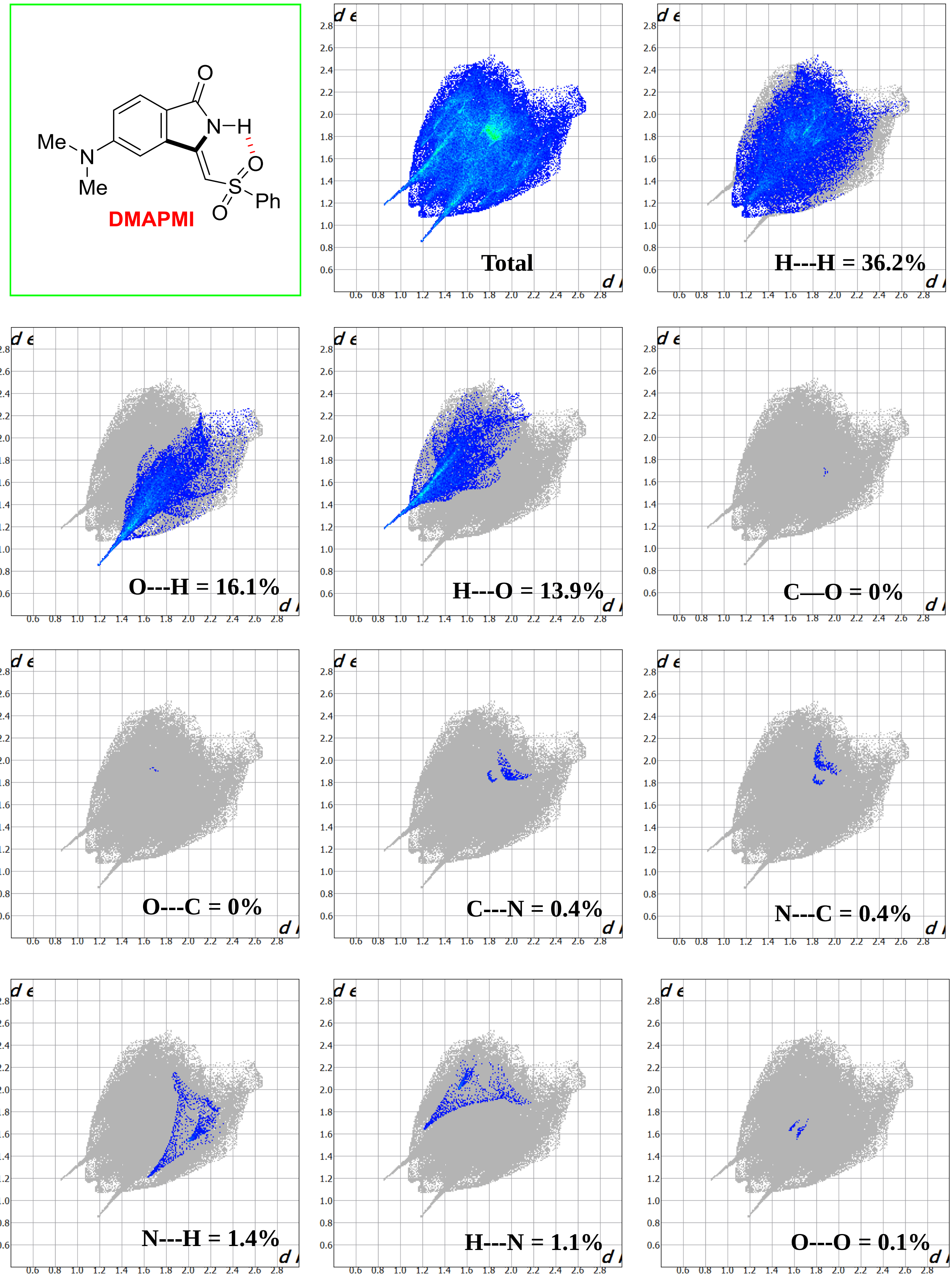

Figure S12: 2D fingerprint plots of DMAPMI luminogen to obtain quantitative non-covalent interactions outlined from Hirshfeld surface analysis (Except C-H and C-C, all other interactions are shown here).

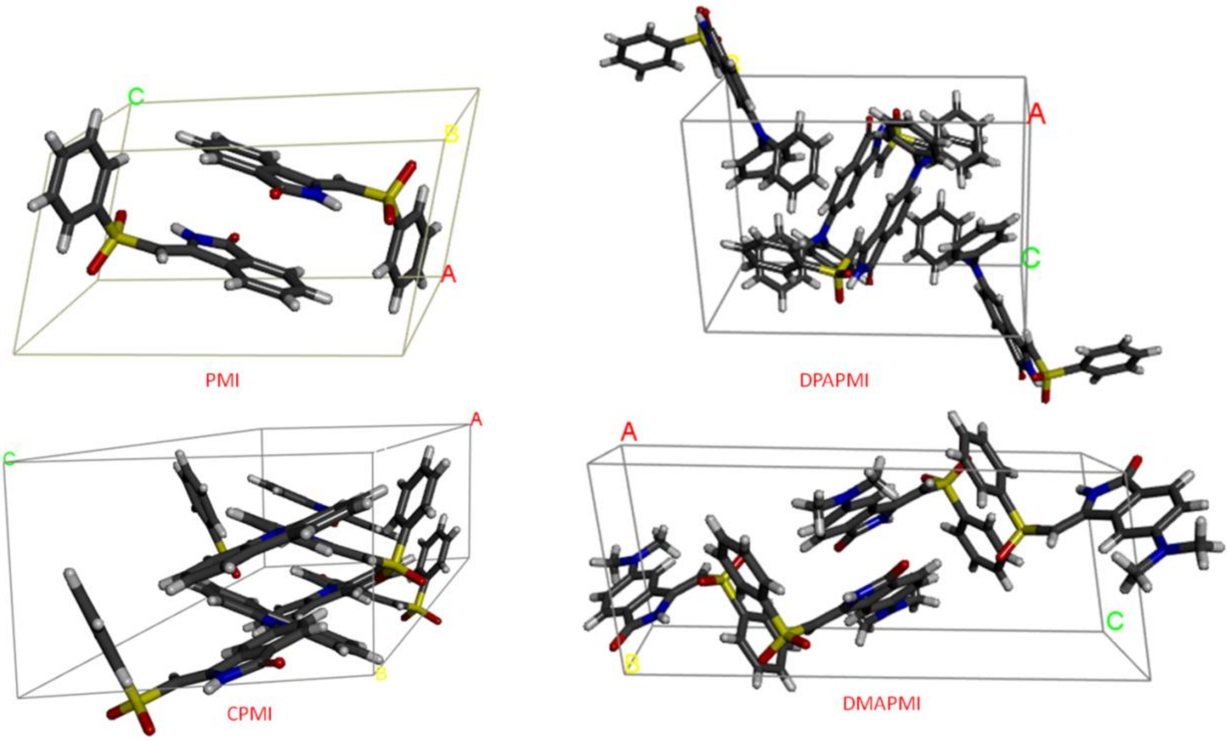

Figure S13: The unit cell of PMI, DPAPMI, CPMI and DMAPMI luminogens. Except CPMI, unit cell of luminogen contains two molecules per unit cell. Only, CPMI contains four molecules per unit cell, which subsequently minimizes the accessible void space.

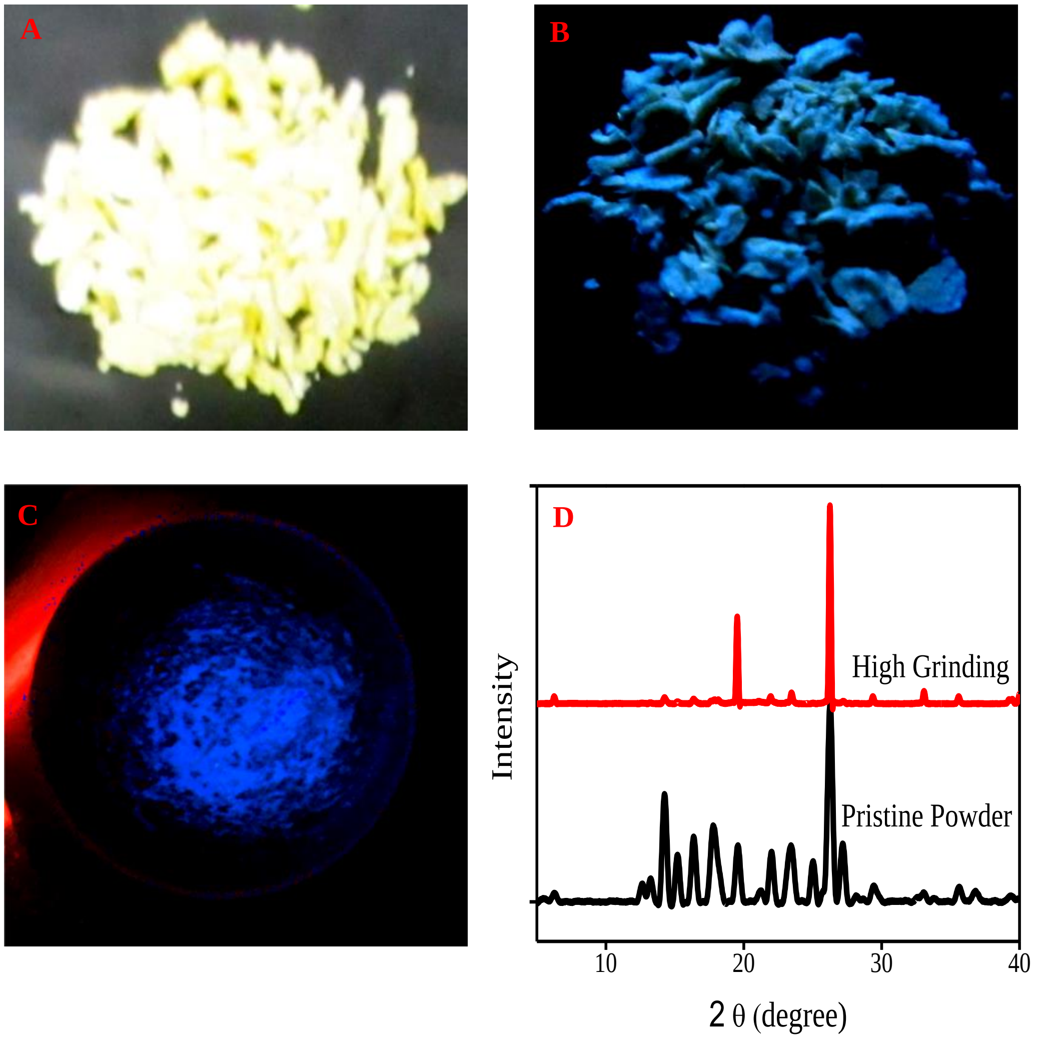

Figure S14: Pristine powder of PMI molecule under daylight (A), under UV (365 nm) light (B), under UV light after strong grinding (C). The PXRD patterns of pristine powder and grinded powder of PMI (D). The PXRD patterns before and after grinding shows crystalline features, suggesting that no phase change occurs due to mechanical force. The blue colour under UV light is the color of UV exposure, not the color of compound, which we have confirmed by taking the image under 265 nm UV light.

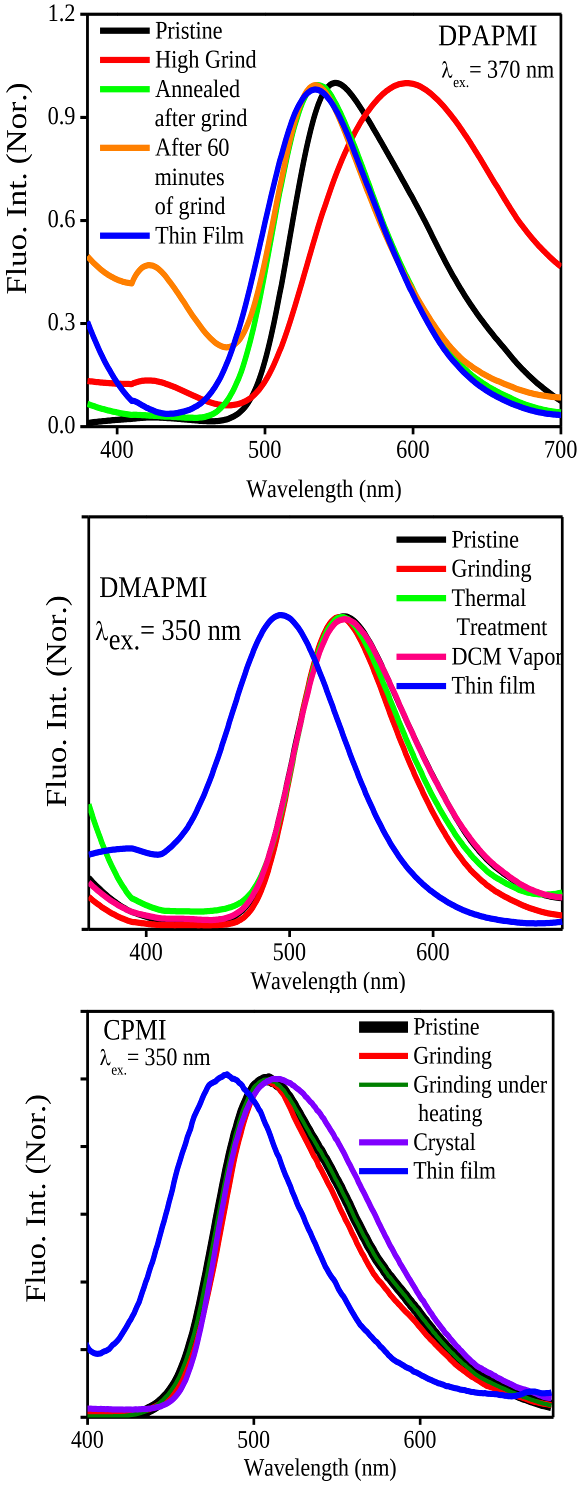

Figure S15: Emission profile of luminogens under different mechanical treatment Legends are given at the bottom-right side. Legends are given at the bottom-right side of the figure.

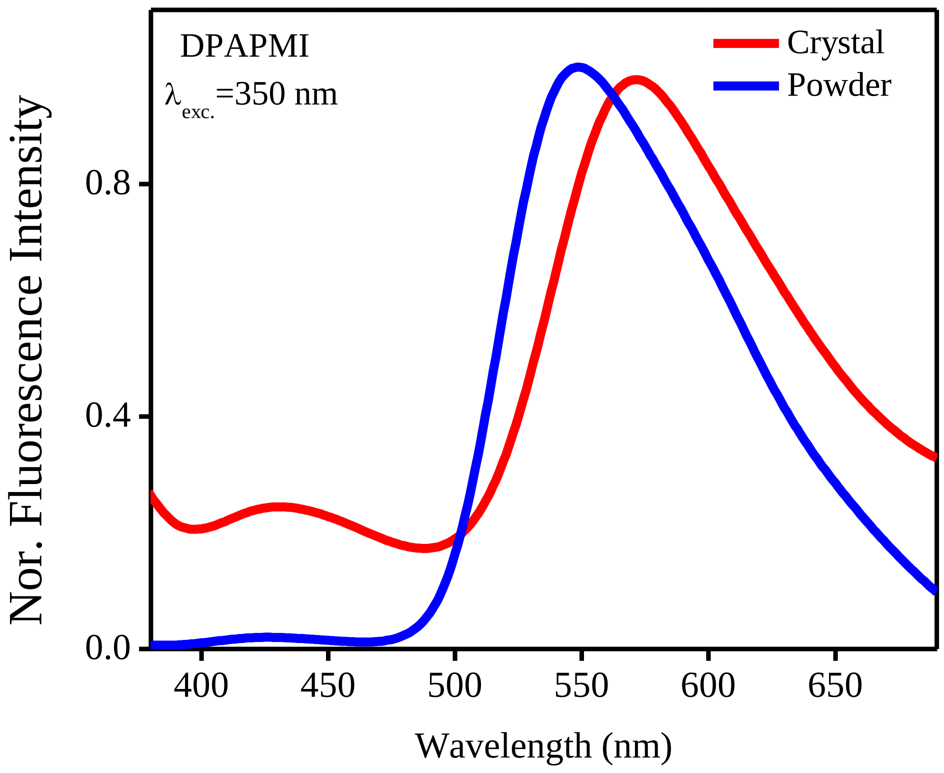

Figure S16: Emission spectra of DPAPMI crystal and powder forms. It is clear that lower energy CT peak in crystalline state ~25 nm is red-shifted compared to pristine powder, while higher energy LE peak position remains nearly unaltered

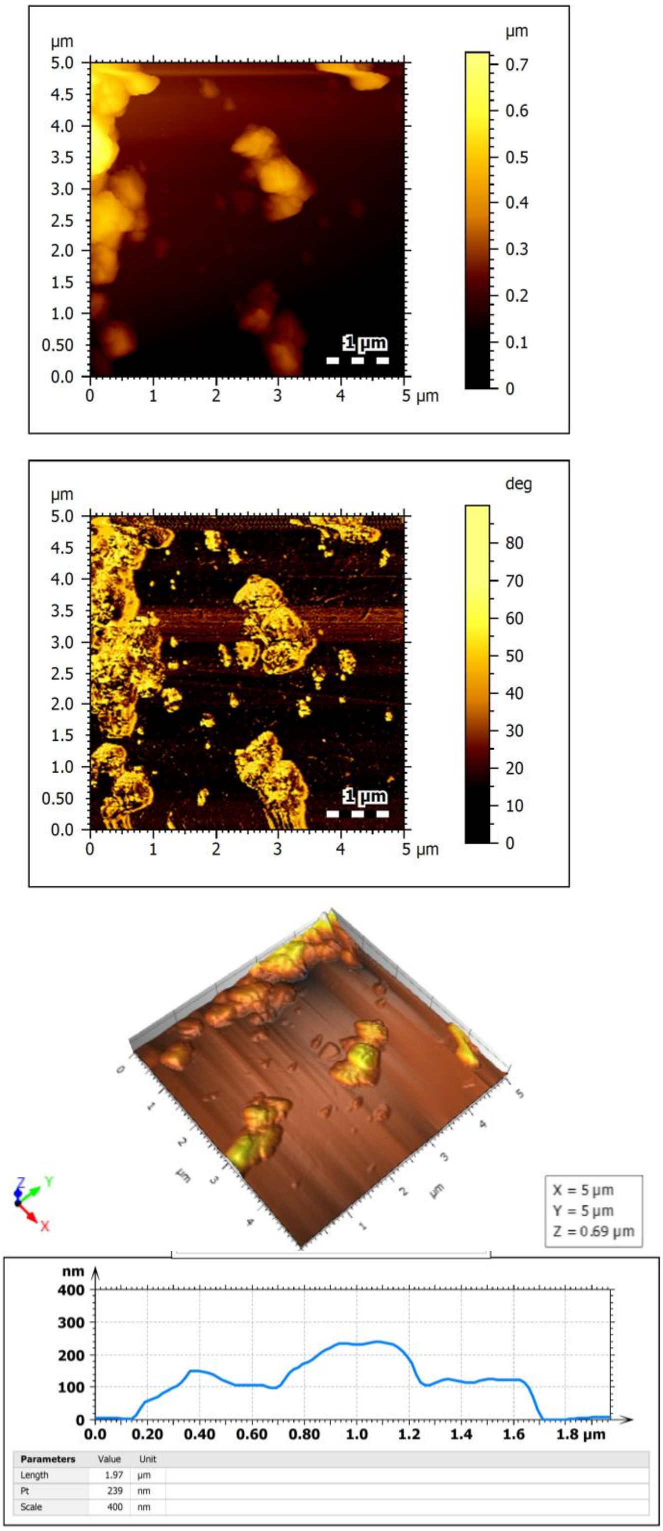

Figure S17. The tapping mode AFM images of DPAPMI pristine powder taken as film on quartz slide. From top to bottom represents height profile, morphology, 3D height profile and scale bar showing height (239 nm) image. This image shows the large aggregated morphology in the pristine state of DPAPMI luminogen before grinding.

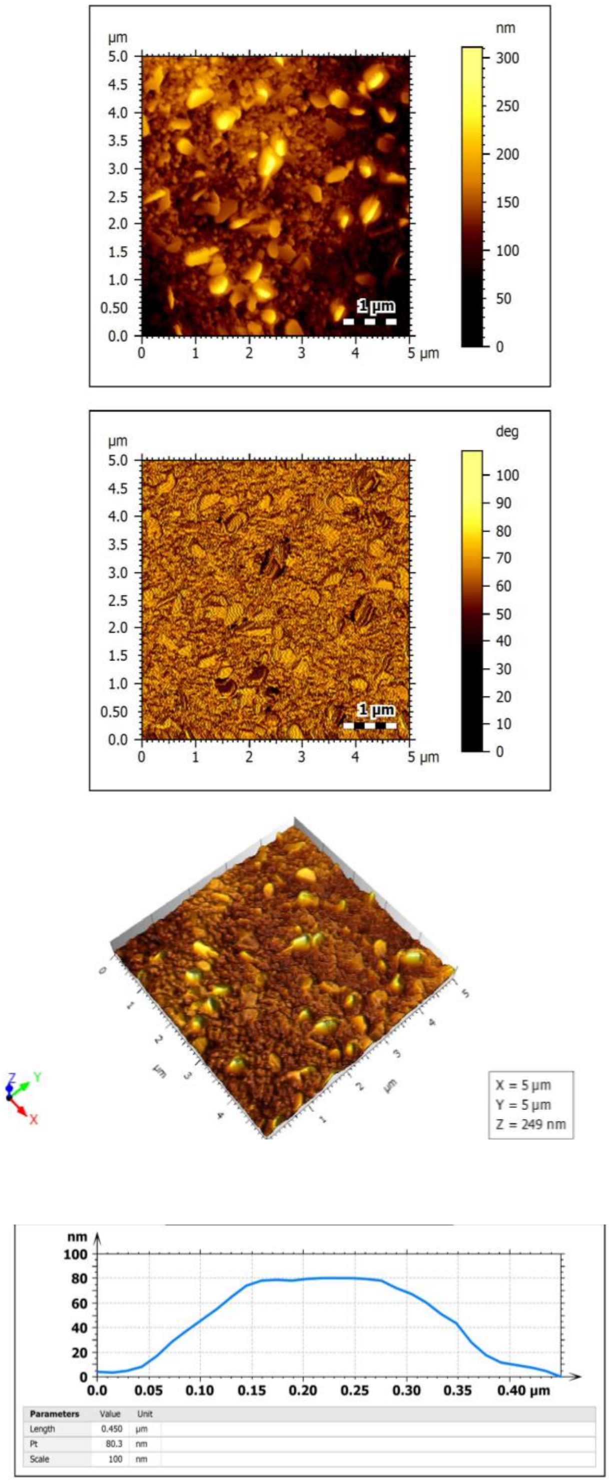

Figure S18. The tapping mode AFM images of DPAPMI of same sample (shown in previous AFM image) after grinding. From top to bottom represents height profile, morphology, 3D height profile and scale bar showing height (80.3 nm) image. This indicates that after grinding bulk material crushed into much smaller size probably due to enormous rupturing of mutable noncovalent interactions shown in crystal section in main manuscript.

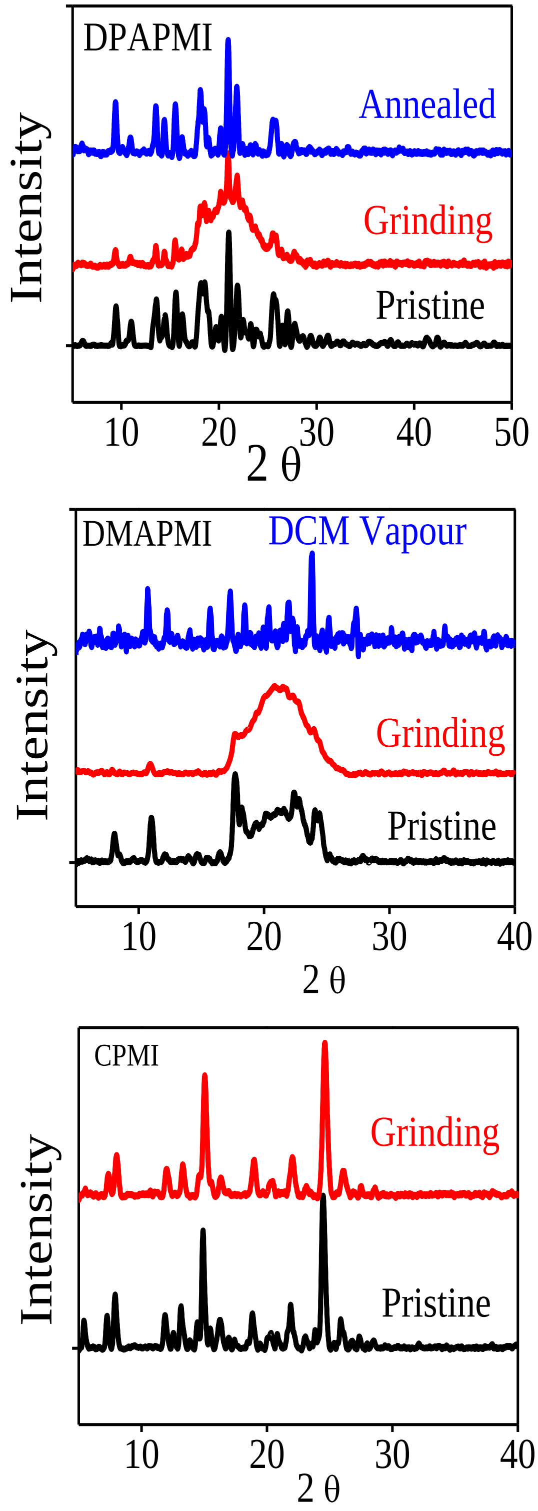

Figure S19: The PXRD pattern of each luminogens under different mechanical treatment.

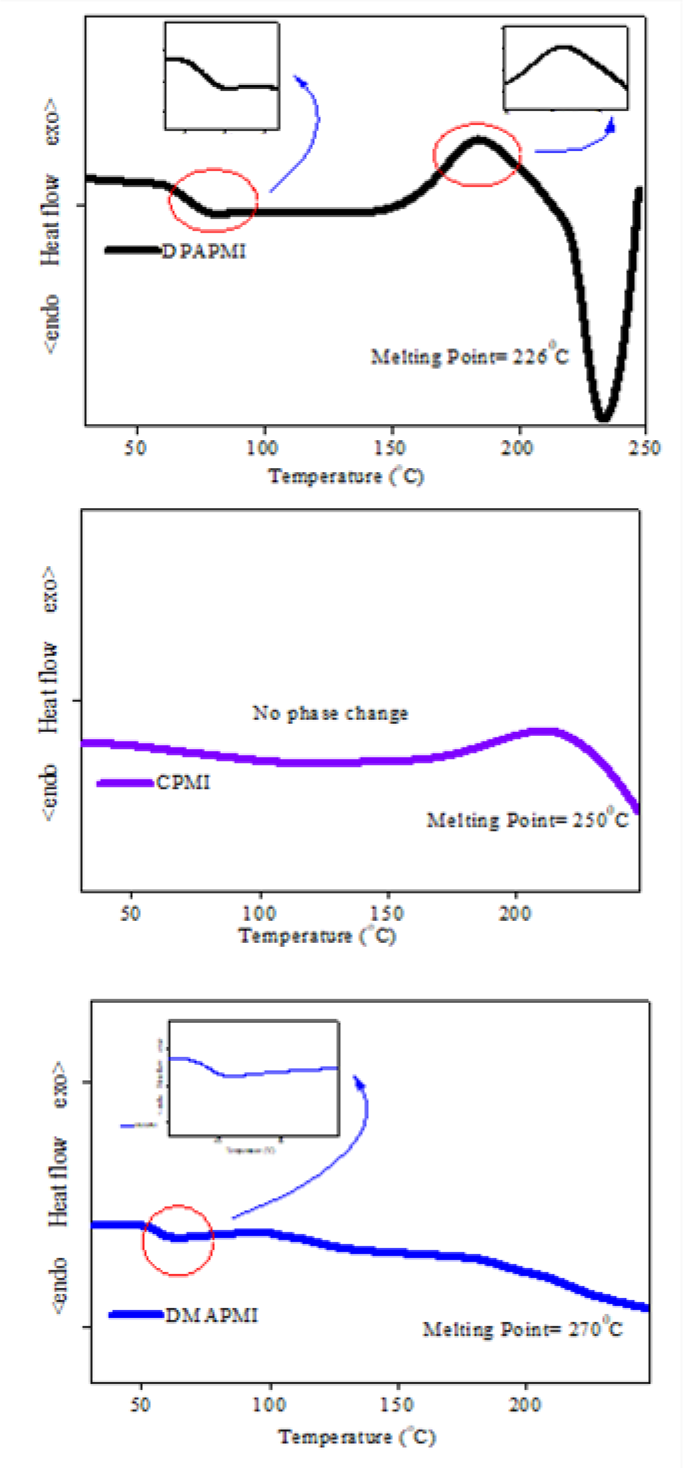

Figure S20: The DSC measurements of DPAPMI, CPMI and DMAPMI luminogens. Here during plot the first cycle of data has been removed, as they contain the prehistory of the sample. The DSC curve shows two transition states for DPAPMI and one transition state for DMAPMI, but no transition state shows in CPMI molecule.

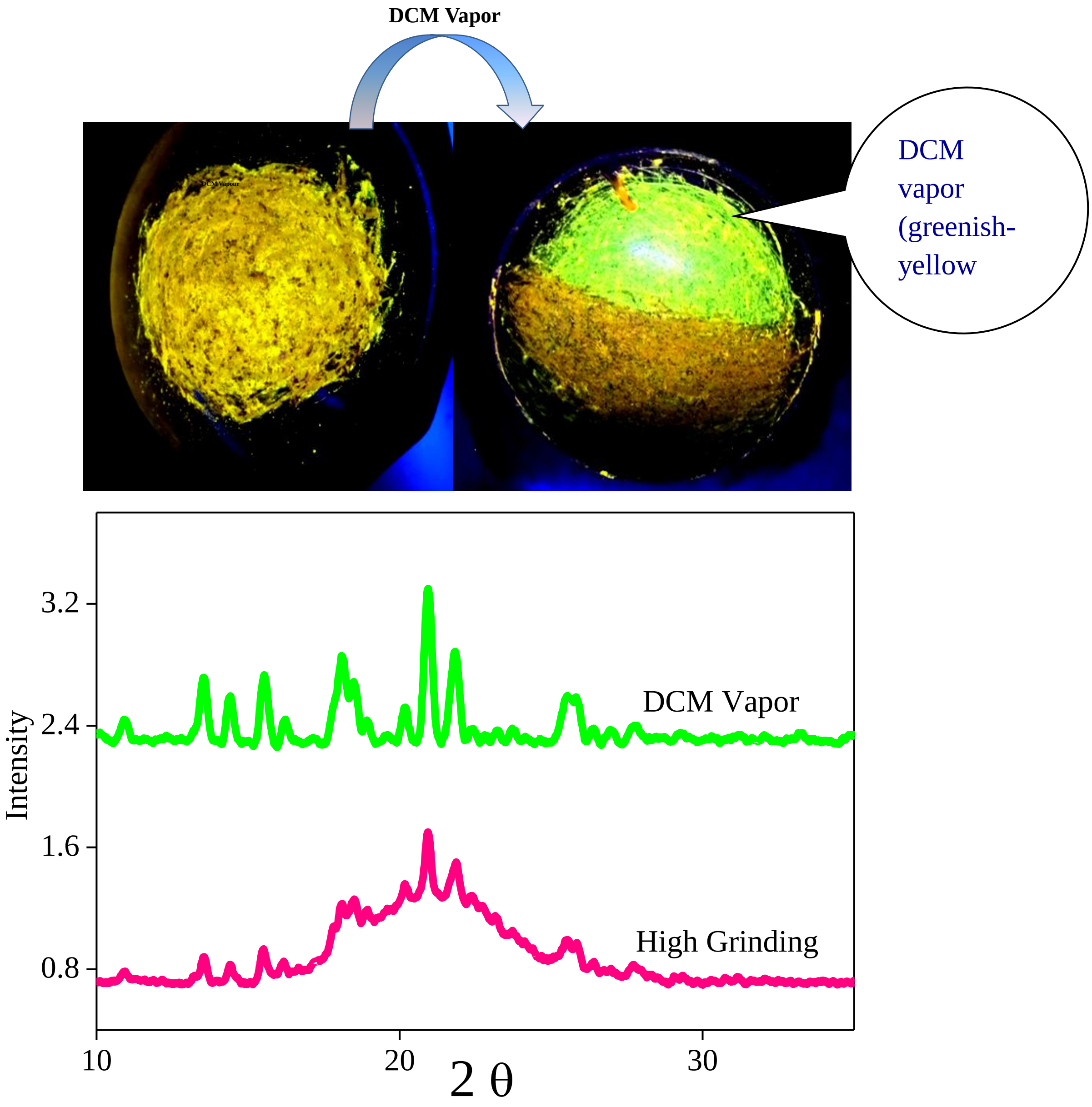

Figure S21. Grinded powder of DPAPMI (left) and under DCM vapor (right side greenish-yellow part) in presence of UV light. The color conversion was monitored instantly under DCM vapor. This experiment shows the ability of reversible switching of DPAPMI under external stimuli (DCM vapor). The PXRD measurement (bottom) shows semi-crystalline to crystalline transformation upon DCM vapor.

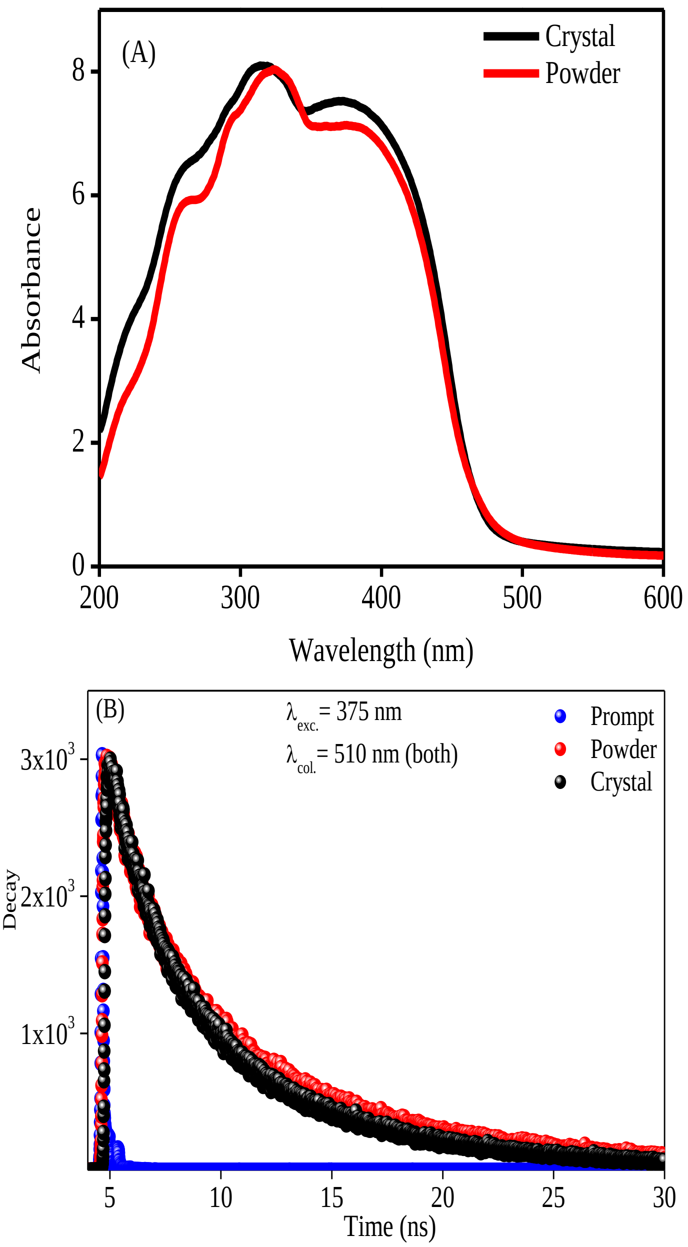

Figure S22: Absorption spectra of pristine powder and crystal of CPMI (A), time-resolved emission spectra of pristine powder and crystal of CPMI (B).

Hirshfeld surface mapped over shape index and curvedness:

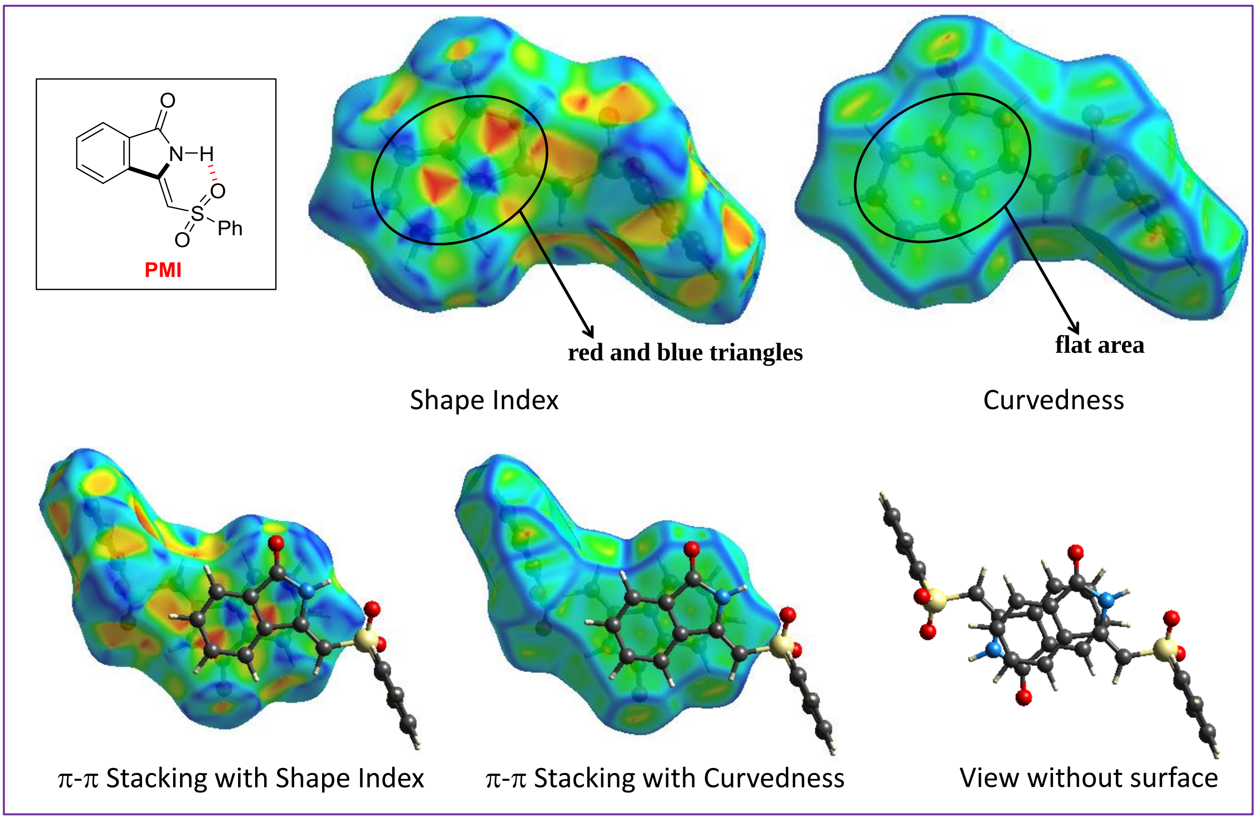

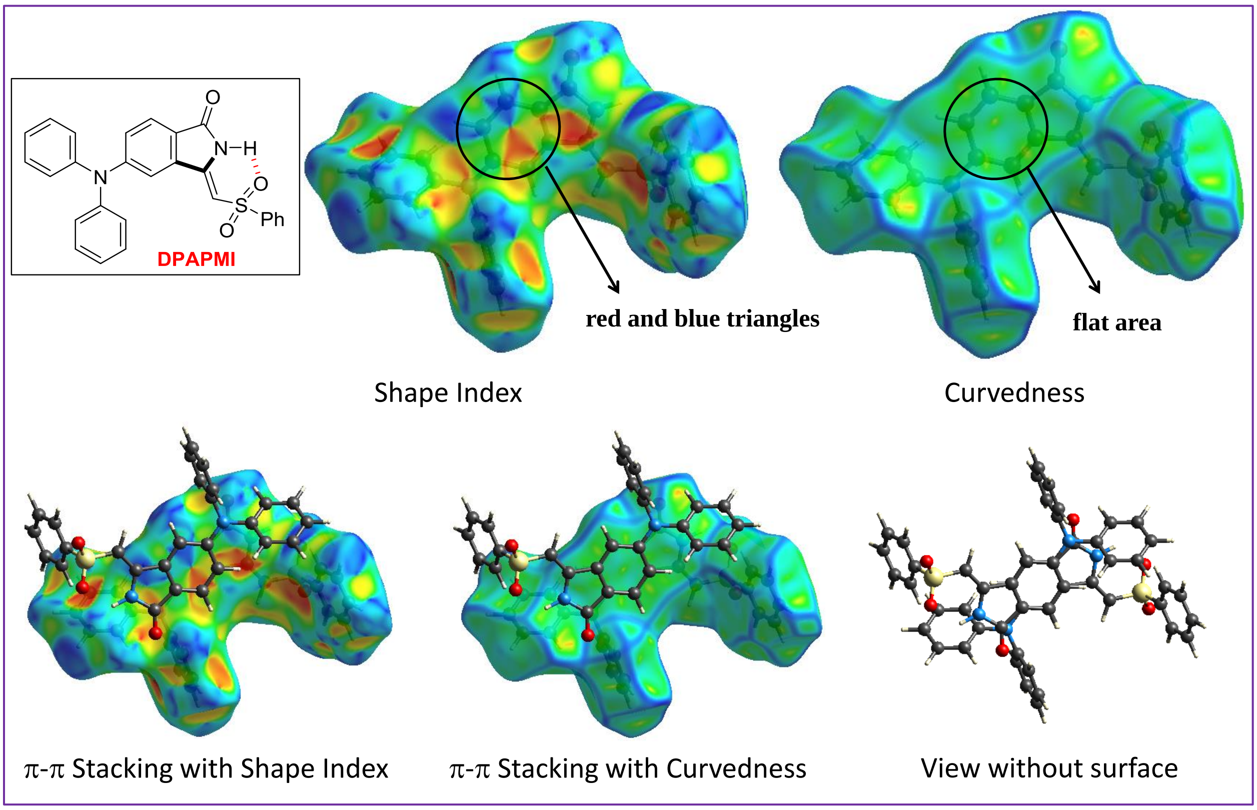

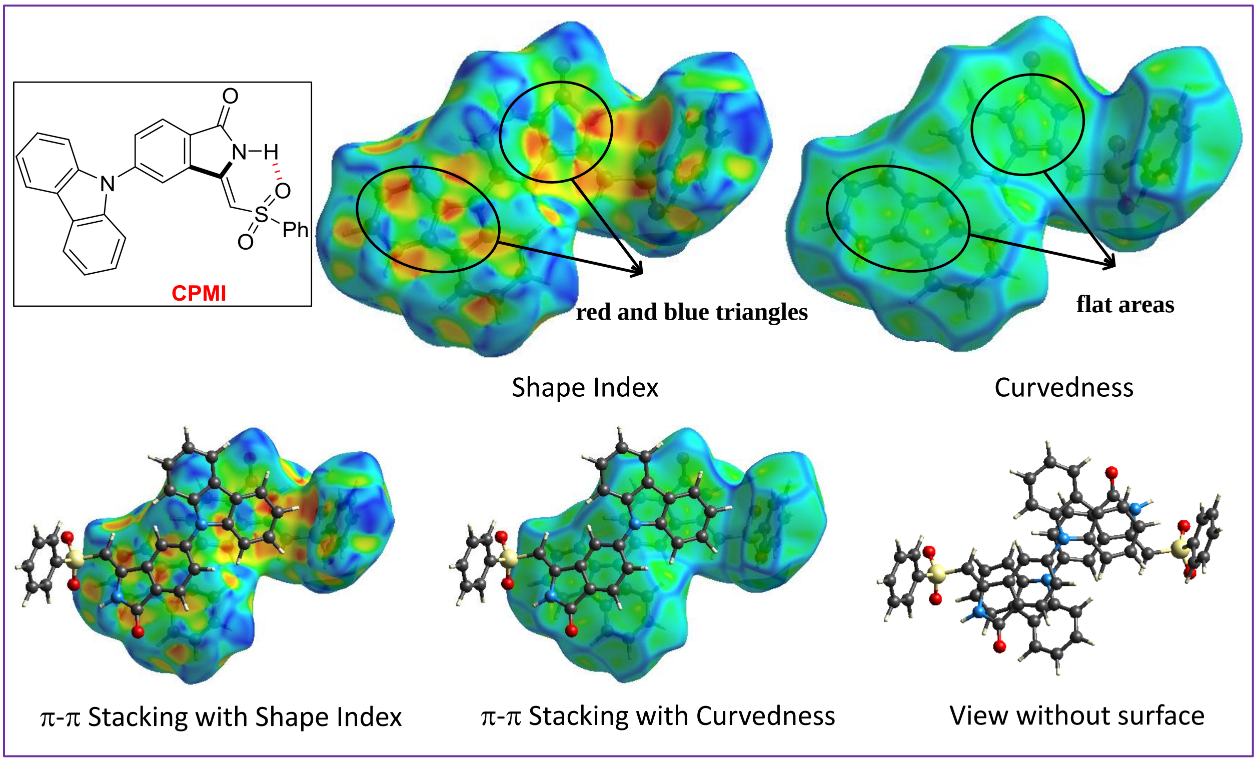

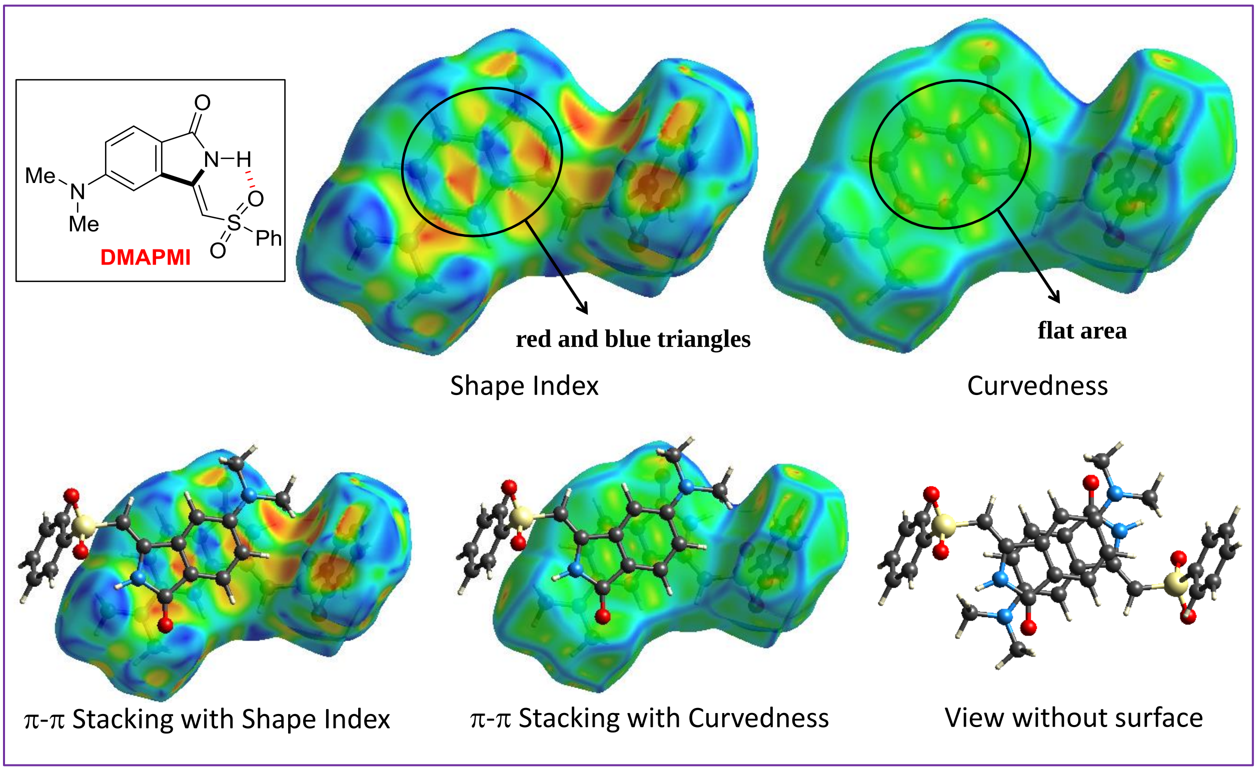

Figure S23: The Hirshfeld surface mapped over the curvedness and shape index of PMI, DPAPMI (previous page), CPMI and DMAPMI (current page). Here the red and blue triangles inside the circular region on shape index and flat region of curvedness indicate the π–π stacking region. For better clarification, we have summarized the π···π stacking region in a single image provided in Figure S24.

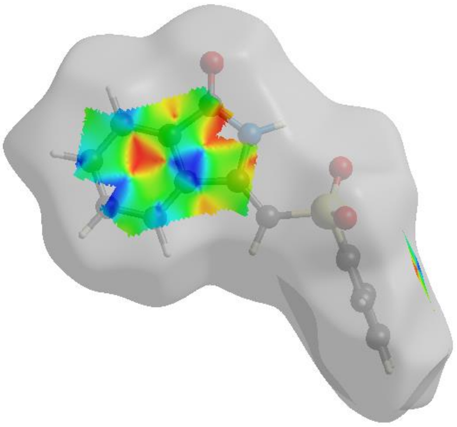

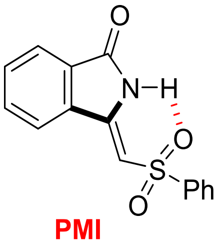

PMI

C-C (i.e. π–π) = 11.6%

π–π stacking both in five and six membered rings of isoindolinone core and aryl ring of -SO_2Ph group **(mechano-inactive)**

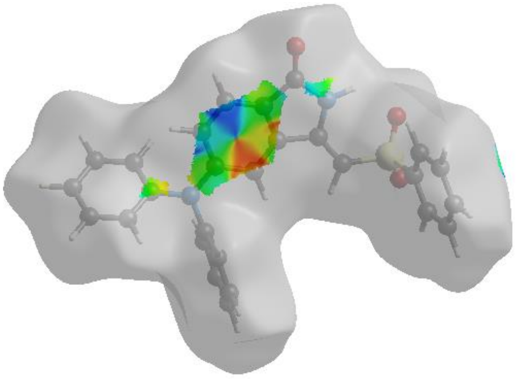

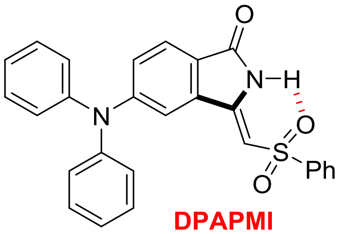

DPAPMI

C-C (i.e. π–π) = 4.1%

π–π stacking only in six membered ring of isoindolinone core **(mechanoactive)**

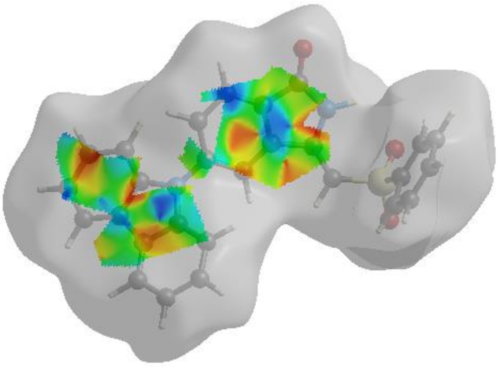

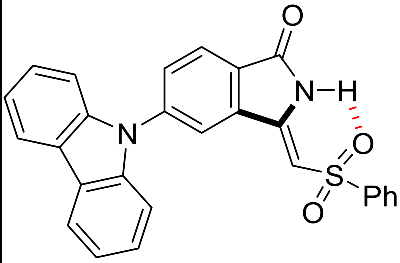

CPMI

C-C (i.e. π–π) = 12.8%

π–π stacking both in five and six membered rings of isoindolinone core and two rings of carbazole **(mechano-inactive)**

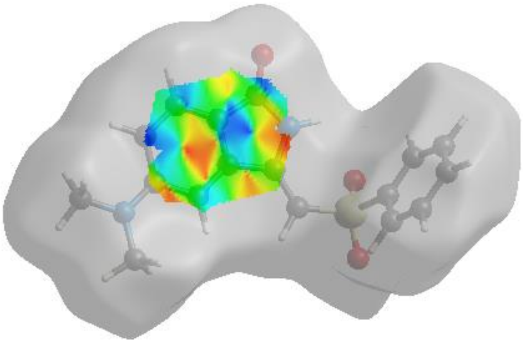

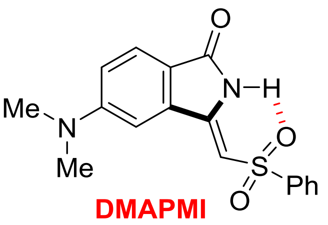

DMAPMI

C-C (i.e. π–π) = 4.4%

π–π stacking in five and six membered rings of isoindolinone core (**mechano-inactive)**

Figure S24: The Hirshfeld surface mapped over the π···π stacking region. Like earlier figure the red and blue triangles indicate the π–π stacking (other part omitted for clarity purpose). The % of π···π interactions are provided in Figure 7 in main manuscript.

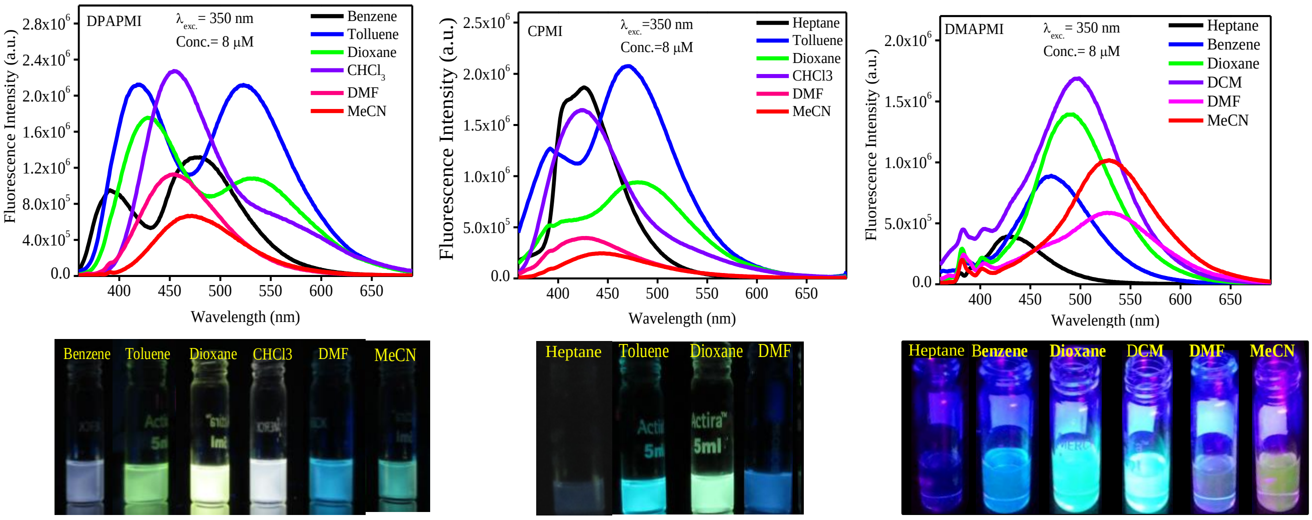

Figure S25: Steady state emission profiles of DPAPMI (left), CPMI (middle) and DMAPMI (right) in different solvents. Corresponding visual emission color has been provided in bottom row under the excitation of 365 nm UV light.

Table S1: Summerized steady-state parameters of each luminogen in different solvents.

Luminogens		PMI		DPAPMI		CPMI		DMAPMI	
Solvents	Δf (ϵ,ŋ)*	λ_a (nm)	λ_{em} (nm)	λ_a (nm)	λ_{em} (nm)	λ_a (nm)	λ_{em} (nm)	λ_a (nm)	λ_{em} (nm)
Heptane	0.001	315	360	-	-	315/ 375	430	310/ 370	430
Benzene	0.003	-	-	320/ 405	400/485	-	-	310/ 382	470
Tolluene	0.014	-	-	320/ 408	418/525	315/ 380	400/475	-	-
Dioxane	0.021	315	430	320/ 405	430/530	315/ 380	400/480	310/ 395	490
$CHCl_3$	0.149	315	430	320/ 420	455/560	315/ 384	420 & 545 (peeping peak)	-	-
THF	0.210	-	-	320/ 422	442/553	-	-	-	-
DCM	0.219	315	430	-	-	-	-	310/ 402	500
DMF	0.275	-	-	320/ 422	450	315/ 387	425	310/ 407	525 & 460 (minor)
MeCN	0.305	315	440	320/ 425	470	315/ 388	442	310/ 415	532

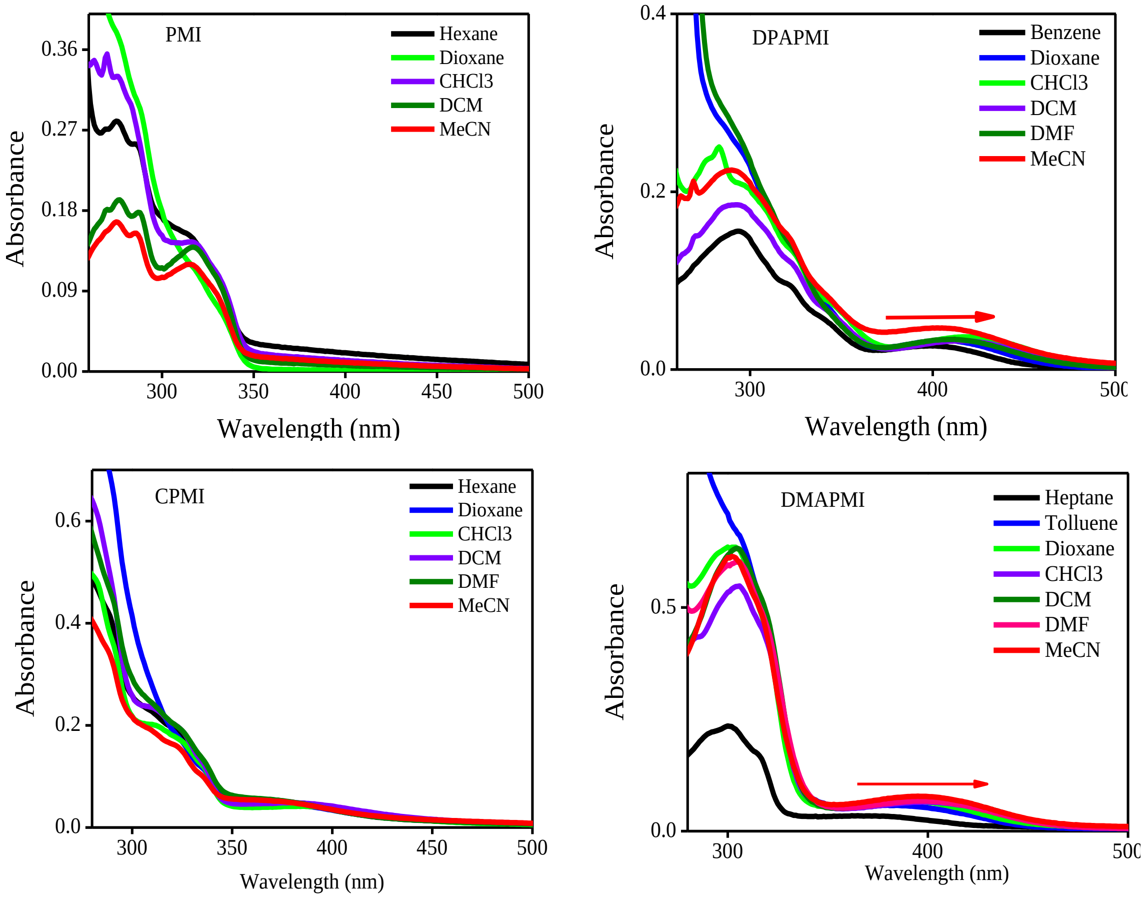

Figure S26: Absorption spectra of each luminogen in different solvents.

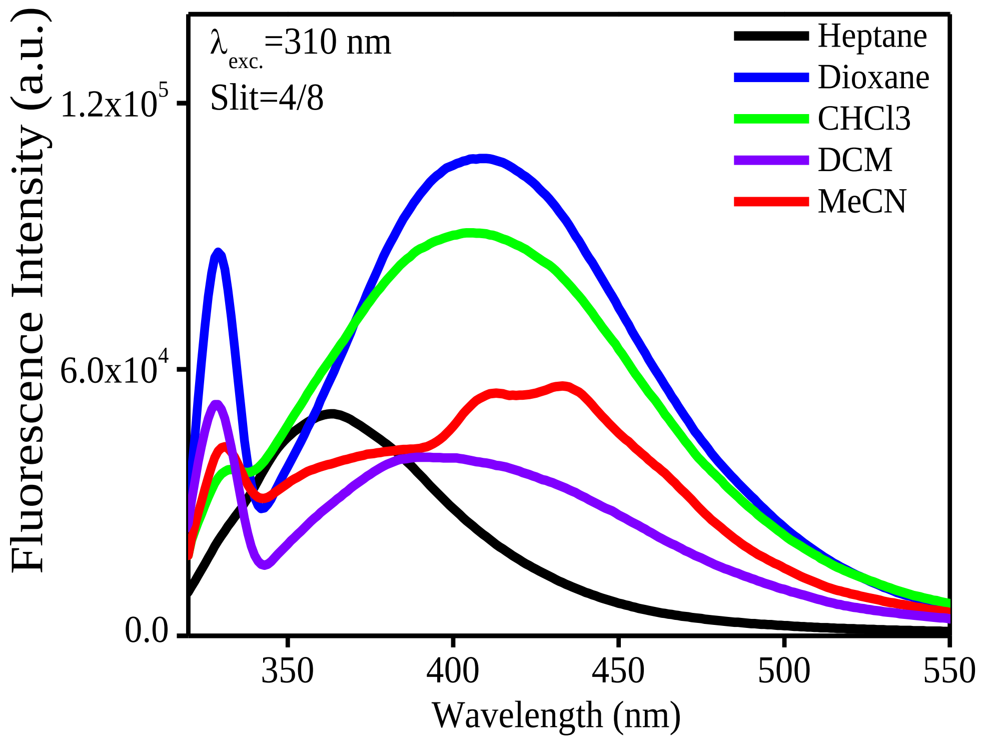

Figure S27: Emission spectra of PMI in different solvents varying polarity.

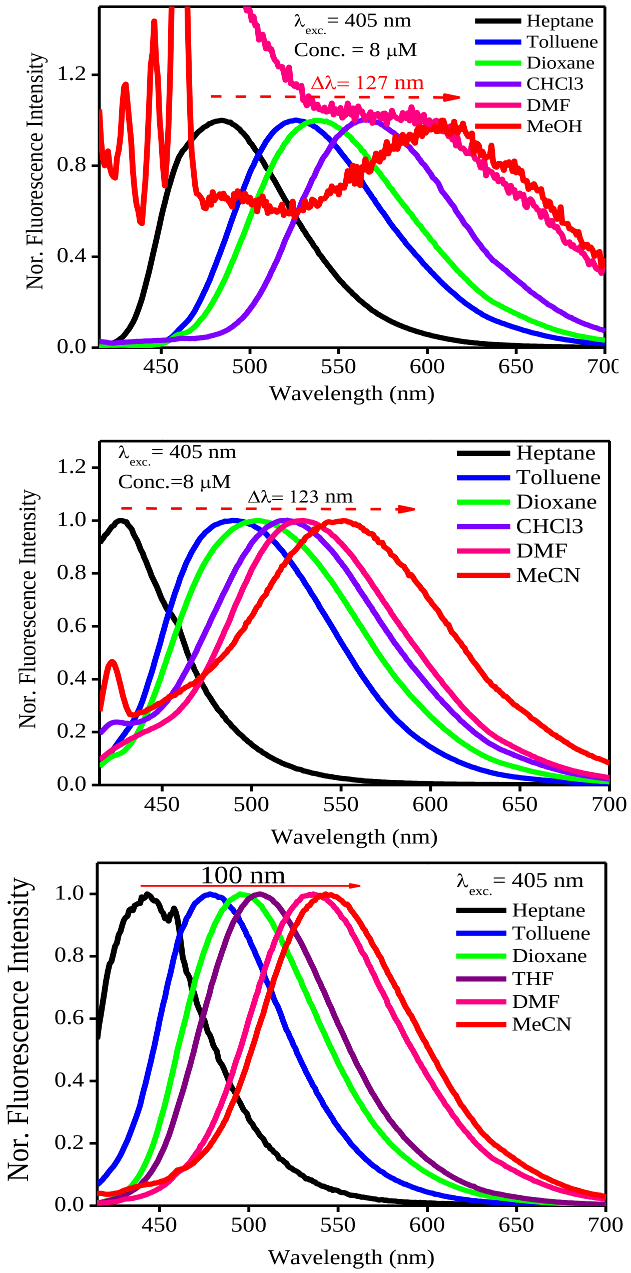

Figure S28: Normalised emission spectra of DPAPMI (top), CPMI (middle) and DMAPMI (bottom) in different solvent medium ($\lambda_{ex.}$ = 405 nm).

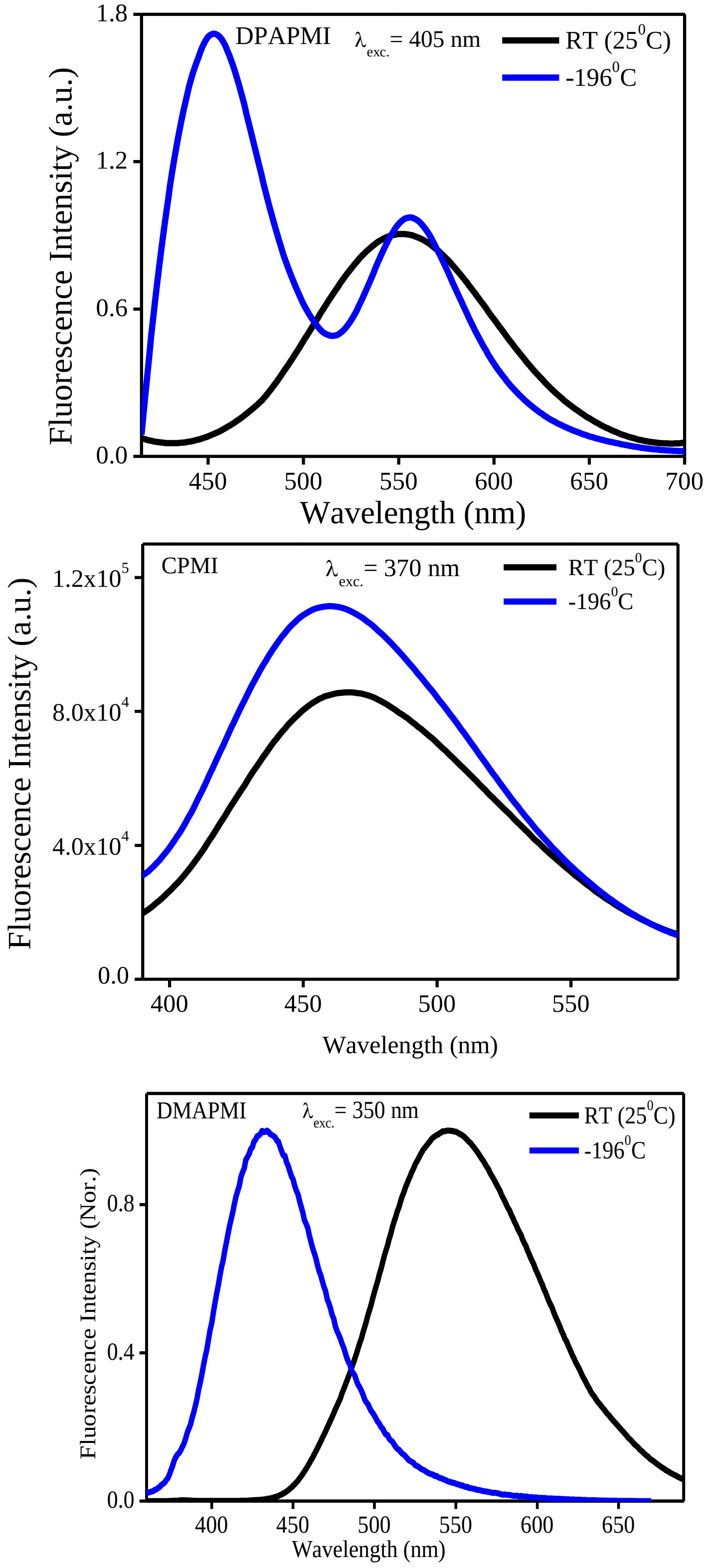

Figure S29: Temperature dependent fluorescence measurements of DPAPMI, CPMI and DMAPMI.

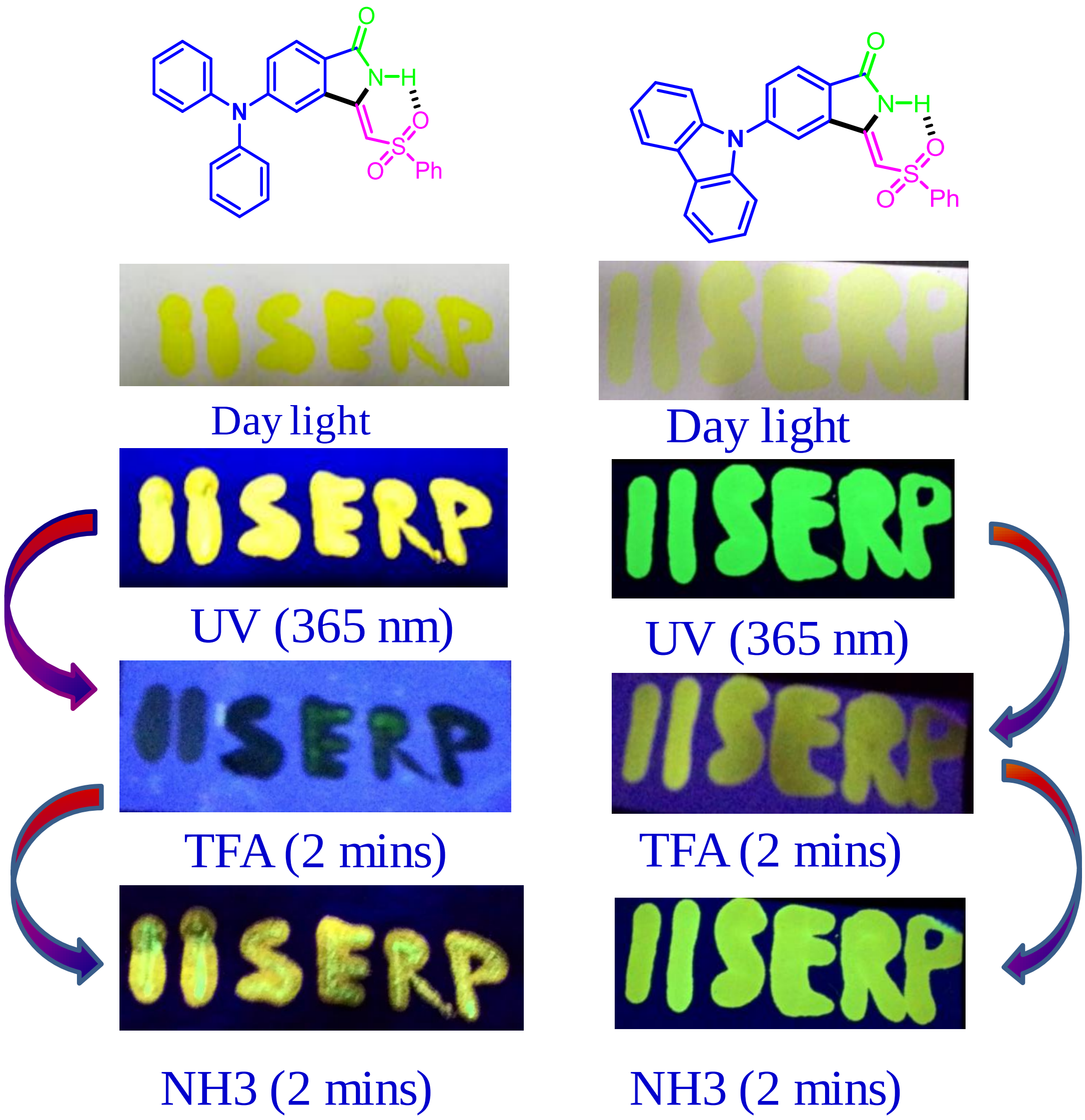

Figure S30: Representation of acid and base induced fluorescence off-on for the AIE active luminogen DPAPMI (left column) and CPMI (right column). After TFA exposure (2 mins) fluorescence is turned 'off' and fluorescence is reversibly switched 'on' under NH_3 exposure (2 mins) for both molecules. Images have taken under 365 nm UV light exposure.

References

(1) E. Ishow, A. Brosseau, G. Clavier, K. Nakatani, P. Tauc, C. Fiorini-Debuisschert, S. Neveu, O. Sandre and A. Léaustic, *Chem. Mater.*,2008,**20,** 6597.

(2) V. A.Galievsky, S. I. Druzhinin, A. Demeter, P. Mayer, S. A. Kovalenko,T. A. Senyushkina and K. A. Zachariasse, *J. Phys. Chem. A*, 2010, **114**, 12622.

(3) A. D. Martin, J. Britton, T. L. Easun, A. J. Blake, W. Lewis and M. Schröder, *Cryst. Growth Des.,* 2015, **15**, 1697.

(4) A. D. Martin, K. J. Hartlieb, A. N. Sobolev and C. L.Raston, *Cryst. Growth Des.,* 2010, **10**, 5302.

(5) J. J. McKinnon, D. Jayatilaka, M. A. Spackman, *Chem. Comm.*,2007, 3814.

(6) M. J. Turner, J. J. McKinnon, S. K. Wolff, D. J. Grimwood, P. R. Spackman, D. Jayatilaka and M. A. Spackman, *Crystal Explorer*17 (2017).
